# A stemness-based eleven-gene signature correlates with the clinical outcome of hepatocellular carcinoma

**DOI:** 10.1186/s12885-021-08351-0

**Published:** 2021-06-19

**Authors:** Liang Hong, Yu Zhou, Xiangbang Xie, Wanrui Wu, Changsheng Shi, Heping Lin, Zhenjing Shi

**Affiliations:** 1 Department of Infectious, The Third Affiliated Hospital of Wenzhou Medical University, Ruian, Zhejiang 325200 People’s Republic of China; 2 Department of Interventional, The Third Affiliated Hospital of Wenzhou Medical University, Ruian, Zhejiang 325200 People’s Republic of China; 3 Department of Respiratory, The Third Affiliated Hospital of Wenzhou Medical University, Ruian, Zhejiang 325200 People’s Republic of China

**Keywords:** Stemness, Hepatocellular carcinoma, Gene signature, Molecular subtype, Prognosis

## Abstract

**Background:**

Cumulative evidences have been implicated cancer stem cells in the tumor environment of hepatocellular carcinoma (HCC) cells, whereas the biological functions and prognostic significance of stemness related genes (SRGs) in HCC is still unclear.

**Methods:**

Molecular subtypes were identified by cumulative distribution function (CDF) clustering on 207 prognostic SRGs. The overall survival (OS) predictive gene signature was developed, internally and externally validated based on HCC datasets including The Cancer Genome Atlas (*TCGA*), GEO and ICGC datasets. Hub genes were identified in molecular subtypes by protein-protein interaction (PPI) network analysis, and then enrolled for determination of prognostic genes. Univariate, LASSO and multivariate Cox regression analyses were performed to assess prognostic genes and construct the prognostic gene signature. Time-dependent receiver operating characteristic (ROC) curve, Kaplan-Meier curve and nomogram were used to assess the performance of the gene signature.

**Results:**

We identified four molecular subtypes, among which the C2 subtype showed the highest SRGs expression levels and proportions of immune cells, whereas the worst OS; the C1 subtype showed the lowest SRGs expression levels and was associated with most favorable OS. Next, we identified 11 prognostic genes (CDX2, PON1, ADH4, RBP2, LCAT, GAL, LPA, CYP19A1, GAST, SST and UGT1A8) and then constructed a prognostic 11-gene module and validated its robustness in all three datasets. Moreover, by univariate and multivariate Cox regression, we confirmed the independent prognostic ability of the 11-gene module for patients with HCC. In addition, calibration analysis plots indicated the excellent predictive performance of the prognostic nomogram constructed based on the 11-gene signature.

**Conclusions:**

Findings in the present study shed new light on the role of stemness related genes within HCC, and the established 11-SRG signature can be utilized as a novel prognostic marker for survival prognostication in patients with HCC.

**Supplementary Information:**

The online version contains supplementary material available at 10.1186/s12885-021-08351-0.

## Background

Globally, liver cancer is the fourth most lethal cancer worldwide [1]. Hepatocellular carcinoma (HCC) is rank as the major histological subtype (approx. 70–85% cases) of total liver cancer cases. The prognosis of advanced HCC is still not satisfactory and treatment options are limited [2]. Integrative studies combining transcriptome and genomic analysis have confirmed that HCC has much heterogeneous at the histo-molecular level and clinical outcomes, and the molecular diversity of HCC is tightly associated with different aetiologies and distinct mechanisms of hepato-carcinogenesis [2]. Give that only individually tailored molecular profiles and biomarkers could escape the patients from undergoing a potentially more harmful, aggressive chemical therapy or even leave them untreated, we should illustrate the natural history of HCC in individual patients by clearly understood their personal molecular characteristics. Therefore, there is an increasing interest in the molecular characterization of HCC allowing prognosticate overall patient survival.

The biological similarity of cancer cells and stem cells has been well documented, and abnormal stem cells is supposed to play an important role in HCC progression [3]. Although several highly conserved pathways including Notch, Hedgehog, Hypoxia and Wnt signaling pathways are pivotal for maintaining self-renewal in cancer stem cells (CSCs) and thus involved in tumorigenesis and cancer development [4], almost nothing is known about the precise role and underlying mechanism of stemness related genes (SRGs) and their gene expression profiles in primary HCC, not yet anything known related to the prognostic distinctions of HCC.

Here we aimed at explore new prognostic signature in patients with HCC using Cox and the least absolute shrinkage and selection operator (LASSO) regression models to analyze the expression profile of stemness-related genes (SRGs) using multiple online HCC dataset. Based on SRG expression data from public databases in The Cancer Genome Atlas (*TCGA*), we constructed molecular subtypes with distinct different immune characteristics and clinical outcomes. Then, we developed a 11-gene signature for assessing the prognosis of patients with HCC, and further validated it in *TCGA,* Gene Expression Omnibus (GEO) and International Cancer Genome Consortium (ICGC) HCC datasets. This module was closely related to patients’ prognosis and could apply as an independent pathological predictor.

## Methods

### Patients and datasets

The clinical information and RNA-seq data of 342 HCC samples were download from *TCGA* database (http://www.cancer.gov/about-nci/organization/ccg/research/structural-genomics/*TCGA*). A dataset which contains gene expression profiles of HCC samples in GEO database, GSE15654 (contains 216 HCC samples), was downloaded from NCBI (http://www.ncbi.nlm.nih.gov/geo/). The RNA-seq data and clinical information of 212 HCC samples in the ICGC HCC cohort was download from ICGC Data Portal (https://icgcportal.genomics.cn/). The clinicopathological characteristics of patients from these three datasets after preprocessing are summarized in Table 1. For *TCGA* HCC dataset, 50% of them was randomly divided into training cohort (*n* = 171), and the rest 171 cases and the entire dataset were selected as internal validation cohorts. GSE15654 and ICGC HCC datasets were used as external validation cohorts. Patient informed consent was existing in these three public datasets, and this study was approved by the Institutional Review Boards of the Third Affiliated Hospital of Wenzhou Medical University.

**Table 1:** Clinical and pathologic characteristics of patients in the pre-processed *TCGA* and GEO HCC datasets

Characteristic		Training Set (*n* = 171)	Validation Set (*n* = 171)	*P* value*	ICGC HCC dataset (*n* = 212)	GSE15654 dataset (*n* = 216)
Age (years)	≤60	81	84	0.828	43	–
	> 60	90	87		169	–
Survival Status	Living	109	110	1	176	150
	Dead	62	61		36	66
Sex	Female	55	54	1	50	–
	Male	116	117		162	–
Grade	G1	24	29	0.513	–	–
	G2	82	79		–	–
	G3	59	52		–	–
	G4	4	8		–	–
Pathologic T stage	T1	79	89	**0.028**	–	–
	T2	54	30		–	–
	T3	43	41		–	–
	T4	4	9		–	–
Pathologic N stage	N0	120	119	0.934	–	–
	N1/Nx	50	52		–	–
Pathologic M stage	M0	128	116	0.188	–	–
	M1/Mx	43	55		–	–
Tumor Stage	Stage I	78	83	**0.011**	33	–
	Stage II	50	27		102	–
	Stage III	34	46		61	–
	Stage IV	1	2		16	–

*Chi-Squared test. ICGC, International Cancer Genome Consortium

### Identification of molecular subtypes based on SRGs

A total of 456 genes related to stemness from 30 stemness-related gene sets (Supplementary Table S1) were collected from the Molecular Signature Database v7.0 (MSigDB). Among them, 48 genes not offered in *TCGA* HCC dataset or with FPKM = 0 in more than half of the samples were excluded. Finally, 408 genes were enrolled for subsequent analysis. Prognostic genes were detected by univariate Cox regression survival analysis using the R package survival coxph function, and log rank *P* < 0.05 was selected as the threshold. The molecular subtypes were identified based on these prognostic genes using cumulative distribution function (CDF) method, and the optimal number of subtypes were determined according to the CDF Delta area.

### Identification of hub genes by protein-protein interaction (PPI) analysis

Since protein-protein interaction (PPI) analysis can help identify hub genes with core functions, PPI among genes in the identified key modules was further explored. The Search Tool for the Retrieval of Interacting Genes (STRING) is a well-known database containing comprehensive PPI information (version 11.0, https://string-db.org/). The PPI network of these genes was thus mapped to the STRING assembly and then visualized by the Cytoscape software. Important nodes in the network was identified by the Cytoscape plugin *cytoHubba* [5]. The topological analysis method Degree and the centrality analysis methods Closeness and Betweenness were used separately to identify the hub nodes in the PPI network. Among the top 10 hub nodes identified by each method, only genes with consistent high Degree, Closeness, and Betweenness values (larger than the median value) were selected as hub genes.

### Construction of stemness-related prognostic gene signature

Co-expression genes in the training set were detected by univariate Cox regression survival analysis, and log rank *p* < 0.01 was selected as the threshold. To narrow the gene range and maximize the accuracy, LASSO Cox regression analysis [6], a method screening signatures with generally effective prognostication performance by performing automatic feature selection, was performed by using the glmnet package of R to identify the prognostic gene. Optimal genes were evaluated by 10-fold cross validation. Risk score for each patient of the training set was calculated with the linear combinational of the signature gene expression weighted by their regression coefficients. Risk score = (expr_gene1_ x coefficient_gene1_) + (expr_gene2_ x coefficient_gene2_) + … + (expr_genen_ x coefficient_genen_). Receiver operating characteristics (ROC) curves, carried out by using the R package timeROC, was used to analyze the risk score of each sample, and samples were set as high-risk group or low-risk group by set the threshold as 0.

### Bioinformatic analysis

The enumeration of six tumor-infiltration immune cells (B cell, CD4^+^ T cell, CD8^+^ T cell, neutrophil, macrophage, neutrophils and dendritic cell) was estimated using the “Tumor Immune Estimation Resource” (TIMER, https://cistrome.shinyapps.io/timer/) tool [7]. Single-sample Gene set enrichment analysis (ssGSEA) was applied for identifying relationship between the risk scores of different samples and biological functions using the R package GSVA. The classical gene sets of Kyoto Encyclopedia of Genes and Genomes (KEGG) pathways (c2. cp. kegg. v11.0.symbols) were considered to decipher the phenotype. For each analytical pathway, the enrichment score (ES) and the significance of ES were calculated, and the normalized enrichment score (NES) and false discovery rate (FDR) were further calculated to examine functional enrichment results. A FDR cutoff value of 0.05 was considered in this test.

### Statistical analysis

DESeq2 was used to calculate differentially expressed genes (DEGs) among each cluster (FDR < 0.05 and |log2FC| > 1). Kaplan-Meier curves were applied to assess the difference on OS between different groups. Multivariate Cox regression analyses were performed to assess the independent prognostic factors. Decision curve analysis (DCA), which can evaluate predictive models from the perspective of clinical consequences [8], was performed in the entire cohort to test the clinical usefulness of the nomogram in comparison with the gene signature and clinicopathological parameters. All statistical analyses were using R 3.6.0 (https://mirrors.tuna.tsinghua.edu.cn/CRAN/) with default software parameters. *P* value < 0.05 was considered significant statistically.

## Result

### Identification of molecular subtypes in HCC

**Fig. 1:**
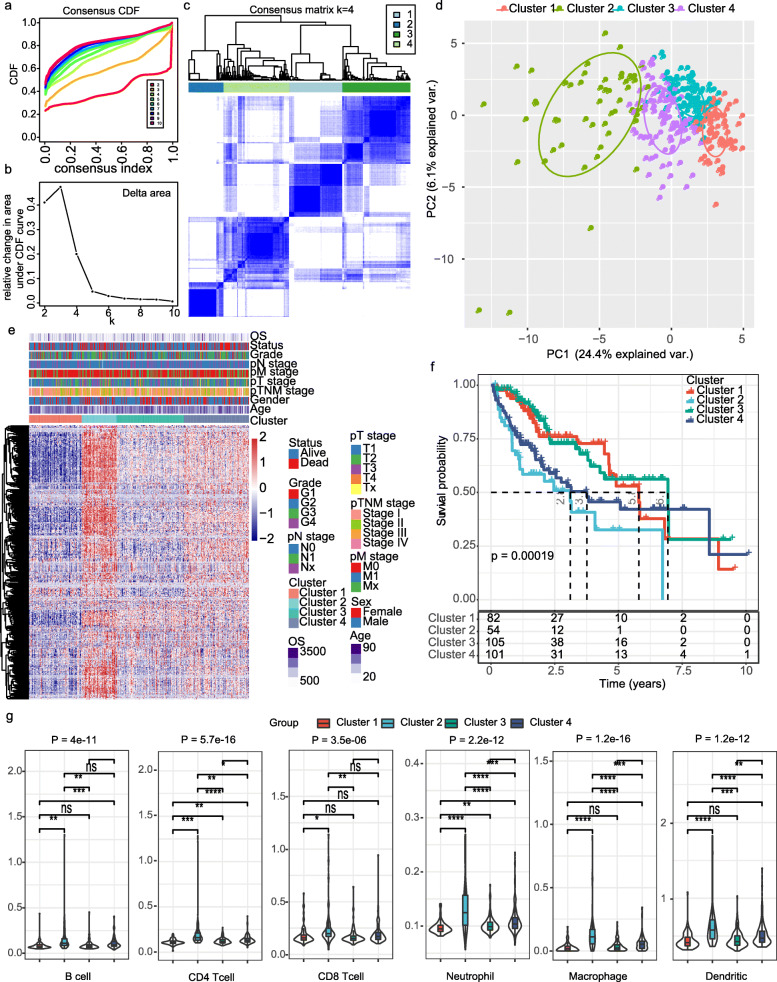
Identification of molecular subtypes in HCC. **a** cumulative distribution function (CDF) curve of K = 2–10; **b** The relative change in area under the CDF curve of K = 2–10; **c** A relative stable partition of the samples is found at K = 4. **d** PC analysis for K = 4 are shown; **e** Heat map of the expression profile of 408 SRGs and the distribution of clinicopathological parameters in all four subtypes; **f** Kaplan-Meier curves with log rank analysis showed the overall survival (OS) curve of the four subtypes; **g** The proportions of B cell, CD4^+^T cell, CD8^+^T cell, Neutrophil, Macrophage, and Dendritic cell (DC) in the three subtypes

By univariate Cox regression survival analysis, 207 stemness related genes (SRGs) were identified correlated with the overall survival (OS) of patients with HCC in the *TCGA* dataset (Supplementary Table S2). Consensus unsupervised clustering of 342 samples from HCC patients, we found that 4 clusters had lower values of ambiguously clustered pairs (PAC), which reflected the near-perfect stability of the samples under the correct K value distribution (Fig. 1a-b). The relative change of the area under the CDF curve reveals a nearly perfect stable distribution of the samples starting from 4 clusters (Fig. 1c). Principal component analysis (PCA) and consensus heatmaps also showed a relatively stable distribution samples in the 4 clusters (Fig. 1d-e). After evaluating the relative changes in the area under the CDF curve, PAC value, PCA and consensus heatmaps, we chose a four-cluster solution. Thus, four molecular subtypes (Cluster 1 [*n* = 82], Cluster 2 [*n* = 54], Cluster 3 [*n* = 105]) and Cluster 4 [*n* = 101]) were constructed based on these 207 prognostic genes (Fig. 1a-e).

Gene expression profile and the distribution of clinicopathological parameters in each subtype was showed in Fig. 1e. However, the molecular subtype had no correlation with any clinicopathological features of patients with HCC (Supplementary Figure 1). Kaplan–Meier method with log-rank tests were applied to explore the difference of prognosis among the four molecular subtypes in HCC. Compared with Cluster 1 and Cluster 3, patients in Cluster 2 and Cluster 4 showed worse overall survival (OS) time (Fig. 1f). In addition, the differences on immune characteristics among the three subtypes were analyzed. Cluster 2 showed the highest proportions of immune cell infiltration than the other three subtypes (Fig. 1g).

### Selection of hub genes by intersection and PPI analyses

Genes differentially expressed among each molecular subtype were calculated (Table 2). There are 286 differentially expressed genes (DEGs, 272 up-regulated and 14 down-regulated, Supplementary Figure 2A) in Cluster 1 compared with the other three subtypes, 727 DEGs (294 up-regulated and 433 down-regulated, Supplementary Figure 2B) between Cluster 2 and other three subtypes, 276 DEGs (264 up-regulated and 12 down-regulated, Supplementary Figure 2C) between Cluster 3 and other three subtypes, and 56 DEGs (31 up-regulated and 25 down-regulated, Supplementary Figure 2D) between Cluster 4 and other three subtypes. The up-regulated and down-regulated DEGs were depicted on Venn diagram (Supplementary Figure 2E-F).

**Table 2:** DEGs in each Cluster

	Cluster 1	Cluster 2	Cluster 3	Cluster 4
Upregulated DEGs	272	294	264	31
Downregulated DEGs	14	433	12	25
All DEGs	286	727	276	56

*DEGs* differentially expressed genes

**Fig. 2:**
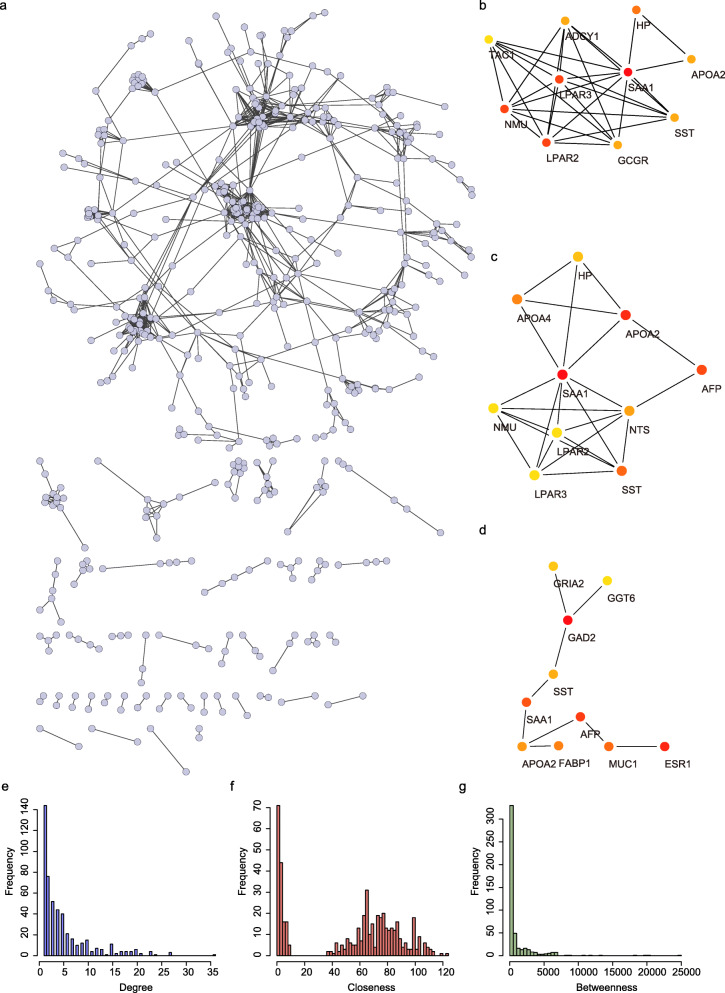
Screening of hub genes involved in the development of STAD. **a** The network showed co-expression gene in PPI pairs with a score higher than 0.7; **b**-**d** Top hub genes identified by the Degree (**b**), Closeness (**c**), and Betweenness (**d**); **e**-**g** The topological properties of the PPI network and the distributions of degree (**e**), closeness (**f**), and betweenness (**g**)

Considering that gene interaction networks helps to uncover key genes participate in liver cancer progression, we mapped the expression of the 1345 DEGs to STRING database to construct PPI network. A total of 1216 PPI pairs with a score higher than 0.7 were matched (Fig. 2a, Supplementary Table S3). Among which, the top 490 hub genes identified by the Degree (Fig. 2b), Closeness (Fig. 2c), and Betweenness (Fig. 2d) methods were largely consistent (Supplementary Table S4). The topological properties of the PPI network were also evaluated and the distributions of degree, closeness, and betweenness were shown in Fig. 2e-g. A total of 130 genes that satisfied high degree, closeness, and betweenness scores were selected out as hub genes for further analysis (Supplementary Table S5). These hub DEGs were assumed to be strongly correlated with the development of HCC, and were enrolled for subsequently identification of prognostic gene.

### Construction of prognosis risk model based on differential co-expression genes

**Fig. 3:**
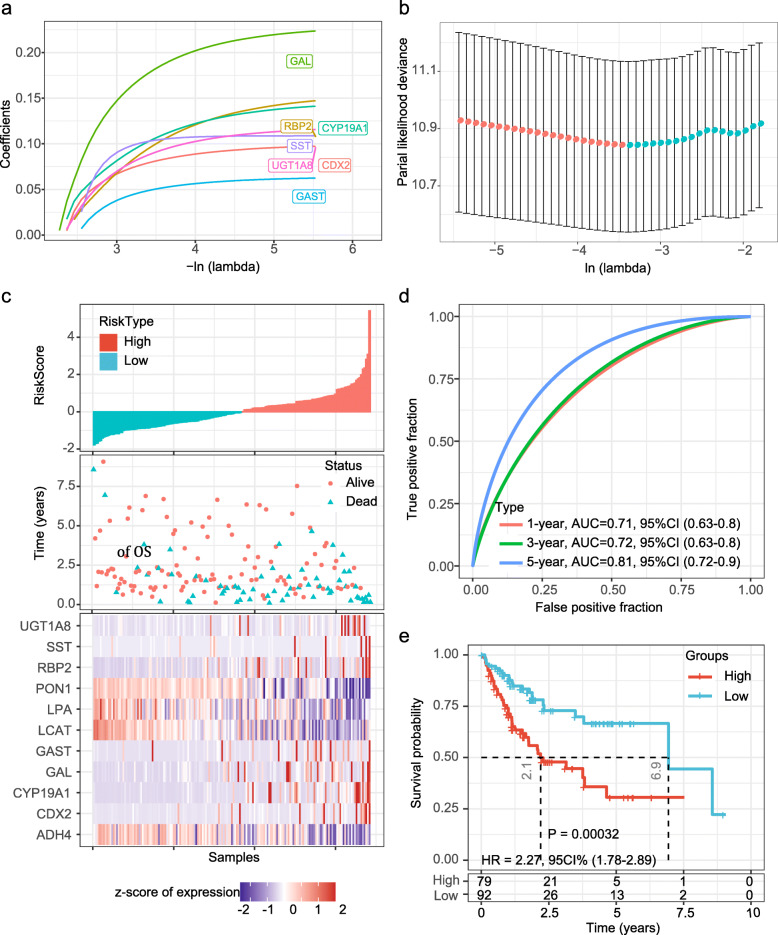
Evaluation of the performance of the 11-gene signature in the training dataset. **a** Trajectory change of each independent variable, the X axis represents the log value of the independent variable lambda, the Y axis represents the coefficient of the independent variable; **b** Confidence intervals of each lambda; **c** Risk score, survival time, survival status and expression of the 11-gene signature in the training set. **d** ROC curve of the 11-gene signature for OS in the training set. **e** Kaplan-Meier survival analysis of OS for patients in the training set. AUC, area under the curve; HR, hazard ratio; CI, confidence interval

The clinical information of HCC patients in the *TCGA* training (*n* = 171), *TCGA* testing (*n* = 171), and two external validation sets used for model construction and evaluation was listed in Table 1. To identify novel prognostic marker for patients with HCC, we applied univariate Cox proportional hazard regression and dimensional-reduction analysis by performing LASSO regression analysis to these 130 hub DEGs in the training set. And then 11 genes (including CDX2, PON1, ADH4, RBP2, LCAT, GAL, LPA, CYP19A1, GAST, SST and UGT1A8) significantly correlated to OS (*P* < 0.01, Table 3) were confirmed with 10-fold cross-validation and the minimized error rate λ = 0.034 (Fig. 3a-b). Among them, CDX2, RBP2, GAL, CYP19A1, GAST, SST and UGT1A8 showed significant negative correlation with OS, while the other four gens, PON1, ADH4, LCAT and LPA, were positive correlated to OS. The final 11-gene signature was calculated using Multivariate Cox survival analysis (Table 3), and a gene-based prognostic model was established to evaluate the survival risk of each patient, the formula of the gene signature is as follows: RiskScore = 0.079 * exp^CDX2^–0.203 * exp^PON1^–0.021 * exp^ADH4^ + 0.094 * exp^RBP2^ + 0.297 * exp^LCAT^ + 0.177 * exp^GAL^ + 0.037 * exp^LPA^ + 0.102 * exp^CYP19A1^ + 0.048 * exp^GAST^ + 0.102 * exp^SST^ + 0.087 * exp^UGT1A8^.

**Table 3:** Univariate Cox regression analysis result of 11 genes in the training set

Gene	*P* value	Hazard ratio	Low 95%CI	High 95%CI	Coefficient
CDX2	0.006	1.213	1.056	1.394	0.193
PON1	0.000	0.657	0.534	0.807	−0.421
ADH4	0.004	0.709	0.562	0.896	−0.343
RBP2	0.008	1.277	1.066	1.529	0.244
LCAT	0.001	0.634	0.479	0.839	−0.456
GAL	0.006	1.353	1.090	1.679	0.302
LPA	0.008	0.687	0.519	0.908	−0.376
CYP19A1	0.006	1.339	1.089	1.648	0.292
GAST	0.010	1.290	1.063	1.565	0.254
SST	0.002	1.430	1.140	1.792	0.357
UGT1A8	0.010	1.188	1.043	1.353	0.172

Based on the risk score formula and the cut-off value of normalized risk score (Z-score = 0), patients were divided into high-risk or low-risk group (Fig. 3c). And a heatmap shown the expression profile of the eight genes illustrated that as the risk score of patients increased, the expression of prognosis-risky genes (CDX2, RBP2, GAL, CYP19A1, GAST, SST and UGT1A8) were distinctly upregulated; in contrast, the expression of prognosis-protective gene (PON1, ADH4, LCAT and LPA) were downregulated. ROC curve showed that the accuracy of the 11-gene signature for one-year, three-year and five-year survival were greater than 0.70 (Fig. 3c). Finally, we divided the samples into the high-risk group (*n* = 79) when their Zscore-based Riskscore greater than zero, and the others as the low-risk group (*n* = 92). Kaplan-Meier curve analysis revealed that high-Riskscore confers significantly shorter OS time (HR = 2.27, 95%CI 1.78–2.89; *P* < 0.001; Fig. 3d). To analyze the clinicopathological correlation of the RiskScore, we obtained vascular invasion and tumor differentiation information from the *TCGA* dataset, and compare the difference between high- and low-risk groups. For vascular invasion status, although there was no significant difference, sample with vascular invasion has a higher proportion of high-risk patients (Supplementary Figure 3A). In addition, there are significant differences between high- and low-risk patients on tumor differentiation (Supplementary Figure 3B). G1 group has the highest proportion of low-risk patients, and G4 group has the highest proportion of high-risk patients, which suggests that the RiskScore is significantly correlated with tumor differentiation.

### Validation of the 11-gene signature in the internal dataset and two external HCC datasets

**Fig. 4:**
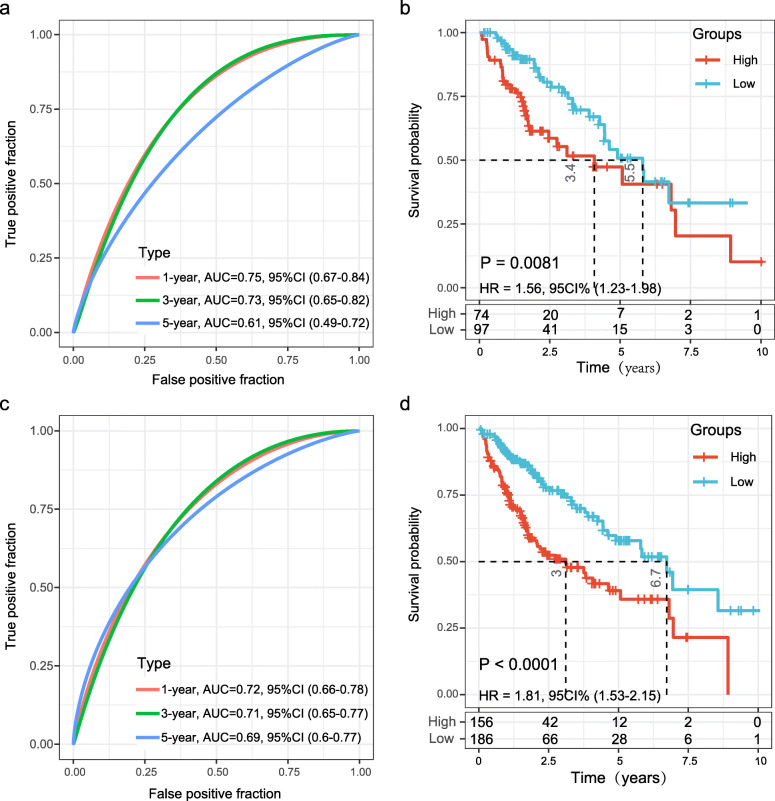
Internal validation of the 11-gene signature’s robustness in the *TCGA* validation cohort and the entire *TCGA* cohort. **a** ROC curve of the 11-gene signature for OS in the *TCGA* validation set; **b** Kaplan-Meier survival analysis of OS for patients in the *TCGA* validation set; **c** ROC curve of the 11-gene signature for OS in the entire *TCGA* set; **d** Kaplan-Meier survival analysis of OS for patients in the entire *TCGA* set

The *TCGA* testing dataset (*n* = 171) and the entire *TCGA* HCC dataset (*n* = 342) were used for internal validation, patients in both two sets were calculated using the same coefficients. Patients were sub-grouped using the same cut-off value as the training set. The corresponding ROC curve and Kaplan-Meier survival curves for the *TCGA* testing set and the entire *TCGA* dataset showed that the AUCs of the signature remained high and the high-risk groups had consistently shorter OS periods than the low-risk groups (Fig. 4).

**Fig. 5:**
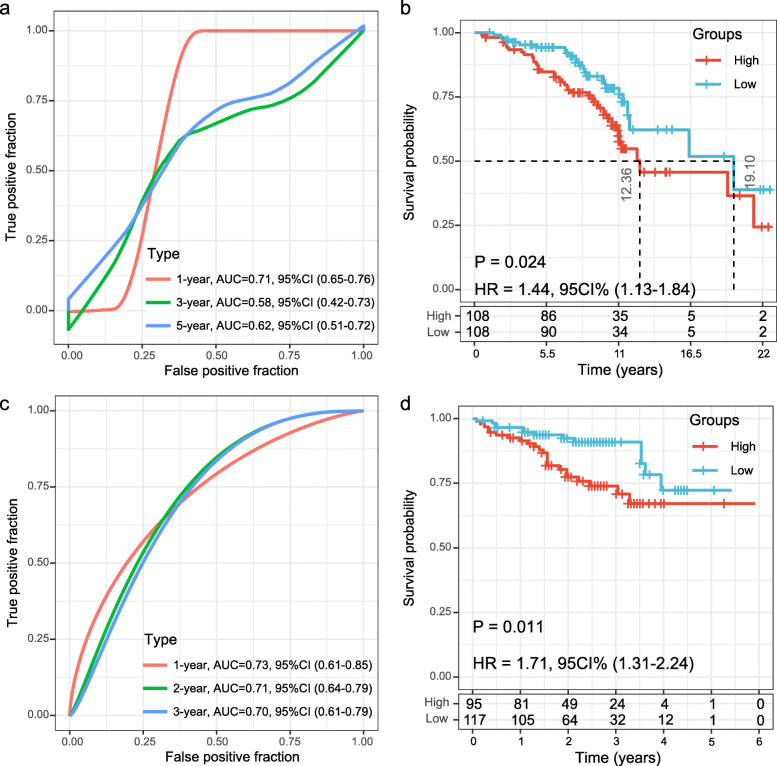
External validation of the 11-gene signature’s robustness in the GSE15654 and ICGC HCC cohorts. **a** ROC curve of the 11-gene signature for OS in the GSE15654 cohort; **b** Kaplan-Meier survival curve of OS for patients with HCC based on the 11-gene signature in the GSE15654 cohorts. **c** ROC curve of the 11-gene signature for OS in the ICGC HCC cohort; **d** Kaplan-Meier survival curve of OS based on the 11-gene signature in the ICGC HCC cohorts

Subsequently, the prognostication efficiency of our 11-gene signature was also calculated in the GSE15654 and ICGC HCC cohort. The results showed that the association between the gene expression and risk score was consistent with that in the training and internal validation sets (Fig. 5). In the GSE15654 dataset, The ROC curve revealed that the AUCs of the prognostic 11-gene signature for 1-year, 3-year and 5-year survival were 0.71, 0.58 and 0.62, respectively (Fig. 5a). As expected, patients in the GSE15654 dataset with high risk-scores had shorter OS than those with low risk scores (HR = 1.44, 95%CI 1.13–1.84; *P* = 0.024; Fig. 5b). In the ICGC HCC cohort, the ROC curve showed that the AUCs of the 11-gene signature for one-year, three-year and five-year survival were consistently greater than 0.70 (Fig. 5c). As expected, patients in the ICGC HCC cohort with high risk-scores had shorter OS than those with low risk scores (HR = 1.71, 95%CI 1.31–2.24; *P* = 0.011; Fig. 5d). Therefore, the 11-gene signature displayed solid effective prognostic classification performance in the two external validation sets.

### Univariate and multivariate cox regression analyses of the 11-gene signature

**Fig. 6:**
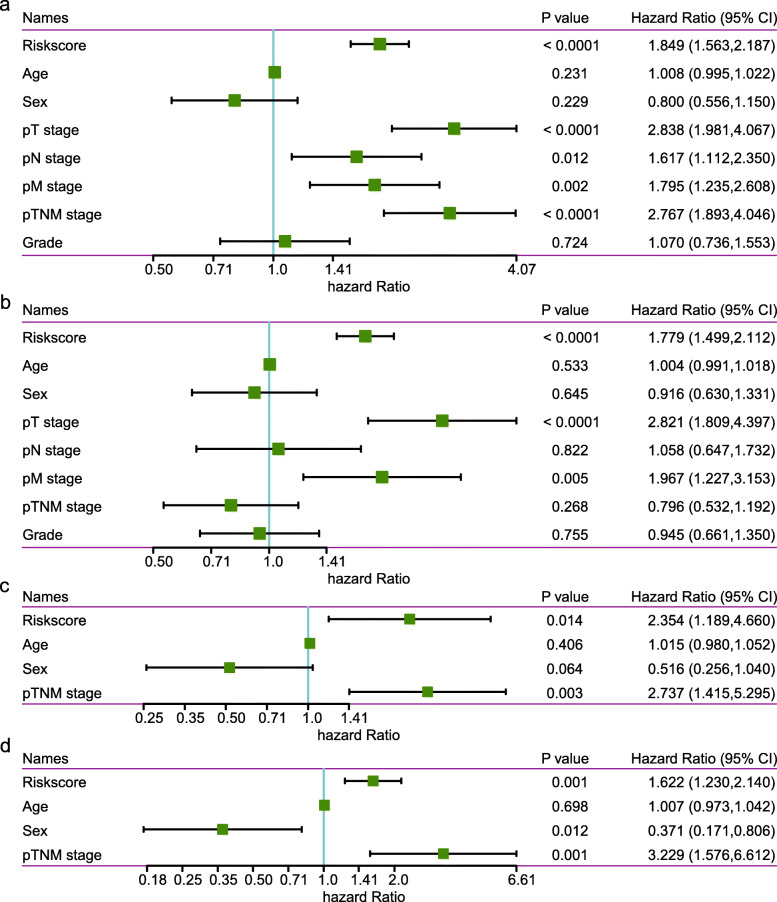
Cox regression analyses of prognostic variables in *TCGA* and ICGC HCC datasets. **a**-**b**) Forest plot of the univariate (**a**) and multivariate (**b**) Cox regression analyses in *TCGA* HCC dataset; **c**-**d** Forest plot of the univariate (**c**) and multivariate (**d**) Cox regression analyses in ICGC HCC dataset

To identify whether the 11-gene signature is an independent prognostic predictor in clinical application, we applied univariate and multivariate Cox regression analysis to systematically evaluate the prognosis of patients in the entire *TCGA* and ICGC HCC dataset. In the entire *TCGA* HCC dataset, univariate analysis of survival revealed that the 11-gene signature (*P* < 0.001), pT stage (*P* < 0.001), pN stage (*P* < 0.001), pM stage (*P* < 0.001) and pTNM stage (*P* < 0.001) were prognostic indicators of OS (Fig. 6a). However, multivariate Cox regression analysis showed that only 11-gene signature (*P* < 0.001) in addition to pT stage (*P* < 0.001) and pN stage (*P* = 0.005) were independent prognostic indicators of OS (Fig. 6b). In the ICGC HCC dataset, univariate and multivariate analysis of survival revealed that both the 11-gene signature and pTNM stage were independent prognostic indicators of OS (Fig. 6c-d). Overall, these results suggest that the 11-gene signature is a potential independent prognostic factor for HCC.

### Construction of a comprehensive nomogram for survival prediction of HCC patients

**Fig. 7:**
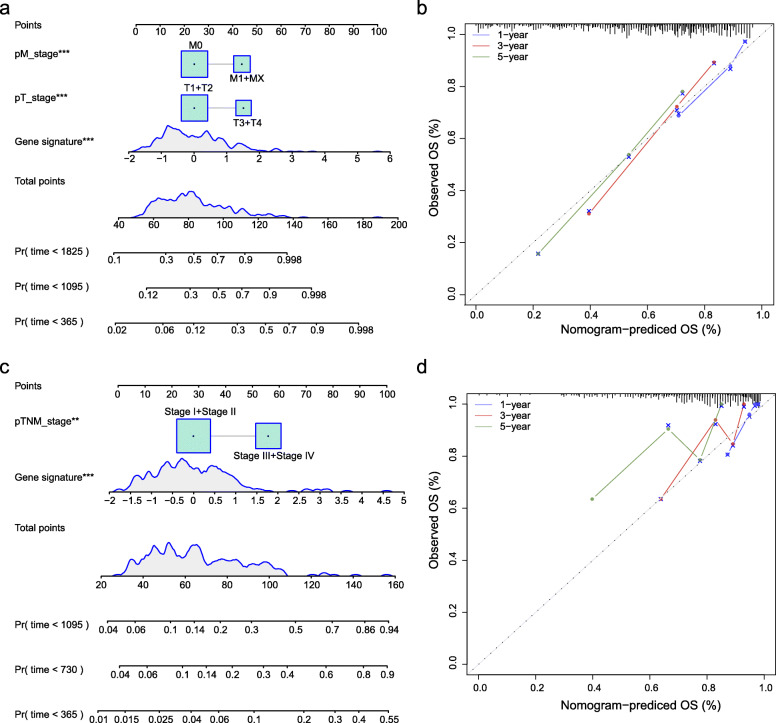
Nomograms for prediction of the outcome of patients with HCC. **a** A nomogram established by integrating the gene-signature with the clinicopathologic features in the TCGA HCC dataset. **b** Calibration curves of the developed nomogram for predicting OS in the TCGA HCC dataset. **c** A nomogram established by integrating the gene-signature with the clinicopathologic features in the ICGC HCC dataset. **d** Calibration curves of the developed nomogram for predicting OS in the ICGC HCC dataset

In order to maximize the utility of the signature, we further integrated risk score and other prognostic clinical factors identified by univariate Cox regression analysis (Fig. 6b) to construct a novel nomogram for the survival prediction of HCC patients (Fig. 7a). The one-year, three-year and five-year calibration curves of the nomogram verified the consistency between predicted and actual survival probability of HCC patients (Fig. 7b). And also, in the ICGC HCC dataset, the one-year, three-year and five-year calibration curves of the nomogram the nomogram showed a similar result (Fig. 7c-d).

### GSEA analysis of enriched pathway based on risk score

ssGSEA was performed to determine the potential related pathways according to patients’ prognostic risk in *TCGA*, ICGC and GSE15654 cohorts, and pathways with False Discovery Rates (FDR) < 0.05 were derived. By divided samples into high-risk group and low-risk group based on whether the Riskscore is greater than 0, and analyzed the enriched pathway in both groups by using GSEA, we found that a total of 20 pathways were identified in the *TCGA* HCC cohort (Supplementary Table S6), 15 pathways were identified in the ICGC HCC cohort (Supplementary Table S7), and 46 pathways were identified in the GSE15654 cohort (Supplementary Table S8). As top pathways showed in Supplementary Figure 4, all of them were significantly enriched in the low-risk group. Thus, the 11-gene signature may involve in the development and progression of HCC by participating these pathways.

## Discussion

Since the theory of cancer stem cells (CSCs) was proposed, CSCs have been well recognized and characterized in many human malignancies including hepatic cancer [3]. Cumulative evidences have been yielded in the contribution of CSCs on the development of cancer recurrence, metastasis, and chemo- and radio-resistance in hepatocellular carcinoma [9]. However, several physiopathology and mechanistic questions of hepatic CSC still need to be illuminated. In this study, we identified the molecular subtypes of HCC based on the expression of stemness-related genes (SRGs), which provided a new molecular subtype classification of HCC, and further studied the genomic background of the molecular characteristics of HCC. In addition, we constructed a PPI network based on the 987 differential expression genes obtained from DEseq analysis of the differential genes in each subtype; then constructed a 11-gene signature prognostic model based on the hub genes in the PPI network; after a three-phase training, test and validation process, we confirmed that the 11-gene signature is able to exert stable prediction performance in datasets from different platforms, which means that it has strong robustness on classification of the prognostic risk of patients with HCC.

The associations between SRG expression and immune-infiltration or clinical outcomes have been detected before but has not been explored in HCC [10–12]. In our analysis, based on the expression profile of prognostic SRGs, we demonstrated that the Cluster 2 subtype is associated with the highest infiltration of immune cells, and we identified that tumors with more active SRG expression have higher immune-infiltration in tumor microenvironment (TME) and significantly worse prognosis, which means that increased expression of stemness-related genes and increased immune infiltration may contribute to a poor prognosis. CSCs can show special characteristics to evade the recognition of innate and adaptive immunity, transform TME into immunosuppressive and promote tumorigenic landscape. In addition, immune cells sculpted by CSCs can affect the differentiation and phenotype of tumor cells in TME [13]. Therefore, tumors with more active SRG expression may have higher immune-infiltration in the tumor microenvironment and significantly worse prognosis. Thus, CSCs maintain the malignant phenotype of tumor cells and induce poor clinical outcomes by remodeling the immune contexture. Various stemness-related pathways such as Notch and Hedghog signaling are also widely involved in tumor immune regulation; in addition, CSCs can induce immune escape by activating their own oncogenic pathways, such as Wnt and Hippo signaling, etc. [14]. The expression activity of SRGs to some extent represent the immunomodulatory properties of CSCs; in addition, this immune-related difference in SRGs based molecular subtype may reflect the effects of stemness on TME and the reason why a part of HCC is so fatal regardless of aggressive therapy. Targeting SRGs will facilitate the development of current therapeutic modalities and the R&D of ground-breaking strategies. Our study suggests a great potential for the use of SRG profiling as a powerful marker in prognostication and inform treatment decisions for HCC patients.

We further established a 11-gene signature that could classify patients’ overall survival. Among the 11 biomarker genes (CDX2, PON1, ADH4, RBP2, LCAT, GAL, LPA, CYP19A1, GAST, SST and UGT1A8) discovered by the present study, caudal-related homeobox 2 (CDX2) is an intestinal-specific homeobox transcription factor that plays a crucial role in the development, proliferation, and differentiation of intestinal epithelium [15, 16]. CDX2 have emerged as important modulators of cancer aggressiveness and can influence the viability of HCC cells by transcription regulating oncogene CDH17 [17]. Moreover, CDX2 also implicated in the differentiation of human and mouse pluripotent stem cells [18]. Retinoblastoma-Binding Protein 2 (RBP2) is a histone demethylase over-expressed in HCC [19], and RBP2 can induce CSC phenotypes through converting renal cell carcinoma cells into a more mesenchymal phenotype [20]. The promoter gene polymorphism of CYP19A1 has been linked with the risk of hepatocellular carcinoma [21, 22]; in addition, it can promote the metastatic homing and proliferation of stem-like prostate cancer cells in the bone marrow [23]. The question that whether CDX2, RBP2, and CYP19A1 involve in the behavior regulation of CSCs subgroup in HCC should be further recognized. Gastrin (GAST) is a trophic factor within the normal gastrointestinal tract and its precursor forms can express in HCC [24]. Interestingly, GAST can also expresses in a group of primary human tumors, including neuronal, renal, and myogenic stem cell tumors [25], which suggest that GAST may play a previously unrecognized role in human CSC. Galanin and GMAP Prepropeptide (GAL) has been demonstrated to activate in human HCC, and it prefers to accumulate in the stromal tissue around the HCC cells [26]; Somatostatin (SST) is a kind of hormone that can inhibits the release of various secondary hormones; Paraoxonase 1 (PON1) was found to be a highly related predictor of recurrence and metastasis in HCC [27]; Production and Homeostasis of Lipoprotein(A) (LPA) can be impaired when liver cancer occurs [28]; and both the downregulation of PON1 [27] and alcohol dehydrogenase 4 (Class II), Pi polypeptide (ADH4) [29] can confers poor survival time of HCC. However, the relationship among these genes and CSC or stemness was seldom reported. Besides, seldom has been recognized on the expression or role of Lecithin-Cholesterol Acyltransferase (LACT) and UDP Glucuronosyltransferase Family 1 Member A8 (UGT1A8) in HCC or even CSCs. Nevertheless, although some of previous studies have identified some these genes as prognostic marker in HCC, whereas they were limited by just single gene detected, small cohort, and deficiency on independent validation. The use of the LASSO Cox regression model [30] and nomogram [31] allowed us to integrate multiple genes into one module, which has significantly higher prognostication performance than that of single gene alone or even some previous reported gene signatures.

Some limitations of this study should be taken into consideration. Firstly, since the *TCGA*, ICGC and GSE15654 datasets we enrolled only included Caucasian population, most of which are hepatitis C-related HCC, this present study may not include patients with hepatitis B-related HCC from other areas loading distinct genetic phenotypes and clinical characteristics, making it vulnerable to the intrinsic biases of such a research format. Obviously, our results should be training in hepatitis B-related HCC cohorts, and further validated by prospective study in some worldwide multicenter clinical study. Moreover, its area under ROC curve should be optimized to improve the prognostic accuracy, and also its prediction value in early HCC must be further evaluated. In addition, despite growing studies began focus on the interaction of tumor cells and associated stemness in human malignancies, most SRG are not yet functionally annotated in HCC, and the biofunction of our 11 genes have not yet been fully investigated in previous studies. Although the physiopathological roles of the gene signature were annotated using computational approaches, additional studies should be carried out to further disclose the mechanisms of these 11 genes involved in the carcinogenesis of HCC. Further, more evidences are required to find out the biological foundation of their dysregulation in HCC.

## Conclusions

In summary, for the first time, we profiled the stemness related molecular subtype in HCC and our study may provide an assessment approach for the CSC-based classification of HCC. Moreover, we identified a stemness-related gene signature which could classify the prognostic risk of patients with HCC. This method might, therefore, help with patient management and individualized therapy of patients with HCC.

## Supplementary Information

**Additional file 1: Supplementary Figure 1.** Distribution of clinicopathological parameters in the four subtypes. **Supplementary Figure 2.** (A-D) Volcano map of differentially expressed genes (DEG) between each subtype and the other subtypes; (E-F) The Venn diagrams of the overlapping genes among the DEGs in each molecular subtype. **Supplementary Figure 3.** Distribution of clinicopathological parameters including vascular invasion (A) and tumor differentiation (B) between the high-risk and low-risk groups. **Supplementary Figure 4.** ssGSEA result according to the risk-score of HCC samples in each dataset, enrichment pathways that were significantly correlated in the low-risk groups (FDR < 0.05). **Supplementary Table 1.** Pathways related to cancer stem cells in Reactome and GO databases. **Supplementary Table S2.** List of SRGs correlated with the overall survival of patients with HCC. **Supplementary Table S3.** Protein-protein interaction pairs with a score higher than 0.7. **Supplementary Figure S4.** Hub genes identified by the Degree, Closeness and Betweenness. **Supplementary Table S4.** Betweenness in the PPI network. **Supplementary Figure S5.** Genes with high degree, closeness, and betweenness scores. **Supplementary Table S6.** Pathways identified in the TCGA HCC cohort. **Supplementary Table S7.** Pathways identified in the ICGC HCC cohort. **Supplementary Table S8.** Pathways identified in the GSE15654 cohort.

## Data Availability

The datasets generated and analyzed during the current study are available in the *TCGA* repository (https://portal.gdc.cancer.gov/), ICGC database (https://icgcportal.genomics.cn/) and the GEO repository (https://www.ncbi.nlm.nih.gov/geo/). The datasets generated and analyzed during the current study are available in the TCGA repository (https://portal.gdc.cancer.gov/) with the accession number TCGA-LIHC; the ICGC Data Portal (https://icgcportal.genomics.cn/) with the accession number HCC; and the GEO repository (https://www.ncbi.nlm.nih.gov/geo/) the accession number was GSE15654. Public access to these databases is open. Coherently expressed signatures of human metabolism-related pathways were all download from the Reactome pathway database (https://reactome.org/) and derived by aggregating MSigDB version 7.0 gene sets.

## Abbreviations

ADH4: Alcohol dehydrogenase 4 (Class II), Pi polypeptide
CDF: Cumulative distribution function
CDX2: Caudal-related homeobox 2
CSC: Cancer stem cell
DCA: Decision curve analysis
DEG: Differentially expressed gene
DFS: Disease specific survival
EMRG: Energy metabolism-related genes
FDR: False discovery rate
FPKM: Fragments Per Kilobase of exon model per Million mapped fragments
GAL: Galanin and GMAP Prepropeptide
GAST: Gastrin
GEO: Gene Expression Omnibus
HCC: Hepatocellular carcinoma
ICGC: International Cancer Genome Consortium
KEGG: Kyoto Encyclopedia of Genes and Genomes
LACT: Lecithin-Cholesterol Acyltransferase
LASSO: Least absolute shrinkage and selection operator
MSigDB: Molecular Signature Database
OS: Overall survival
PAC: Ambiguously clustered pairs
PCA: Principal component analysis
PON1: Paraoxonase 1
PPI: Protein-protein interaction
RBP2: Retinoblastoma-Binding Protein 2
ROC: Receiver operating characteristic
SRG: Stemness related gene
ssGSEA: Single-sample Gene set enrichment analysis
STRING: Search Tool for the Retrieval of Interacting Genes
*TCGA*: The Cancer Genome Atlas
TME: Tumor microenvironment
UGT1A8: UDP Glucuronosyltransferase Family 1 Member A8
