# Supplementary material for: A stemness-based eleven-gene signature correlates with the clinical outcome of hepatocellular carcinoma
Source: BMC Cancer. 2021 Jun 19;21:716. doi: 10.1186/s12885-021-08351-0 (PMC8214273; doi:10.1186/s12885-021-08351-0)
Supplement: Supplementary file 1 — Additional file 1: Supplementary Figure 1. Distribution of clinicopathological parameters in the four subtypes. Supplementary Figure 2. (A-D) Volcano map of differentially expressed genes (DEG) between each subtype and the other subtypes; (E-F) The Venn diagrams of the overlapping genes among the DEGs in each molecular subtype. Supplementary Figure 3. Distribution of clinicopathological parameters including vascular invasion (A) and tumor differentiation (B) between the high-risk and low-risk groups. Supplementary Figure 4. ssGSEA result according to the risk-score of HCC samples in each dataset, enrichment pathways that were significantly correlated in the low-risk groups (FDR < 0.05). Supplementary Table 1. Pathways related to cancer stem cells in Reactome and GO databases. Supplementary Table S2. List of SRGs correlated with the overall survival of patients with HCC. Supplementary Table S3. Protein-protein interaction pairs with a score higher than 0.7. Supplementary Figure S4. Hub genes identified by the Degree, Closeness and Betweenness. Supplementary Table S4. Betweenness in the PPI network. Supplementary Figure S5. Genes with high degree, closeness, and betweenness scores. Supplementary Table S6. Pathways identified in the TCGA HCC cohort. Supplementary Table S7. Pathways identified in the ICGC HCC cohort. Supplementary Table S8. Pathways identified in the GSE15654 cohort. [file 12885_2021_8351_MOESM1_ESM.pdf]

A stemness-based eleven-gene signature correlates with the clinical outcome of hepatocellular carcinoma

Running title: stemness-based prognostic gene signature in hepatocellular carcinoma

Liang Hong[1], Yu Zhou[1], Xiangbang Xie[2], Wanrui Wu[2], Changsheng Shi[2], Heping Lin[3], Zhenjing Shi[2]

[1] Department of Infectious, the Third Affiliated Hospital of Wenzhou Medical University, Ruian, Zhejiang 325200, P.R. China

[2] Department of Interventional, the Third Affiliated Hospital of Wenzhou Medical University, Ruian, Zhejiang 325200, P.R. China.

[3] Department of Respiratory, the Third Affiliated Hospital of Wenzhou Medical University, Ruian, Zhejiang 325200, P.R. China.

Corresponding author：

Zhenjing Shi

Department of Interventional, the Third Affiliated Hospital of Wenzhou Medical University, Wenzhou, Zhejiang 325699, P.R. China

Email: szj258@126.com

&

Heping Lin

Department of Respiratory, the Third Affiliated Hospital of Wenzhou Medical University, Wenzhou, Zhejiang 325699, P.R. China

Email: 13857793035@163.com

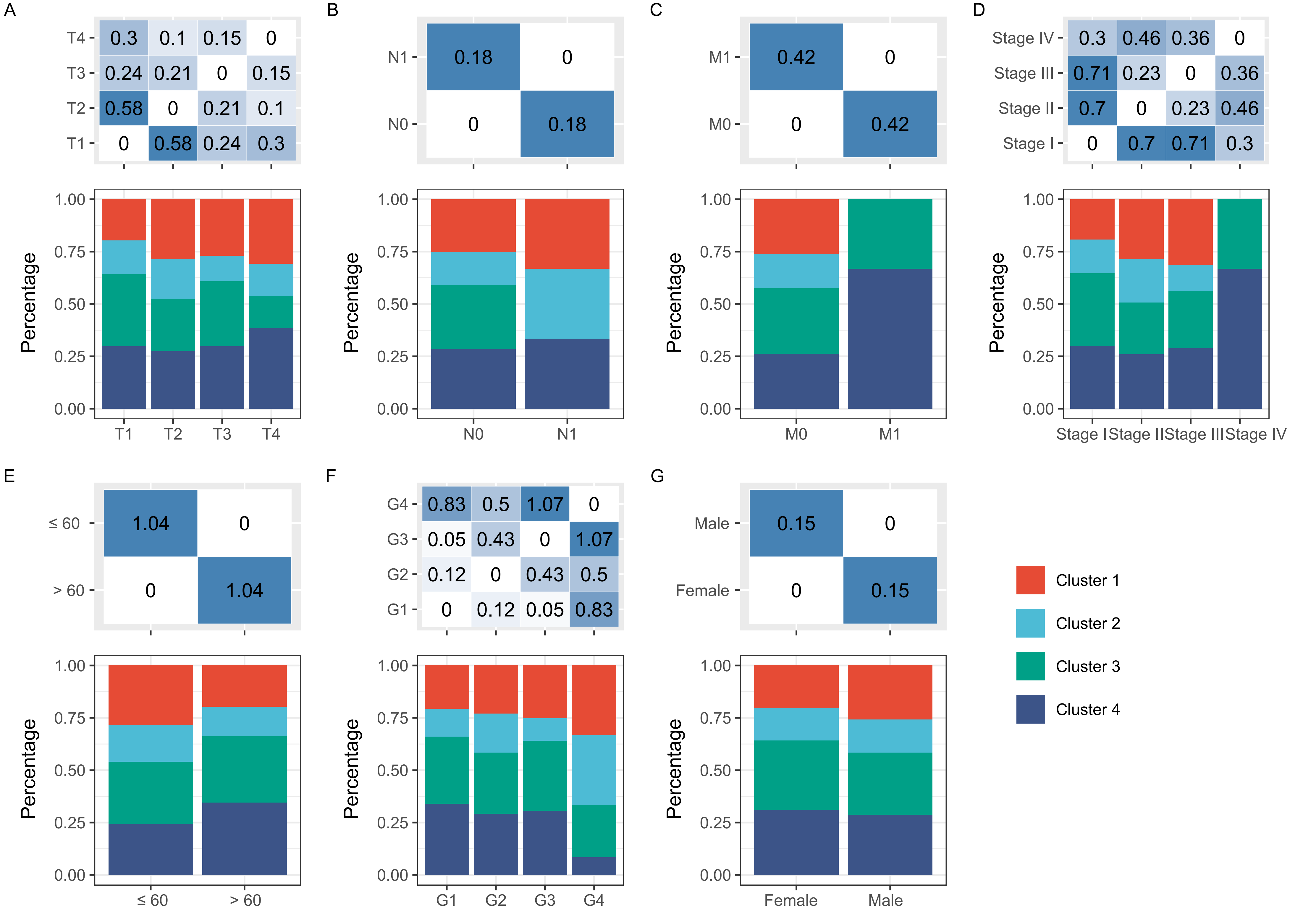

A
B
C
D
E
F
G
T4
T3
T2
T1
0.3
0.1
0.15
0
0.24
0.21
0
0.15
0.58
0
0.21
0.1
0
0.58
0.24
0.3
N1
N0
0.18
0
0
0.18
M1
M0
0.42
0
0
0.42
Stage IV
Stage III
Stage II
Stage I
0.3
0.46
0.36
0
0.71
0.23
0
0.36
0.7
0
0.23
0.46
0
0.7
0.71
0.3
Percentage
1.00
0.75
0.50
0.25
0.00
T1
T2
T3
T4
N0
N1
M0
M1
Stage I
Stage II
Stage III
Stage IV
≤ 60
> 60
1.04
0
0
1.04
G4
G3
G2
G1
0.83
0.5
1.07
0
0.05
0.43
0
1.07
0.12
0
0.43
0.5
0
0.12
0.05
0.83
Male
Female
0.15
0
0
0.15
G1
G2
G3
G4
Female
Male
Cluster 1
Cluster 2
Cluster 3
Cluster 4

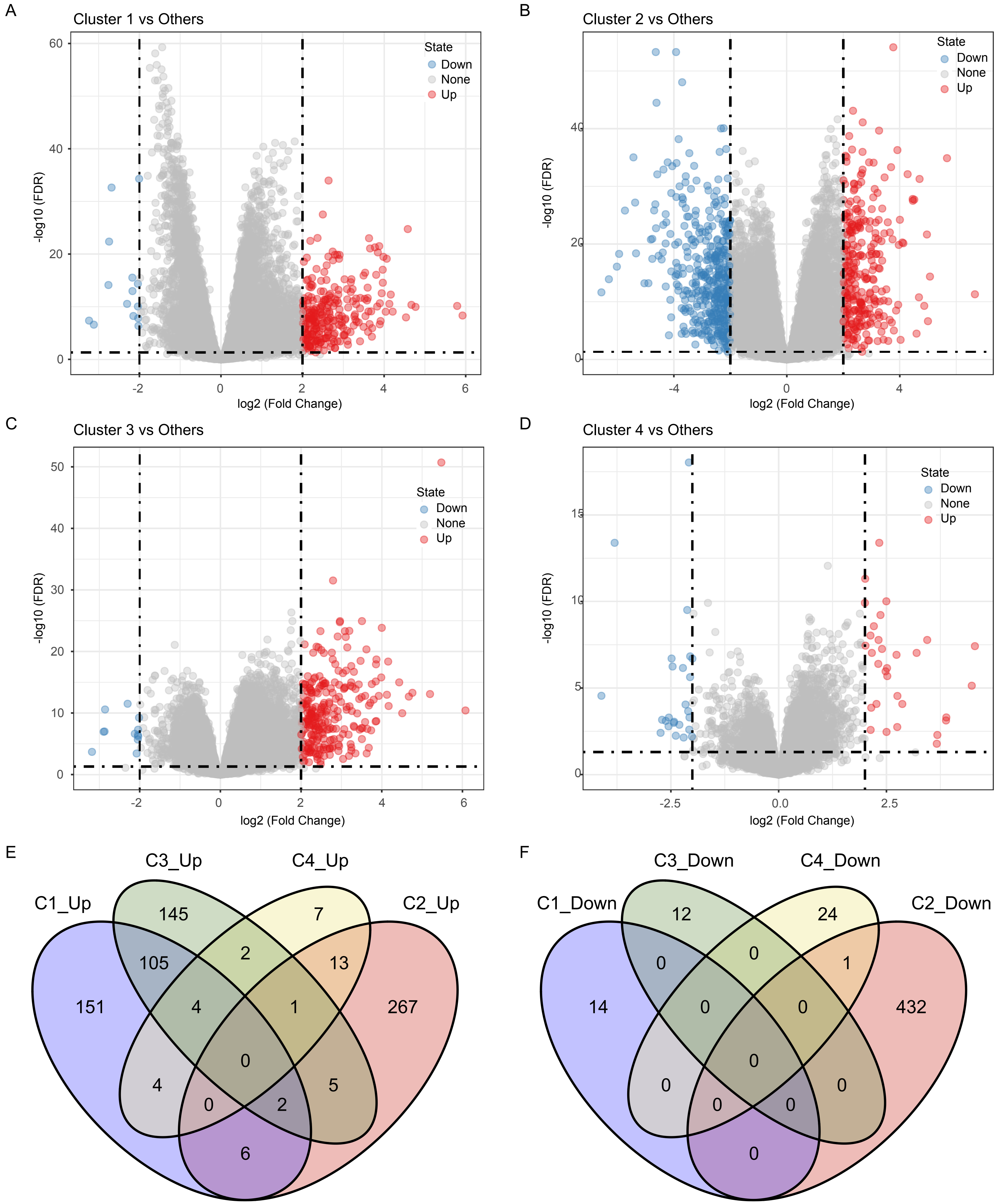

A
Cluster 1 vs Others
State
Down
None
Up
-log10 (FDR)
log2 (Fold Change)
B
Cluster 2 vs Others
C
Cluster 3 vs Others
D
Cluster 4 vs Others
E
C1_Up
C3_Up
C4_Up
C2_Up
145
7
105
2
13
151
4
1
267
0
4
0
2
5
6
F
C1_Down
C3_Down
C4_Down
C2_Down
12
24
0
0
1
14
0
0
432
0
0
0
0
0
0

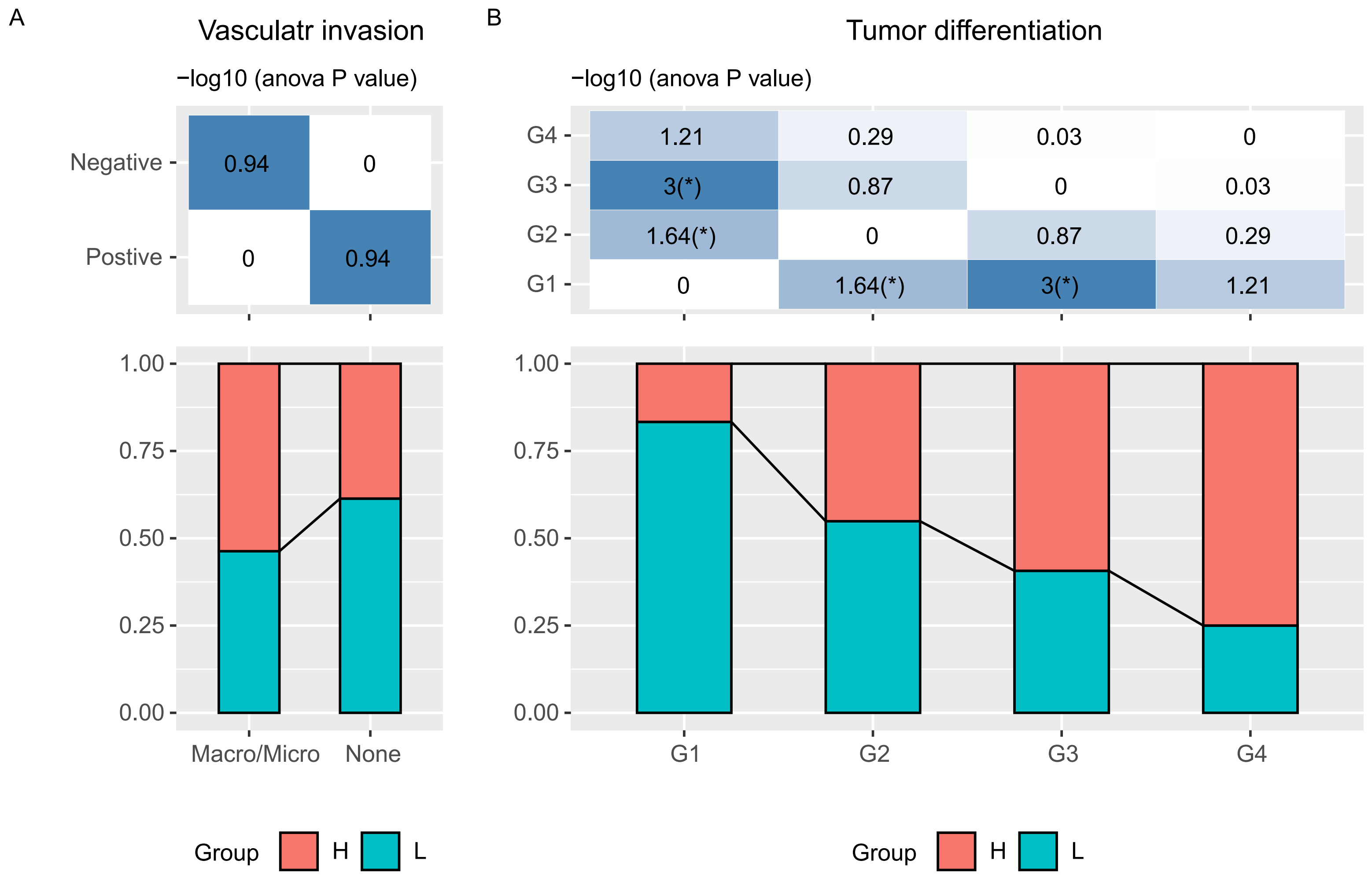
A
Vasculatr invasion
−log10 (anova P value)
Negative
Postive
0.94
0
0
0.94
1.00
0.75
0.50
0.25
0.00
Macro/Micro
None
Group
H
L
B
Tumor differentiation
−log10 (anova P value)
G4
G3
G2
G1
1.21
0.29
0.03
0
3(*)
0.87
0
0.03
1.64(*)
0
0.87
0.29
0
1.64(*)
3(*)
1.21
1.00
0.75
0.50
0.25
0.00
G1
G2
G3
G4
Group
H
L

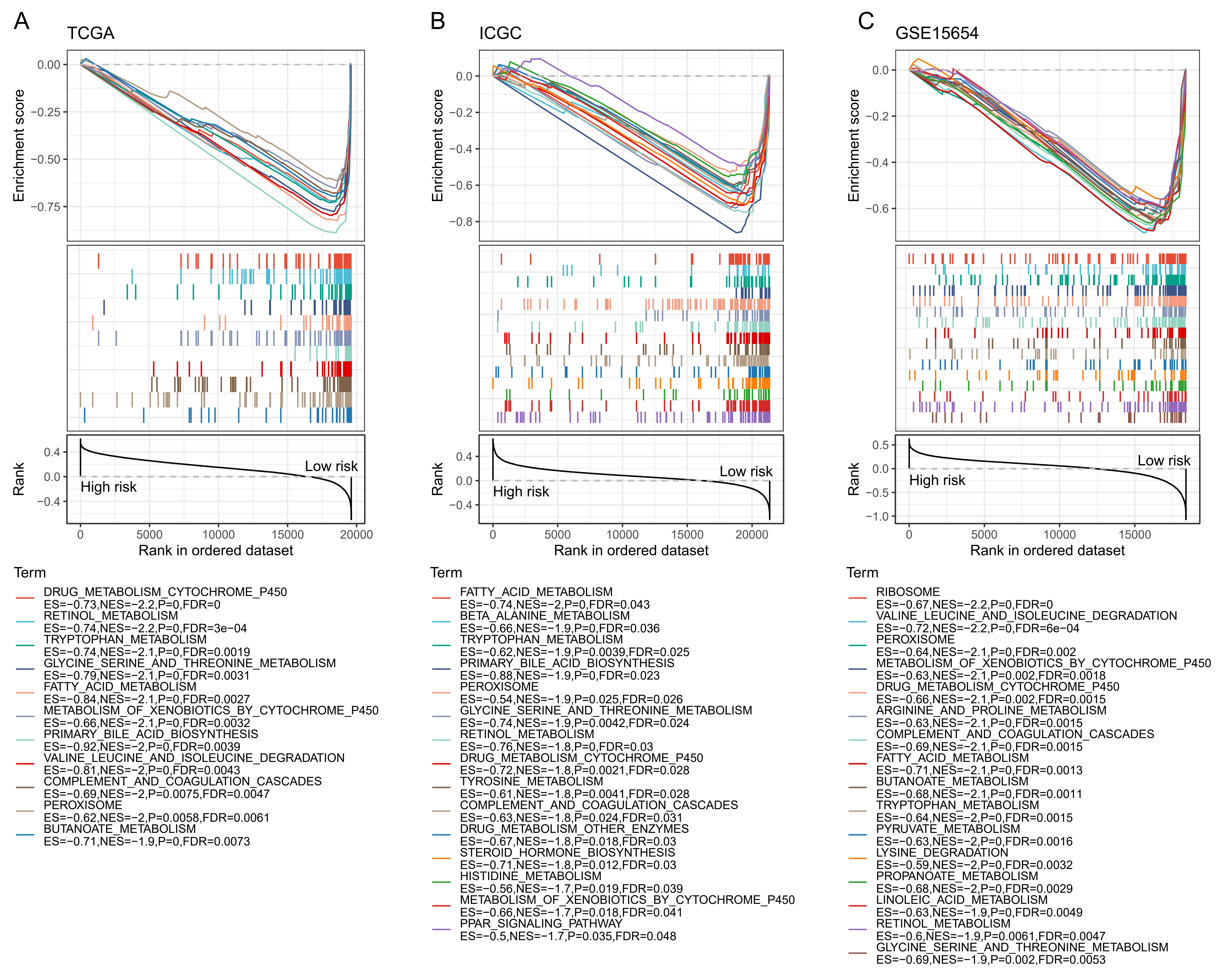
A
TCGA
B
ICGC
C
GSE15654
Enrichment score
Rank
High risk
Low risk
Rank in ordered dataset
Term
DRUG_METABOLISM_CYTOCHROME_P450
ES=−0.73,NES=−2.2,P=0,FDR=0
RETINOL_METABOLISM
ES=−0.74,NES=−2.2,P=0,FDR=3e−04
TRYPTOPHAN_METABOLISM
ES=−0.74,NES=−2.1,P=0,FDR=0.0019
GLYCINE_SERINE_AND_THREONINE_METABOLISM
ES=−0.79,NES=−2.1,P=0,FDR=0.0031
FATTY_ACID_METABOLISM
ES=−0.84,NES=−2.1,P=0,FDR=0.0027
METABOLISM_OF_XENOBIOTICS_BY_CYTOCHROME_P450
ES=−0.66,NES=−2.1,P=0,FDR=0.0032
PRIMARY_BILE_ACID_BIOSYNTHESIS
ES=−0.92,NES=−2,P=0,FDR=0.0039
VALINE_LEUCINE_AND_ISOLEUCINE_DEGRADATION
ES=−0.81,NES=−2,P=0,FDR=0.0043
COMPLEMENT_AND_COAGULATION_CASCADES
ES=−0.69,NES=−2,P=0.0075,FDR=0.0047
PEROXISOME
ES=−0.62,NES=−2,P=0.0058,FDR=0.0061
BUTANOATE_METABOLISM
ES=−0.71,NES=−1.9,P=0,FDR=0.0073
Term
FATTY_ACID_METABOLISM
ES=−0.74,NES=−2,P=0,FDR=0.043
BETA_ALANINE_METABOLISM
ES=−0.66,NES=−1.9,P=0,FDR=0.036
TRYPTOPHAN_METABOLISM
ES=−0.62,NES=−1.9,P=0.0039,FDR=0.025
PRIMARY_BILE_ACID_BIOSYNTHESIS
ES=−0.88,NES=−1.9,P=0,FDR=0.023
PEROXISOME
ES=−0.54,NES=−1.9,P=0.025,FDR=0.026
GLYCINE_SERINE_AND_THREONINE_METABOLISM
ES=−0.74,NES=−1.9,P=0.0042,FDR=0.024
RETINOL_METABOLISM
ES=−0.76,NES=−1.8,P=0,FDR=0.03
DRUG_METABOLISM_CYTOCHROME_P450
ES=−0.72,NES=−1.8,P=0.0021,FDR=0.028
TYROSINE_METABOLISM
ES=−0.61,NES=−1.8,P=0.0041,FDR=0.028
COMPLEMENT_AND_COAGULATION_CASCADES
ES=−0.63,NES=−1.8,P=0.024,FDR=0.031
DRUG_METABOLISM_OTHER_ENZYMES
ES=−0.67,NES=−1.8,P=0.018,FDR=0.03
STEROID_HORMONE_BIOSYNTHESIS
ES=−0.71,NES=−1.8,P=0.012,FDR=0.03
HISTIDINE_METABOLISM
ES=−0.56,NES=−1.7,P=0.019,FDR=0.039
METABOLISM_OF_XENOBIOTICS_BY_CYTOCHROME_P450
ES=−0.66,NES=−1.7,P=0.018,FDR=0.041
PPAR_SIGNALING_PATHWAY
ES=−0.5,NES=−1.7,P=0.035,FDR=0.048
Term
RIBOSOME
ES=−0.67,NES=−2.2,P=0,FDR=0
VALINE_LEUCINE_AND_ISOLEUCINE_DEGRADATION
ES=−0.72,NES=−2.2,P=0,FDR=6e−04
PEROXISOME
ES=−0.64,NES=−2.1,P=0,FDR=0.002
METABOLISM_OF_XENOBIOTICS_BY_CYTOCHROME_P450
ES=−0.63,NES=−2.1,P=0.002,FDR=0.0018
DRUG_METABOLISM_CYTOCHROME_P450
ES=−0.66,NES=−2.1,P=0.002,FDR=0.0015
ARGININE_AND_PROLINE_METABOLISM
ES=−0.63,NES=−2.1,P=0,FDR=0.0015
COMPLEMENT_AND_COAGULATION_CASCADES
ES=−0.69,NES=−2.1,P=0,FDR=0.0015
FATTY_ACID_METABOLISM
ES=−0.71,NES=−2.1,P=0,FDR=0.0013
BUTANOATE_METABOLISM
ES=−0.68,NES=−2.1,P=0,FDR=0.0011
TRYPTOPHAN_METABOLISM
ES=−0.64,NES=−2,P=0,FDR=0.0015
PYRUVATE_METABOLISM
ES=−0.63,NES=−2,P=0,FDR=0.0016
LYSINE_DEGRADATION
ES=−0.59,NES=−2,P=0,FDR=0.0032
PROPANOATE_METABOLISM
ES=−0.68,NES=−2,P=0,FDR=0.0029
LINOLEIC_ACID_METABOLISM
ES=−0.63,NES=−1.9,P=0,FDR=0.0049
RETINOL_METABOLISM
ES=−0.6,NES=−1.9,P=0.0061,FDR=0.0047
GLYCINE_SERINE_AND_THREONINE_METABOLISM
ES=−0.69,NES=−1.9,P=0.002,FDR=0.0053

Table1. Pathways related to cancer stem cells in Reactome and GO databases

Stem cell function related pathways	PathwayID	Gene Count
GO: Somatic Stem Cell Population Maintenance	GO: 0035019	72
GO: Negative Regulation of Stem Cell Differentiation	GO: 2000737	20
GO: Stem Cell Proliferation	GO: 0072089	118
GO: Hematopoietic Stem Cell Differentiation	GO: 0060218	79
GO: Negative Regulation of Stem Cell Proliferation	GO: 2000647	16
GO: Stem Cell Division	GO: 0017145	41
GO: Hematopoietic Stem Cell Proliferation	GO: 0071425	23
GO: Positive Regulation of Stem Cell Differentiation	GO: 2000738	20
GO: Regulation of Stem Cell Population Maintenance	GO: 2000036	28
GO: Neuronal Stem Cell Population Maintenance	GO: 0097150	22
GO: Regulation of Stem Cell Proliferation	GO: 0072091	67
GO: Somatic Stem Cell Division	GO: 0048103	24
GO: Stem Cell Differentiation	GO: 0048863	248
GO: Positive Regulation of Stem Cell Proliferation	GO: 2000648	40
GO: Regulation of Stem Cell Differentiation	GO: 2000736	112
GO: Hematopoietic Stem Cell Migration	GO: 0035701	6
GO: Stem Cell Fate Commitment	GO: 0048865	9
GO: Mesenchymal Stem Cell Maintenance Involved In Nephron Morphogenesis	GO: 0072038	6
GO: Mesenchymal Stem Cell Differentiation	GO: 0072497	8
GO: Mesenchymal Stem Cell Proliferation	GO: 0097168	5
GO: Asymmetric Stem Cell Division	GO: 0098722	10
GO: egulation of Hematopoietic Stem Cell Proliferation	GO: 1902033	9
GO: ositive Regulation of Hematopoietic Stem Cell Proliferation	GO: 1902035	5
GO: egative Regulation of Stem Cell Population Maintenance	GO: 1902455	8
GO: ositive Regulation of Stem Cell Population Maintenance	GO: 1902459	8
GO: egulation of Somatic Stem Cell Population Maintenance	GO: 1904672	7
GO: Negative Regulation of Somatic Stem Cell Population Maintenance	GO: 1904673	5
GO: Regulation of Stem Cell Division	GO: 2000035	10
GO: Regulation of Mesenchymal Stem Cell Differentiation	GO: 2000739	6
Reactome Transcriptional Regulation of Pluripotent Stem Cells	R-HSA-452723	31
**Total**	**1063 (unique 456)**	

Supplementary Table S2. List of SRGs correlated with the overall survival of patients with HC

Gene	P value	Hazard ratio	Low 95%CI	High 95%CI
CDX2	0.0043427	1.102912789	1.03111523	1.17970969
ZHX2	0.00036768	1.01797705	1.00804641	1.02800552
BCL9L	0.00581139	1.028071653	1.00804458	1.0484966
HES1	0.04245896	1.004434648	1.00015055	1.00873709
RBPJ	0.00016741	1.056139678	1.02652226	1.08661162
SMAD2	6.82E-06	1.201959518	1.10939909	1.30224254
SMAD4	0.00851427	1.046515721	1.01166321	1.08256892
ASCL2	0.00500467	1.051609731	1.0152976	1.08922057
PAX2	0.01017432	1.086989417	1.01999278	1.15838662
POLR2A	0.00067569	1.015927481	1.00671321	1.02522609
POLR2B	0.00020491	1.021867218	1.01026499	1.03360269
POLR2D	7.86E-07	1.066233852	1.03943985	1.09371854
POLR2E	0.0006421	1.009302974	1.00395036	1.01468412
POLR2G	9.29E-07	1.01499858	1.00897994	1.02105312
POLR2H	6.50E-05	1.015902639	1.00806715	1.02379904
POLR2K	0.01168176	1.004840161	1.00107605	1.00861843
POLR2L	0.02440832	1.000947433	1.00012231	1.00177323
SALL4	2.73E-05	1.0834159	1.04361107	1.12473894
VANGL2	0.02062568	1.027754634	1.00420503	1.0518565
RAF1	0.00773258	1.013945195	1.00366484	1.02433085
BCL9	0.00045932	1.025098528	1.0109807	1.0394135
PRDM16	0.00746308	1.20244424	1.05053689	1.37631735
SOX4	2.43E-05	1.009984862	1.00533679	1.01465442
SOX9	0.00900906	1.00462145	1.00115128	1.00810365
SPI1	0.00556867	1.007929612	1.00231675	1.01357391
BRAF	0.00148113	1.117259699	1.04342064	1.19632408
VPS72	2.71E-05	1.012377003	1.00657739	1.01821003
CUL4A	2.22E-05	1.023667935	1.01266346	1.03479199
ZSCAN10	0.00046872	279.3376021	11.9000462	6557.07505
LDB1	0.00663949	1.015117409	1.00417899	1.02617497
TRIM6	0.00013039	1.141468035	1.06665604	1.22152712
NELFB	7.14E-05	1.01329682	1.00671277	1.01992394
HNRNPU	1.41E-05	1.008733197	1.00478163	1.0127003
HES5	0.0077869	0.419438099	0.22118787	0.79537961
NOTCH1	0.00244775	1.029629357	1.01036314	1.04926296
CDK12	0.0392068	1.037056901	1.00180235	1.0735521
REST	0.00026861	1.075467248	1.03419282	1.11838894
CDK13	0.01015993	1.038523925	1.00901681	1.06889393
ARIH2	1.62E-05	1.054113092	1.02915787	1.07967344
FRS2	0.001803	1.052192226	1.01910538	1.08635329
RAB10	3.09E-07	1.007051181	1.00434501	1.00976464
TRIM71	0.01130421	1.031140125	1.00696286	1.05589789
CTNNA1	0.00123519	1.007179221	1.00281759	1.01155982
CTNNB1	0.01721118	1.003668837	1.00064926	1.00669752

CX3CR1	0.01696601	1.037627572	1.0066356	1.06957372
AGO3	0.00019989	1.414315702	1.17817436	1.69778682
ETV6	0.00214345	1.047975267	1.01708381	1.07980498
MECOM	1.16E-05	1.07957581	1.04324875	1.11716782
FBLN1	0.00061653	1.004455183	1.0019027	1.00701417
DAGLB	0.00023782	1.078400146	1.0358522	1.12269577
FGFR1	0.02871561	1.018235486	1.0018805	1.03485746
KDM1A	1.26E-08	1.027431103	1.0179003	1.03705115
ACSL6	0.03433796	0.939400546	0.88655275	0.99539862
PLXNB2	0.01340601	1.002421308	1.00050167	1.00434463
ASPM	7.91E-05	1.03816364	1.01903475	1.05765161
BABAM1	0.01064282	1.010144588	1.00235105	1.01799872
RACGAP1	2.62E-08	1.033339439	1.02147231	1.04534444
HIF1A	0.00058742	1.004297388	1.00184478	1.006756
HMGB2	1.55E-05	1.006799644	1.00371029	1.00989851
KITLG	0.00021853	1.063502062	1.02934595	1.09879155
NF2	0.00390311	1.036779844	1.01165622	1.06252739
PAFAH1B1	0.02494176	1.021050981	1.00262656	1.03981397
PAX6	0.03528542	1.30794847	1.01867739	1.67936308
PDCD2	7.81E-07	1.065991805	1.03930394	1.09336498
ABCB1	0.01250856	1.010361906	1.00222106	1.01856888
NDE1	3.61E-06	1.150650373	1.08432839	1.22102888
EIF2AK2	0.02025395	1.030757238	1.00473012	1.05745858
ZNF335	0.00015288	1.09908457	1.04662659	1.1541718
SMARCD3	0.01080744	1.021477646	1.00492182	1.03830622
SOX11	3.16E-06	2.038054747	1.51063838	2.7496105
ZFP36L1	0.00196932	1.003931834	1.00143999	1.00642987
TEAD3	0.02889519	1.015303725	1.0015649	1.02923102
TGFB1	0.00062883	1.004254031	1.00181308	1.00670093
THPO	0.03941757	1.016638204	1.00080013	1.03272693
VEGFA	7.45E-05	1.011582594	1.00583539	1.01736263
DAGLA	0.00049142	1.08989529	1.03839346	1.14395148
NKAP	0.00235725	1.066173189	1.02304073	1.11112416
FZD3	9.63E-06	1.546806986	1.27507151	1.87645307
HMGA2	0.00243906	1.062965313	1.02180888	1.10577944
DOCK7	5.10E-05	1.080987273	1.04101387	1.12249559
ARTN	0.04002732	1.177518457	1.00746845	1.3762711
ARHGEF2	4.77E-06	1.034566166	1.01961243	1.04973921
NUMBL	0.00678775	1.047391948	1.01286106	1.08310008
SART3	0.00014936	1.050284511	1.02398701	1.07725737
PSME3	5.87E-06	1.016889091	1.00954871	1.02428285
PSMD14	1.27E-09	1.037402963	1.0251775	1.04977422
BATF	0.00022434	1.008827297	1.00412885	1.01354773
ERCC2	0.04462823	1.016547907	1.00039383	1.03296283
PSME4	7.42E-05	1.044813356	1.02240121	1.0677168
ABL1	0.00081303	1.02216979	1.00913358	1.03537441

GATA3	0.00776797	1.047661421	1.01235475	1.08419944
KMT2A	0.00238863	1.092709208	1.03194381	1.15705274
MYB	5.44E-05	1.533360414	1.2459065	1.88713532
YTHDF2	1.27E-08	1.037157871	1.0242062	1.05027332
PUS7	2.48E-05	1.066976032	1.03530134	1.0996198
METTL3	6.45E-06	1.082154448	1.04566192	1.11992052
PSMA1	1.21E-08	1.026342222	1.0172053	1.03556121
PSMA3	1.44E-05	1.015250349	1.0083301	1.02221809
PSMA4	0.0049126	1.015924774	1.00480146	1.02717123
PSMA5	4.97E-06	1.014199603	1.00807971	1.02035665
PSMA6	0.04968829	1.042500333	1.0000567	1.08674532
PSMA7	0.00062974	1.002080927	1.00088731	1.00327597
PSMB2	1.79E-07	1.012731875	1.00793246	1.01755414
PSMB4	0.04392416	1.00054923	1.00001495	1.0010838
PSMB5	0.00014112	1.005442256	1.00263613	1.00825623
PSMC1	0.00535742	1.107552048	1.0307169	1.1901149
PSMC2	0.02838301	1.006928542	1.00073103	1.01316444
PSMC3	0.00337469	1.003323564	1.00110015	1.00555191
PSMC4	0.00393773	1.003849923	1.0012311	1.0064756
PSMC5	0.00048585	1.006419254	1.00280757	1.01004395
PSMC6	0.00125793	1.042519823	1.01647134	1.06923584
PSMD1	4.22E-09	1.022838635	1.01516229	1.03057303
PSMD2	6.26E-06	1.007054677	1.00398783	1.01013089
PSMD3	0.04147301	1.005191662	1.00020018	1.01020806
PSMD4	0.03150588	1.000859301	1.00007615	1.00164307
PSMD5	0.00350792	1.031984285	1.01039992	1.05402974
PSMD7	5.72E-05	1.017198271	1.00878466	1.02568205
PSMD8	0.02656375	1.003967887	1.0004606	1.00748747
PSMD9	0.00015874	1.114382747	1.05348202	1.17880408
PSMD10	6.58E-05	1.015060269	1.00763606	1.02253918
PSMD11	2.00E-05	1.021614283	1.01162484	1.03170237
PSMD12	5.06E-05	1.028713787	1.0147255	1.0428949
PSMD13	1.80E-05	1.013231135	1.00716171	1.01933713
SRF	0.00963989	1.017731635	1.00427689	1.03136664
TCF3	2.65E-07	1.034445334	1.02118991	1.04787282
TCF12	0.03514997	1.026324755	1.00181065	1.05143872
XRCC5	1.99E-06	1.00886442	1.00520082	1.01254137
CBFB	0.00014901	1.029380457	1.01409126	1.04490017
PSMF1	0.00988931	1.008522715	1.0020408	1.01504656
SETD1A	0.01333261	1.038936094	1.00797737	1.07084568
PSMD6	1.04E-06	1.070640621	1.04171039	1.1003743
ETV5	2.36E-06	1.030209822	1.01755563	1.04302138
WWTR1	0.00116065	1.012963162	1.00512165	1.02086585
GNL3	6.49E-05	1.013931815	1.00707174	1.02083862
ING2	0.00013541	1.050899682	1.02444396	1.07803861
TGFB2	0.00610694	1.0132364	1.00375732	1.02280499

TIAL1	0.00138881	1.039883512	1.01524741	1.06511744
FZD7	8.70E-05	1.025871714	1.01286766	1.03904273
CUL3	0.00555874	1.054379587	1.01564305	1.09459353
NUDT21	0.00030619	1.031595286	1.01431949	1.04916532
PWP1	2.31E-05	1.02759383	1.01472154	1.04062941
DHX36	0.00868779	1.069487377	1.0171479	1.12452009
GATA4	7.08E-07	1.021626735	1.01302394	1.03030258
TACSTD2	0.0347673	1.005283423	1.00037722	1.01021368
SOX6	4.37E-05	1.274446786	1.13452081	1.43163052
ELAVL1	0.00018114	1.036244918	1.01710982	1.05574001
CNOT1	0.03372947	1.015214645	1.0011622	1.02946434
LOXL2	0.02251636	1.014076912	1.00197222	1.02632784
CNOT3	8.34E-05	1.037568206	1.01868015	1.05680648
ZNF706	0.02550831	1.01514891	1.00184376	1.02863077
KDM3A	0.00098537	1.056202249	1.0223985	1.09112366
IGF2BP1	0.0126434	1.021517616	1.00456677	1.03875449
FANCD2	3.05E-06	1.132144209	1.07465084	1.19271345
FOXO3	0.01504488	1.023349446	1.00448453	1.04256866
SRRT	9.54E-09	1.021464393	1.0140823	1.02890023
MCPH1	0.00291903	1.199430034	1.06405785	1.35202461
FUT10	0.02770625	1.120006514	1.01250837	1.23891775
PDCD6	2.69E-06	1.036683101	1.02120224	1.05239864
SEMA4F	0.00208677	1.12053548	1.04219497	1.20476475
CFL1	2.01E-08	1.003140686	1.00204253	1.00424005
MSI2	0.00799765	1.06253305	1.01595686	1.11124451
EDN1	0.03549929	1.025841245	1.00173244	1.05053028
EDNRA	0.02718301	1.054543412	1.0060026	1.10542637
EFNB1	3.13E-07	1.016735194	1.01029055	1.02322095
TAPT1	0.02546812	0.954931746	0.91707143	0.99435508
ESR1	0.03392927	0.88571021	0.79174098	0.99083235
MTF2	9.66E-08	1.088037846	1.05481664	1.12230534
FN1	0.00332647	1.00054727	1.00018188	1.00091279
CORO1C	1.02E-06	1.019857465	1.01184792	1.02793041
GREM1	0.04503927	1.022654692	1.00049553	1.04530464
SETD2	0.01466759	1.031029786	1.00603489	1.05664568
HOXA7	0.00105956	2.124876539	1.35328699	3.33639525
HTR2B	0.03042637	0.952802118	0.91198887	0.99544183
JARID2	0.03234774	1.044444176	1.00366666	1.08687842
LAMA5	0.01980506	1.007601372	1.00120342	1.01404021
NRTN	0.02719138	1.012534194	1.00140407	1.02378803
PDGFRA	0.02697722	1.015415391	1.00174362	1.02927376
PITX2	0.00209322	1.097435658	1.03432261	1.16439978
SEMA5B	0.00684334	1.057582543	1.01553004	1.10137641
SEMA4C	0.00098663	1.031646881	1.01269975	1.0509485
PEF1	5.13E-06	1.012762055	1.00725621	1.01829799
MAPK1	0.00122852	1.024994627	1.00976452	1.04045445

MAPK3	2.28E-05	1.026887964	1.01435847	1.03957222
SEMA6A	8.84E-05	1.023633713	1.0117495	1.03565752
KLHL12	5.30E-05	1.0236029	1.01208922	1.03524757
RET	0.00725282	1.051465117	1.01364377	1.09069766
SEMA3F	0.00335899	1.035598964	1.0116709	1.06009297
BMPR1A	0.00507507	1.122950407	1.03546846	1.2178233
PHACTR4	0.00775885	1.028964566	1.00756165	1.05082213
TCOF1	7.31E-11	1.082572897	1.05703577	1.10872698
SETD6	0.00078989	1.141120344	1.05645742	1.23256803
SEMA6D	0.01865741	1.095605819	1.01534577	1.18221019
FZD1	0.00061647	1.020775861	1.00883201	1.03286112
TEAD2	0.00838508	1.006730835	1.00172221	1.01176451
RUNX2	0.03284115	1.058121524	1.00461852	1.11447394
NRP2	0.02690938	1.015820357	1.00179529	1.03004177
NRP1	0.00725421	1.009304772	1.00250396	1.01615172
ACVR1	0.00148007	1.01726282	1.00658333	1.02805562
SEMA5A	0.01225102	1.050635957	1.01080278	1.09203886
NOLC1	0.00044709	1.014516519	1.00638619	1.02271253
PUM1	0.00054486	1.02661147	1.01144115	1.04200933
SEMA3E	0.03313201	1.039939132	1.00313932	1.07808893
NR6A1	4.93E-07	1.119416411	1.07126872	1.16972807
HHEX	0.00428175	1.006826976	1.0021379	1.01153799
CD44	0.01986461	1.005516668	1.0008719	1.01018299
EZH2	1.75E-11	1.065242195	1.04580251	1.08504323
KDM5B	0.00029806	1.042870213	1.01941652	1.06686351

C in the TCGA HCC dataset

Supplementary Table S3. Protein-protein interaction pairs with a score higher than 0.7

#node1	node2	combined_score
ASS1	OTC	0.999
SERPINC1	F9	0.999
TNNI3	TNNC1	0.999
RBP4	TTR	0.998
EPHA5	EFNA5	0.998
EPHA7	EFNA5	0.998
C8B	C8A	0.997
EPHB3	EFNA5	0.997
PNLIP	CLPS	0.997
APOA2	APOC3	0.996
HBA1	HBA2	0.995
UGT1A10	UGT1A8	0.995
CPS1	ASS1	0.995
SSTR3	SST	0.995
TAT	HPD	0.994
DUOX2	DUOXA2	0.994
TNNT1	TNNI3	0.993
EPHA6	EFNA5	0.993
FZD1	WNT3A	0.992
EPHB6	EFNA5	0.992
TDRD9	PIWIL4	0.991
APOA5	APOC3	0.991
CPS1	OTC	0.991
APOA4	APOC3	0.99
DSC3	PKP3	0.99
ESR1	IGF1R	0.989
AFP	APOA2	0.989
GAST	GRP	0.989
MUC5AC	MUC1	0.989
TAC1	NTS	0.988
PIWIL4	TDRD1	0.988
MMP3	MMP10	0.987
ESR1	TFF1	0.987
NRG3	ERBB4	0.987
UROC1	AMDHD1	0.987
HSD17B6	CYP17A1	0.986
TNNT1	TNNC1	0.986
HSD17B6	CYP19A1	0.986
TAC1	GRP	0.985
MUC1	MUC6	0.985
WNT3A	SFRP2	0.984
GRIP1	GRIA2	0.983
GRP	NTS	0.983
BMP7	FST	0.982

ZNRF3	RSPO4	0.982
SGPP2	SPHK1	0.981
APOA2	LPA	0.981
GABRA5	GABRB3	0.981
ERBB4	EREG	0.98
MUC1	MUC5B	0.98
TDRD5	TDRD9	0.98
SNAP91	AMPH	0.979
SST	GAL	0.979
PTHLH	CALCA	0.978
FZD1	WNT9A	0.978
FZD7	WNT3A	0.978
SPTA1	ANK1	0.977
HP	HBA1	0.977
GAST	NTS	0.977
HP	HBA2	0.976
ARG1	OTC	0.976
PFKP	ALDOB	0.975
MUC1	GALNT4	0.975
WNT7B	FZD1	0.975
CYP1A2	NAT2	0.972
ITGB4	ITGA3	0.972
FZD7	WNT9A	0.971
COL8A2	COL2A1	0.97
EYA1	SIX2	0.97
APOA5	SERPINC1	0.97
GAST	TAC1	0.969
APOA5	APOA2	0.969
CYP3A4	UGT2B7	0.969
LPA	LCAT	0.969
WIF1	WNT9A	0.968
AMPH	SH3GL3	0.968
ESR1	TERT	0.968
FZD1	WIF1	0.968
CYP1A1	ARNT2	0.967
LPA	APOC3	0.967
GAD2	SNAP25	0.967
COL2A1	COL9A2	0.966
CPLX2	SNAP25	0.966
APOA5	AFP	0.966
NR1I3	CYP1A2	0.966
CYP2E1	NAT2	0.966
APOA2	LCAT	0.966
TDRD9	TDRD1	0.965
WIF1	WNT3A	0.965
RBP4	RBP2	0.965

APOC1	APOC3	0.964
SGPP2	DEGS2	0.964
WNT7B	FZD7	0.964
APOA2	APOA4	0.964
ESR1	NR0B1	0.963
APOA2	SERPINC1	0.963
CYP1A1	GSTM1	0.962
SPHK1	ACER1	0.962
NMU	NTS	0.962
ITGB8	ITGA3	0.962
ESR1	CYP19A1	0.962
AFP	SERPINC1	0.962
CYP3A4	NR1I2	0.962
TDRD5	PIWIL4	0.962
F12	F9	0.961
CYP3A4	UGT1A8	0.961
CNTN1	L1CAM	0.961
CTH	BHMT	0.961
GABRA3	GABRB3	0.96
GABRA2	GABRB3	0.96
ESR1	TFAP2A	0.96
C8A	C9	0.96
CYP2E1	ADH4	0.958
LAMC2	ITGB4	0.958
SERPINC1	F12	0.958
APOA2	FABP1	0.957
CYP2E1	NR1I3	0.957
L1CAM	ANK1	0.957
APOC1	APOA2	0.957
C8A	C6	0.957
LAMA1	ITGA3	0.956
CCL25	CXCL5	0.956
NRG3	EREG	0.956
C8B	C9	0.956
PKM	PCK2	0.955
CYP2A6	NAT2	0.955
LAMC2	ITGA3	0.955
MUC6	A4GNT	0.955
GNRH2	KISS1R	0.955
GAL	NMU	0.954
APOC4	APOC1	0.954
CCL25	CXCL6	0.954
ITGB6	ITGA3	0.954
CYP2E1	ALDH3B2	0.954
APOA4	LPA	0.953
RDH16	CYP26A1	0.953

CALCA	SCTR	0.953
APOA5	FABP1	0.952
CYP1A2	CYP3A4	0.952
HAO2	DAO	0.952
AFP	BPIFB2	0.951
MSX2	DLX5	0.951
GRIN2A	GRIA2	0.951
BCAT1	CTH	0.951
GAST	SST	0.951
MASP2	FCN2	0.951
UGT8	DEGS2	0.951
PKM	PCK1	0.95
CYP1A1	CYP3A4	0.95
TUBA3C	ENSG000002	0.95
SNAP91	SH3GL3	0.949
CYP3A4	CYP2A6	0.949
CYP19A1	CYP17A1	0.949
ESR1	GRIP1	0.948
CCL16	CXCL5	0.948
DEGS2	ACER1	0.948
CYP3A4	CYP2B6	0.948
PTHLH	SCTR	0.947
GABBR1	SST	0.947
CTH	CDO1	0.947
CYP2B6	SULT2A1	0.947
NEFL	GRIA2	0.947
GLP1R	CALCA	0.946
TREH	GCK	0.946
APOA2	PON1	0.946
CYP2E1	CYP3A4	0.946
SGPP2	ACER1	0.946
CCL16	CXCL6	0.946
B3GALT5	FUT3	0.946
MUC5AC	A4GNT	0.945
ZNRF3	WNT3A	0.945
IDO2	TPH1	0.945
ESR1	TGFA	0.945
CCL16	CCL25	0.945
WNT3A	DKK4	0.944
NMU	GRP	0.944
DUOX2	DUOXA1	0.944
CTH	SDS	0.944
TFAP2A	PITX2	0.944
TAC1	SST	0.943
HPR	HP	0.943
OXTR	GNRH2	0.943

HSD17B6	CYP1A1	0.943
HSD17B6	CYP3A4	0.943
OXTR	NTS	0.943
MUC1	GALNT3	0.943
FZD1	DKK4	0.943
CYP2A7	CYP2A6	0.943
SNAP91	SYT9	0.942
CYP2J2	CYP4F2	0.942
MUC5AC	GALNT4	0.942
ANK1	CHL1	0.942
SPHK1	DEGS2	0.941
CYP19A1	CYP1A1	0.941
ALDOB	TKTL1	0.941
HTR2C	NTS	0.941
CDH10	CDH9	0.941
APOA4	RBP2	0.941
MUC5AC	MUC5B	0.94
NEFL	GRIN2A	0.94
KRT4	KRT15	0.94
CYP3A4	CYP2A13	0.94
CDH15	CDH9	0.94
CRISP3	OLFM4	0.939
CDH18	CDH9	0.939
CDH18	CDH12	0.939
CRHR1	CALCA	0.939
KRT80	KRT15	0.938
TAC1	HTR2C	0.938
CYP2J2	CYP3A4	0.938
KRT4	KRT19	0.938
MUC5AC	GALNTL6	0.938
LAMA1	ITGB4	0.937
APOA5	APOA4	0.937
CDH18	CDH6	0.937
GABRA3	GABRA5	0.937
MUC1	GALNTL6	0.937
SGPP2	UGT8	0.937
CYP19A1	CYP3A4	0.937
GCGR	CALCA	0.937
GAD2	GLS2	0.937
GRM1	HTR2C	0.937
ALDOB	PKM	0.936
CDH6	CDH9	0.936
COL9A2	COL11A1	0.936
CYP2B6	CYP4F2	0.936
CDH6	CDH10	0.936
C8B	C6	0.935

COL2A1	COL11A1	0.935
KRT80	KRT20	0.935
LCAT	PON1	0.935
NTS	KISS1R	0.935
GRM1	TAC1	0.935
CYP17A1	CYP3A4	0.935
MUC5AC	GALNT5	0.935
CYP2E1	CYP4F2	0.935
CLDN10	CLDN18	0.934
KRT80	KRT19	0.934
MUC5AC	GALNT3	0.934
CYP2B6	CYP26A1	0.934
CYP1A1	CYP26A1	0.934
NLGN1	GRM1	0.934
AVPR1A	NTS	0.934
SAA1	CXCL5	0.934
GABBR1	GRM3	0.934
ARHGAP36	RHOV	0.934
CYP1A1	SULT2A1	0.934
TAC1	NMU	0.934
MUC1	GALNT5	0.934
CRISP3	TCN1	0.933
GCGR	ADCY1	0.933
WNT3A	WNT9A	0.933
ADH4	CYP26A1	0.932
SST	NMU	0.932
CYP1A2	CYP2A6	0.932
ARG1	HP	0.932
REG3A	REG3G	0.932
CRABP2	RXRG	0.932
CYP3A4	CYP26A1	0.932
IDO2	CYP1A1	0.931
RAB19	RAB36	0.931
RAB25	RAB36	0.931
APOA2	BPIFB2	0.931
GRM1	AVPR1A	0.931
KLC3	KIF3C	0.93
APOA5	BPIFB2	0.93
LPA	PON1	0.93
DLX6	DLX5	0.93
CYP1A1	HSD11B1	0.93
CYP1A2	CYP2B6	0.93
CYP2E1	CYP1A2	0.93
REG1B	REG1A	0.93
CALCA	TAC1	0.929
CYP2B6	CYP26B1	0.929

RAB19	RAB25	0.929
SSTR3	GAL	0.929
FGF19	FGF4	0.929
CYP1A1	CYP26B1	0.929
HSD11B1	CYP3A4	0.929
ARHGEF38	RHOV	0.929
PFKP	TKTL1	0.929
CYP1A2	CYP26A1	0.928
GALNTL6	MUC6	0.928
PFKFB3	PFKFB1	0.928
MUC5AC	MUC6	0.928
PFKP	PKM	0.928
KRT4	KRT12	0.928
CYP2A6	CYP26A1	0.928
ITPR3	SNAP25	0.928
CYP1A1	CYP2B6	0.928
COL9A2	MMP3	0.928
KRT4	KRT20	0.928
COL9A2	MMP13	0.928
C6	C9	0.928
TAC1	KISS1R	0.928
MUC5B	MUC6	0.928
CYP2E1	GSTM1	0.927
TCN1	OLFM4	0.927
CYP2E1	CYP1A1	0.927
APOA2	HP	0.927
CRHR1	ADCY1	0.926
GRM3	ADCY1	0.926
GALNT4	MUC6	0.926
PTHLH	GCGR	0.926
NMU	GNRH2	0.926
COL8A2	COL11A1	0.926
LPAR3	NTS	0.926
CERS1	DEGS2	0.926
APOC1	LCAT	0.926
LAMC2	MMP7	0.925
CDH12	CDH17	0.925
GRM1	NTS	0.925
CYP3A4	CYP26B1	0.925
GIPR	CALCA	0.924
NKX3-2	MSX2	0.924
GRM3	GRM1	0.924
CDH15	CDH17	0.924
MUC5B	GALNTL6	0.924
CYP1A1	CYP2A6	0.924
CDH6	CDH17	0.924

CYP1A2	CYP2A13	0.924
PKM	LDHD	0.924
LPAR2	NTS	0.923
LYPD6B	LY6K	0.923
SAA1	CXCL6	0.923
AVPR1A	GRP	0.923
MUC6	GALNT5	0.923
GAST	NMU	0.923
FABP1	SULT2A1	0.923
KRT19	KRT15	0.922
NKX3-2	DLX5	0.922
BMP7	NKX2-5	0.922
SSTR3	GABBR1	0.922
MUC6	GALNT3	0.922
TKTL1	PKM	0.922
CYP1A1	CYP2A13	0.922
CYP2A6	CYP26B1	0.922
CPS1	GLS2	0.922
GCGR	GRM1	0.922
CALCA	GPR27	0.922
CXCL5	CXCL6	0.921
CYP1A2	CYP26B1	0.921
GLP1R	ADCY1	0.921
GABBR1	ADCY1	0.921
CYP1A1	CYP17A1	0.921
SCN1A	ANK1	0.921
CYP2E1	CYP17A1	0.921
FZD1	SFRP2	0.921
CDH18	CDH17	0.921
DLX6	NKX3-2	0.92
CDH10	CDH17	0.92
CYP2E1	HSD11B1	0.92
CYP17A1	CYP1A2	0.92
CDH9	CDH17	0.92
COL8A2	COL9A2	0.919
DLX6	MSX2	0.919
MAGEA12	CSAG1	0.919
XPNPEP2	ALPI	0.919
PNLIP	RBP2	0.919
GCGR	NTS	0.919
KRT80	KRT12	0.919
PPFIA4	SNAP25	0.919
TAT	BHMT	0.919
HSD11B1	CYP2A6	0.919
GRM1	OXTR	0.919
CYP2J2	CYP1A2	0.919

FABP1	ABCB4	0.918
UGT8	ACER1	0.918
GAD2	CDO1	0.918
OXTR	TAC1	0.918
GRM3	SST	0.918
CYP19A1	FOXL2	0.918
NTNG1	GPLD1	0.918
MUC5B	GALNT4	0.917
APOA5	LPA	0.917
HSD11B1	CYP2A13	0.917
AFP	TNC	0.917
CYP2B6	UGT1A8	0.917
MMP3	LAMC2	0.917
GABBR1	GAL	0.917
TAC1	AVPR1A	0.916
MUC5B	GALNT5	0.916
CDH18	CDH10	0.916
SERPINC1	BPIFB2	0.916
TFF1	TFF2	0.916
AMPH	SYT9	0.916
LAMC2	MMP13	0.915
LAMA1	LAMC2	0.915
CYP1A1	FABP1	0.915
PTHLH	GLP1R	0.915
EPHA6	EPHA7	0.915
PLXNB3	SEMA3C	0.915
KRT12	KRT20	0.915
PTHLH	ADCY1	0.915
MUC5B	GALNT3	0.914
PLXNB3	SEMA3E	0.914
MUC5B	A4GNT	0.914
GABBR1	KCNJ4	0.914
PROK1	GRP	0.914
CTNNA2	CDH15	0.914
CYP2E1	CYP2A6	0.914
GGT6	GAD2	0.914
GAL	MTNR1B	0.914
SERPINB3	TTR	0.914
GIPR	PTHLH	0.914
GRM1	GRP	0.914
LGI1	CACNG4	0.914
SST	MTNR1B	0.914
FXYD1	ATP1A3	0.914
GCGR	GAST	0.914
EPHA5	EPHA6	0.914
MUC1	CHST4	0.913

SSTR3	LPAR2	0.913
GAD2	CNDP1	0.913
ITPR3	ANK1	0.913
GCGR	CRHR1	0.913
PAK7	MUSK	0.913
CALCA	ADCY1	0.913
MMP10	MMP7	0.913
CRHR1	GPR27	0.913
MT1G	MT1H	0.913
COL28A1	COL2A1	0.913
LPAR3	TAC1	0.912
P2RY4	GRM3	0.912
CDH18	CDH15	0.912
MATN3	AFP	0.912
NMU	MTNR1B	0.912
HTR2C	GRP	0.912
ABCB11	SLC10A1	0.912
FXYD3	ATP1A3	0.912
SSTR3	GRM3	0.912
PTHLH	CRHR1	0.912
WNT7B	WNT9A	0.912
MATN3	TNC	0.911
GABRA2	GABRA3	0.911
CRHR1	GLP1R	0.911
ADCY1	SCTR	0.911
CYP2B6	CYP2A6	0.911
CYP2E1	CYP2J2	0.911
GRM1	KISS1R	0.911
KRT19	KRT20	0.911
OXTR	GRP	0.911
AOC1	HP	0.911
GABBR1	P2RY4	0.911
GABRA2	GABRA5	0.91
CDH12	CDH9	0.91
MMP3	MMP13	0.91
CNDP1	ALDH3B2	0.91
APOC1	LPA	0.91
CYP2E1	CYP2B6	0.91
IDO2	CYP1A2	0.91
CYP2J2	CYP2B6	0.91
CRHR1	SCTR	0.91
GAD2	ALDH3B2	0.91
GIPR	ADCY1	0.91
MMP3	MMP7	0.91
CDH12	CDH15	0.91
CEACAM7	PRSS21	0.91

OXTR	HTR2C	0.91
APOC1	APOA5	0.909
GPR27	ADCY1	0.909
CDH15	CDH10	0.909
GCGR	OXTR	0.909
ADCY1	SST	0.909
ALPI	GPLD1	0.909
SYT9	SH3GL3	0.909
ARG1	SERPINB3	0.909
GPR27	SCTR	0.909
CRISP3	TNFAIP6	0.909
MMP10	MMP13	0.909
SSTR3	ADCY1	0.909
CDH15	CDH6	0.909
EPHA5	EPHA7	0.909
XPNPEP2	ART3	0.909
FUT4	FUT3	0.909
KRT15	KRT20	0.909
GRM3	GAL	0.909
LPAR3	ADCY1	0.908
GRP	KISS1R	0.908
APOA2	TNC	0.908
CYP2E1	CYP2A13	0.908
ADCY1	MTNR1B	0.908
LPAR2	SST	0.908
SYT9	CFTR	0.908
CHRNA4	CHRND	0.908
LCAT	APOC3	0.908
LDHD	OTC	0.908
TAC1	GNRH2	0.908
COL28A1	COL9A2	0.908
TFAP2A	TGFA	0.908
PRSS1	MMP7	0.908
KRT4	KRT80	0.908
GRM3	MTNR1B	0.908
GIPR	CRHR1	0.908
ARHGAP40	RHOV	0.908
CYP1A1	UGT1A8	0.908
ETNK2	ETNPPL	0.907
HTR2C	NMU	0.907
CDH12	CDH10	0.907
BPIFB2	TMEM132A	0.907
GABBR1	MTNR1B	0.907
CNTN5	NTNG1	0.907
CYP2A13	CYP2A6	0.907
ARG1	TCN1	0.907

LPAR2	MTNR1B	0.907
GNMT	DAO	0.907
GIPR	GLP1R	0.907
COL28A1	COL11A1	0.907
CRISP3	ARG1	0.907
TNC	TMEM132A	0.907
MMP13	MMP7	0.907
WNT7B	WNT3A	0.907
MATN3	BPIFB2	0.907
TCN1	TNFAIP6	0.907
ENPP7	UGT8	0.907
GLP1R	SCTR	0.906
LYPD6B	NTNG1	0.906
CRISP3	HP	0.906
GCGR	GNRH2	0.906
ALPI	PRSS21	0.906
GIPR	SCTR	0.906
KRT19	KRT12	0.906
ARG1	TTR	0.906
LPAR2	GABBR1	0.906
LPAR2	KISS1R	0.906
PKM	ENTPD3	0.906
GCGR	SCTR	0.906
MUC1	A4GNT	0.906
SSTR3	MTNR1B	0.905
CYP1A2	UGT1A8	0.905
LPAR2	LPAR3	0.905
RNF182	ASB11	0.905
RNF182	UBE2U	0.905
HP	TCN1	0.905
APOA2	TMEM132A	0.905
GAST	GNRH2	0.905
MATN3	APOA2	0.905
EPHB6	EPHA6	0.905
GCGR	GRP	0.905
ITGB6	TNC	0.905
CDH12	CDH6	0.905
SPAG6	SPAG17	0.905
ASB11	UBE2U	0.905
GCGR	GLP1R	0.905
TAC1	PROK1	0.905
NMU	KISS1R	0.905
OXTR	AVPR1A	0.905
LY6K	PRSS21	0.905
GABBR1	NMU	0.905
OXTR	NMU	0.905

SPTA1	SCN1A	0.905
AVPR1A	KISS1R	0.905
MUC6	CHST4	0.904
AOC1	OLFM4	0.904
PRSS1	MMP13	0.904
XPNPEP2	PRSS21	0.904
FBN2	ITGB6	0.904
GP2	ALPI	0.904
EPHB6	EPHA5	0.904
MATN3	SERPINC1	0.904
LPAR2	GRM3	0.904
AOC1	ARG1	0.904
GIPR	GCGR	0.904
EPHA6	EPHB3	0.904
AVPR1A	HTR2C	0.904
P2RY4	SST	0.904
EPHB6	EPHB3	0.904
GCGR	TAC1	0.904
LPAR2	ADCY1	0.904
PROK1	KISS1R	0.904
EPHB6	EPHA7	0.904
UPP2	CYP2A6	0.904
GAST	OXTR	0.904
SAA1	NMU	0.904
KRT15	KRT12	0.903
LOC93432	SI	0.903
XPNPEP2	GPLD1	0.903
GRM1	GAST	0.903
HP	OLFM4	0.903
PKP3	PI3	0.903
SAA1	SST	0.903
LPAR3	SST	0.903
GCGR	AVPR1A	0.903
LPAR3	P2RY4	0.903
MATN3	TMEM132A	0.903
LPAR2	GRM1	0.903
LPAR3	GAL	0.903
GP2	PRSS21	0.903
MUC5AC	CHST4	0.903
LPAR3	GRP	0.903
FOLR1	SPTA1	0.903
FZD1	FZD7	0.903
AMBN	BPIFB2	0.903
SAA1	NTS	0.903
LPAR3	GCGR	0.903
TNC	BPIFB2	0.903

P2RY4	GAL	0.903
CNTN5	LYPD6B	0.902
SAA1	P2RY4	0.902
OXTR	KISS1R	0.902
APOA5	MATN3	0.902
GRM3	NMU	0.902
OR13A1	RTP3	0.902
NTNG1	ALPI	0.902
CNTN5	PRSS21	0.902
EPHA7	EPHB3	0.902
HP	TNFAIP6	0.902
GCGR	PROK1	0.902
AVPR1A	PROK1	0.902
AFP	AMBN	0.902
LPAR3	SAA1	0.902
MUC1	B3GNT7	0.902
OR7D2	RTP3	0.902
L1CAM	SPTA1	0.902
HAMP	EPO	0.902
P2RY4	ADCY1	0.902
SSTR3	NMU	0.902
SAA1	GCGR	0.902
LAMA1	L1CAM	0.902
LPAR2	TAC1	0.902
GCGR	KISS1R	0.902
ART3	GPLD1	0.902
ALPI	CEACAM7	0.902
PRSS1	MMP3	0.902
PAK7	LAMC2	0.902
HTR2C	KISS1R	0.902
AOC1	CRISP3	0.902
GP2	XPNPEP2	0.902
GAST	KISS1R	0.902
EPHA5	EPHB3	0.902
CNTN5	GPLD1	0.902
MYO10	CALML3	0.902
LPAR2	P2RY4	0.902
APOA2	RBP2	0.902
LPAR3	OXTR	0.902
TNFAIP6	OLFM4	0.901
SERPINC1	LPA	0.901
ARG1	OLFM4	0.901
CASP14	PI3	0.901
SSTR3	P2RY4	0.901
SAA1	GABBR1	0.901
GCGR	NMU	0.901

LPAR3	NMU	0.901
SSTR3	LPAR3	0.901
P2RY4	MTNR1B	0.901
LPAR2	HTR2C	0.901
NTS	GNRH2	0.901
LPAR3	GRM1	0.901
GRM1	GNRH2	0.901
SAA1	GRM1	0.901
FOLR1	TGFA	0.901
LPAR2	GNRH2	0.901
NTNG1	PRSS21	0.901
LPAR3	AVPR1A	0.901
LY6K	GPLD1	0.901
FOLR1	ANK1	0.901
P2RY4	CXCL5	0.901
AMBN	TNC	0.901
DSC3	PI3	0.901
ART3	ALPI	0.901
LPAR2	NMU	0.901
KCNJ16	GABBR1	0.901
OR52E8	RTP3	0.901
LPAR3	GNRH2	0.901
SNAP91	CFTR	0.901
GAST	HTR2C	0.901
CXCL5	SST	0.901
PRSS1	TCN1	0.901
LPAR3	GRM3	0.901
GCGR	HTR2C	0.901
P2RY4	CXCL6	0.901
LPAR2	GAST	0.9
LPAR3	PROK1	0.9
LYPD6B	ALPI	0.9
TGFB2	HRG	0.9
ADCY1	GAL	0.9
CCL16	MTNR1B	0.9
SERPINC1	TMEM132A	0.9
GRM3	CXCL5	0.9
COL28A1	COL8A2	0.9
MATN3	AMBN	0.9
CCL25	GRM3	0.9
SSTR3	CXCL6	0.9
SST	CXCL6	0.9
CCL25	MTNR1B	0.9
TRIM17	MT2A	0.9
CCL25	ADCY1	0.9
HTR2C	GNRH2	0.9

CXCL5	GAL	0.9
PROK1	GNRH2	0.9
ADCY1	CXCL5	0.9
CCL25	GAL	0.9
SAA1	HTR2C	0.9
LPAR2	GCGR	0.9
APOA5	TMEM132A	0.9
GIPR	GPR27	0.9
SAA1	AVPR1A	0.9
SAA1	GNRH2	0.9
PROK1	NMU	0.9
GP2	ART3	0.9
SAA1	KISS1R	0.9
LPAR3	CXCL5	0.9
CNTN5	GP2	0.9
LYPD6B	GP2	0.9
LPAR2	PROK1	0.9
LYPD6B	XPNPEP2	0.9
NTNG1	LY6K	0.9
CNTN5	ART3	0.9
CCL16	LPAR2	0.9
SAA1	PROK1	0.9
CCL25	P2RY4	0.9
PRSS1	MMP10	0.9
GP2	LY6K	0.9
MCEMP1	SNAP25	0.9
GP2	CEACAM7	0.9
GRM3	CXCL6	0.9
PROK1	NTS	0.9
SAA1	MTNR1B	0.9
AVPR1A	NMU	0.9
APOC1	APOA4	0.9
GRM1	PROK1	0.9
NTNG1	ART3	0.9
MUC5B	B3GNT7	0.9
LPAR2	SAA1	0.9
CCL16	SST	0.9
CCL16	GAL	0.9
AVPR1A	GNRH2	0.9
LPAR3	GABBR1	0.9
MTNR1B	CXCL6	0.9
GABBR1	CXCL6	0.9
GABBR1	CXCL5	0.9
ART3	CEACAM7	0.9
ADCY1	NMU	0.9
MUC6	B3GNT7	0.9

GP2	NTNG1	0.9
CNTN5	CEACAM7	0.9
LPAR2	GRP	0.9
SAA1	TAC1	0.9
CCL16	NMU	0.9
XPNPEP2	CEACAM7	0.9
LPAR2	CCL25	0.9
APOA5	TNC	0.9
LPAR3	GAST	0.9
P2RY4	NMU	0.9
GRM1	NMU	0.9
LPAR3	CXCL6	0.9
APOA2	AMBN	0.9
GLP1R	GPR27	0.9
LYPD6B	ART3	0.9
LYPD6B	PRSS21	0.9
CCL25	NMU	0.9
SAA1	OXTR	0.9
PTHLH	GPR27	0.9
CCL16	GABBR1	0.9
OXTR	PROK1	0.9
SAA1	CCL25	0.9
APOA5	AMBN	0.9
XPNPEP2	NTNG1	0.9
SERPINC1	AMBN	0.9
XPNPEP2	LY6K	0.9
SAA1	ADCY1	0.9
GRP	GNRH2	0.9
SSTR3	CXCL5	0.9
ALPI	LY6K	0.9
GPLD1	CEACAM7	0.9
SSTR3	SAA1	0.9
CNTN5	ALPI	0.9
LYPD6B	CEACAM7	0.9
SSTR3	CCL16	0.9
CCL16	GRM3	0.9
ART3	PRSS21	0.9
CCL25	GABBR1	0.9
LPAR2	GAL	0.9
CNTN5	LY6K	0.9
SERPINC1	TNC	0.9
ADCY1	CXCL6	0.9
LPAR3	MTNR1B	0.9
LPAR2	CXCL6	0.9
CXCL5	MTNR1B	0.9
GAL	CXCL6	0.9

GAST	PROK1	0.9
ART3	LY6K	0.9
CNTN5	XPNPEP2	0.9
CCL25	SST	0.9
AFP	TMEM132A	0.9
LAMA1	PAK7	0.9
GAST	AVPR1A	0.9
GP2	GPLD1	0.9
CCL16	ADCY1	0.9
AMBN	TMEM132A	0.9
HTR2C	PROK1	0.9
CXCL5	NMU	0.9
NTNG1	CEACAM7	0.9
SH3GL3	CFTR	0.9
LPAR3	CCL25	0.9
SAA1	GRM3	0.9
LPAR3	KISS1R	0.9
GPLD1	PRSS21	0.9
LY6K	CEACAM7	0.9
GCGR	GPR27	0.9
MUC5B	CHST4	0.9
MUC5AC	B3GNT7	0.9
LPAR3	HTR2C	0.9
CCL16	LPAR3	0.9
LPAR2	AVPR1A	0.9
NMU	CXCL6	0.9
SAA1	GAL	0.9
SAA1	GAST	0.9
CCL16	SAA1	0.9
SSTR3	CCL25	0.9
AMPH	CFTR	0.9
SAA1	GRP	0.9
LPAR2	OXTR	0.9
LPAR2	CXCL5	0.9
AOC1	TCN1	0.9
CCL16	P2RY4	0.9
LYPD6B	GPLD1	0.9
APOA4	HP	0.899
HP	HPX	0.898
WNT3A	SFRP5	0.898
APOC3	PON1	0.894
TDRD5	TDRD1	0.893
SST	CHGA	0.892
WNT7B	SFRP5	0.892
BCAT1	SDS	0.891
UGT1A10	CYP2B6	0.89

ERBB4	TGFA	0.888
ALDOB	GCK	0.888
UGT2B7	CYP2A6	0.887
CYP2B6	UGT2B7	0.886
APOF	APOA2	0.884
CST1	CST4	0.883
APOA4	LCAT	0.883
KCNJ4	KCNQ1	0.882
CYP1A2	UGT2B7	0.882
MAGEA6	CSAG1	0.882
SERPINC1	PROZ	0.881
WNT9A	SFRP5	0.88
APOA5	LCAT	0.88
SERPINC1	HRG	0.879
PCDHA11	PCDHA10	0.878
PNMA2	DPYSL5	0.878
FABP1	APOC3	0.877
HP	APOC3	0.875
CYP2A6	UGT2B10	0.875
CYP1A2	GSTM1	0.875
HP	PON1	0.874
WNT7B	SFRP2	0.874
AMPH	DPYSL5	0.873
SFRP2	WNT9A	0.873
BHMT	SDS	0.872
EFNA5	NTRK2	0.872
APOC4	APOC3	0.872
APOC1	PON1	0.872
HTR3A	HTR2C	0.871
SYT9	SNAP25	0.871
CYP1A1	UGT1A10	0.87
CYP1A1	UGT2B7	0.87
MTNR1B	GPR50	0.87
APOA4	PON1	0.868
PTF1A	RBPJL	0.868
CPA2	CPA1	0.867
SST	GRP	0.867
HP	LCAT	0.867
SERPINC1	FETUB	0.867
SHISA9	GRIA2	0.867
CYP2E1	UGT1A8	0.866
REG3A	REG1A	0.866
HPR	APOA2	0.865
HTR3A	CTTNBP2	0.86
SST	NTS	0.86
GAST	ATP12A	0.86

APOA5	PON1	0.86
GABBR1	GRM1	0.859
CALB2	SST	0.857
COL2A1	MMP13	0.856
UGT1A10	CYP3A4	0.856
PFKFB3	PFKP	0.856
PCK1	PCK2	0.856
HPR	HBA1	0.856
CALCA	SST	0.855
ESRP1	GRHL2	0.855
UGT1A8	CYP2A6	0.854
CYP2B6	GSTM1	0.853
HPR	PON1	0.853
TKTL1	GCK	0.853
CDX2	KRT20	0.85
CYP2E1	UGT2B7	0.849
APOC1	HP	0.848
UGT1A10	CYP1A2	0.847
CD207	CD1A	0.847
MUSK	CHRND	0.847
NR1I2	UGT1A8	0.844
WNT7B	WIF1	0.843
AFP	GPT	0.842
XPNPEP2	MEP1A	0.842
MUC5AC	TFF1	0.841
CYP3A4	GSTM1	0.841
SERPINC1	APOC3	0.84
SAA1	HP	0.84
GSTM1	CYP2A6	0.839
TAC1	GAL	0.839
GGT6	GLS2	0.838
HPR	APOC3	0.837
APOC4	APOA4	0.836
SNAP25	SYT13	0.835
SLC26A9	CFTR	0.835
HAVCR1	FABP1	0.834
GAST	CHGA	0.834
PCDHA6	PCDHA10	0.834
MT1G	MT1X	0.833
RTL1	DLK1	0.833
GLYAT	UGT8	0.832
PFKP	PFKFB1	0.832
MT1X	MT1H	0.831
SAA1	PCSK1N	0.831
MT1H	MT1E	0.831
HCN1	HCN4	0.831

MT1X	MT2A	0.831
MT1X	MT1E	0.83
ADH4	ALDH3B2	0.83
MIP	GJA3	0.829
GRIN2A	HTR3A	0.828
GNMT	BHMT	0.827
CYP26A1	CYP26B1	0.826
SAA4	APOC3	0.826
PDGFD	TGFA	0.825
SERPINC1	C9	0.824
GAD2	ASS1	0.824
SHD	CD300LG	0.822
APOA4	FABP1	0.822
NR1I3	CYP3A4	0.821
DCDC2	KAAG1	0.82
PIWIL4	RNF17	0.819
MCIDAS	FOXJ1	0.819
PNMA2	AMPH	0.818
APOC1	APOF	0.817
NR1I3	CYP2B6	0.817
HPX	HRG	0.814
CYP1A1	CYP1A2	0.813
GRIA2	CACNG4	0.813
SNTG1	SGCZ	0.813
MT1E	MT2A	0.812
BEX1	NHLH2	0.812
NR1I2	CYP2B6	0.812
CTNNA2	CTNND2	0.812
CYP8B1	ABCB11	0.811
AFP	KRT19	0.809
HPR	LCAT	0.808
NR1I3	UGT1A8	0.808
NR1I2	SULT2A1	0.806
GPX3	GSTM1	0.806
GRIN2A	GRM3	0.805
CNDP1	GLS2	0.805
ASPDH	ASS1	0.804
RORB	BHLHE41	0.804
CYP2E1	UGT1A10	0.803
PPP1R14D	PPP1R14C	0.803
MT1H	MT2A	0.803
EYA1	SIX3	0.802
LGI1	DPYSL5	0.802
SERPINC1	APOA4	0.8
APOC4	APOA2	0.8
APOF	APOC3	0.8

GPR123	SCTR	0.8
HP	TTR	0.799
CFAP221	SPAG6	0.799
CALCA	GAL	0.798
CA5A	CYP24A1	0.798
GSTM1	NAT2	0.797
HTR3A	GRIA2	0.797
TFDP3	HRK	0.797
HP	LPA	0.796
PTF1A	MNX1	0.795
APOF	PON1	0.794
ADIPOQ	RBP4	0.793
APOC4	PON1	0.793
AFP	NTS	0.793
HTR3A	TAC1	0.793
EFNA5	SEMA3E	0.792
EFNA5	SEMA3C	0.792
KCNU1	LRRC52	0.792
CALCA	GRP	0.791
HPR	APOA4	0.791
APOF	LCAT	0.791
CYP1A1	UGT2B10	0.791
SHISA9	GRIN2A	0.79
SLC30A8	GAD2	0.79
UGT1A10	CYP2A13	0.788
PCDHA11	PCDHA12	0.788
APOA5	HPR	0.788
PTF1A	CPA1	0.787
DCHS2	USH1C	0.787
ENPP7	ACER1	0.786
APOA2	SAA4	0.786
PRSS1	CFTR	0.786
ASS1	ARG1	0.785
SAA1	APOA2	0.785
CXCL17	CCL25	0.784
CADPS	GABRA3	0.784
MT1G	MT1E	0.784
APOC1	HPR	0.784
PCDHA6	PCDHA3	0.783
CYP2A13	CYP2A7	0.783
MT1G	MT2A	0.783
MT1A	MT1X	0.783
CYP19A1	UGT1A8	0.781
WIF1	SFRP5	0.781
CYP8B1	SLC10A1	0.781
SPAG6	FOXJ1	0.781

HPR	LPA	0.78
PRSS1	CPA1	0.778
FOXL2	FST	0.777
NKX3-2	PAX9	0.777
UGT1A10	CYP2A6	0.777
ITGB6	LAMC2	0.776
ITGB6	ITGB4	0.776
CYP2B6	UGT2B10	0.775
HTR2C	TPH1	0.775
CYP2A13	GSTM1	0.775
MAGEA6	MAGEA12	0.773
CYP2A13	UGT1A8	0.773
LPA	C9	0.773
MUC1	ESR1	0.772
ODAM	AMBN	0.772
FOXD1	SIX2	0.772
AMPH	DNER	0.771
APOF	LPA	0.771
GRIN2A	CACNG4	0.771
MASP2	C8B	0.77
CYP2A13	UGT2B10	0.77
APOA4	FETUB	0.769
WIF1	SFRP2	0.768
KCNA1	SCN1A	0.766
GAD2	CALB2	0.766
CTNND2	CDH17	0.766
NR0B1	FOXL2	0.766
APOC4	LCAT	0.766
APOC4	APOA5	0.764
SNAP91	SNAP25	0.764
PNMA2	DNER	0.764
TAT	SDS	0.763
APOA5	HP	0.763
PNLIP	CPA1	0.763
GRIN2A	GRM1	0.762
NKX2-5	HCN4	0.761
NTRK2	CHL1	0.761
FOXJ1	SCGB1A1	0.76
AGR2	TFF1	0.759
C19orf33	SPINT2	0.759
APOA5	APOF	0.759
ITGB8	ITGB4	0.759
GCK	PCK2	0.758
APOC4	LPA	0.758
SEC14L2	OTC	0.757
GAL	NTS	0.757

GSTM1	ADH4	0.755
MATN3	COL2A1	0.754
ESR1	ERBB4	0.754
CYP3A4	UGT2B10	0.753
CYP19A1	NR0B1	0.753
APOF	APOA4	0.753
SAA1	LPA	0.753
HSD17B6	UGT1A8	0.752
HSD17B6	UGT1A10	0.752
PNMA2	LGI1	0.751
GAST	TFF2	0.749
GAD2	SST	0.748
UCHL1	NTRK2	0.748
CA9	CYP24A1	0.748
ANO2	CFTR	0.747
TMEM252	CNDP1	0.747
MUC1	KRT19	0.747
CDX2	MEP1A	0.746
BPIFB1	BPIFB2	0.746
DZIP1L	CLDN18	0.746
DMRT2	FOXL2	0.746
F9	OTC	0.746
MAGEA12	GAGE2A	0.745
GRIA2	SNAP25	0.745
MUC5AC	CDX2	0.743
MTNR1B	GCK	0.743
APOA2	TTR	0.743
PLXNB3	PKHD1	0.742
CRABP2	CYP26A1	0.742
CYP2E1	UGT2B10	0.742
ITGB6	ITGB8	0.741
MUC6	CDX2	0.741
LGI1	AMPH	0.741
NLGN1	GRIA2	0.74
MASP2	C8A	0.739
ABCB4	SLC10A1	0.739
HSD17B6	SULT2A1	0.739
CYP2A13	UGT2B7	0.739
OTOG	USH1C	0.739
PCK1	GCK	0.739
LAMA1	ITGB6	0.739
CALCA	GAST	0.738
BPIFA2	BPIFB1	0.738
APOC1	SAA4	0.737
MT1A	MT1H	0.737
SAA1	PON1	0.737

BPIFA1	BPIFB2	0.736
GALP	GAL	0.736
GRM1	GRIA2	0.736
AFP	TTR	0.736
ESR1	FOXL2	0.735
GRIP1	FRAS1	0.735
SHISA9	HTR3A	0.734
APOC4	HPR	0.733
APOC4	HP	0.733
GAD2	AMPH	0.733
ETNK2	GYS2	0.733
SAA2	HP	0.733
MUC6	TFF1	0.733
PLA2G2A	CYP2B6	0.732
GPX3	GGT6	0.732
IGF1R	EREG	0.732
APOF	SAA4	0.732
HTR3A	CACNG4	0.731
SAA1	APOC3	0.731
CFAP221	SPAG17	0.73
TGFA	IGF1R	0.73
TNNI3	NKX2-5	0.729
CRYAB	GJA3	0.729
NR1I2	CYP24A1	0.729
PNLIP	MOGAT2	0.728
SAA1	LCAT	0.727
SLC51A	ZDHHC19	0.727
ITPR3	CALML3	0.727
TRIM17	TRIM67	0.727
CDX2	FGF4	0.727
RBP4	SERPINA12	0.726
GABBR1	GAD2	0.726
SERPINC1	HPX	0.726
SAA1	APOA4	0.726
NR1I3	SULT2A1	0.726
SHISA9	CACNG4	0.726
FETUB	HRG	0.725
ATP1A3	ATP12A	0.724
REN	CMA1	0.724
MATN3	COL9A2	0.723
APOC4	APOF	0.723
FXYD1	ATP12A	0.722
PTHLH	IBSP	0.721
EFNA5	IGF1R	0.721
UGT2B7	FABP1	0.721
APOC1	SAA1	0.721

CYP1A2	UGT2B10	0.721
AFP	DLK1	0.719
OVOL2	GRHL2	0.719
HSD11B1	UGT1A8	0.719
HPR	APOF	0.719
CA5A	THRSP	0.718
ASPG	SERPINC1	0.717
CALCA	CHGA	0.717
VANGL2	FZD7	0.717
PLA2G4F	CYP4F2	0.716
CALCA	UCHL1	0.716
SHOX2	HCN4	0.715
CYP19A1	UGT2B7	0.715
CYP2E1	GPT	0.715
CALCA	TRPA1	0.715
HTR2C	SLC6A2	0.715
C3orf52	TECRL	0.714
CALCA	NTS	0.713
GSTM1	ALDH3B2	0.712
SLC5A1	TREH	0.712
APOA4	HRG	0.711
ASS1	POX2	0.711
CYP17A1	SULT2A1	0.711
ALPI	CHRND	0.711
ENPP7	SGPP2	0.71
GAD2	GRIA2	0.709
SCD5	FA2H	0.709
KCNA1	LGI1	0.708
SLC30A8	MTNR1B	0.708
APOA4	F9	0.707
FGF19	IGF1R	0.707
ZNF469	COL8A2	0.707
SERPINC1	C8A	0.707
ADIPOQ	APOC3	0.707
ATP2C2	DCDC2	0.706
GGT6	ALPI	0.706
APOA4	SAA4	0.706
ESRP1	RAB25	0.706
PROM1	KRT19	0.706
MUC1	AFP	0.706
SAA4	PON1	0.706
PROM1	FUT4	0.705
AFP	KLK3	0.705
GABRP	GABRA5	0.704
HSD17B6	UGT2B7	0.704
GABRP	GABRA2	0.703

HSD11B1	UGT1A10	0.703
ADIPOQ	SERPINA12	0.703
APOF	HP	0.703
UGT2B7	ADH4	0.703
PITX2	HCN4	0.703
MUC6	TFF2	0.703
NR1I2	ABCB11	0.702
HMGA2	IGF2BP3	0.702
IDO2	KMO	0.702
PAX3	EYA1	0.702
GABRP	GABRA3	0.701
ITGB6	IBSP	0.701
APOA5	SAA4	0.701
ADIPOQ	GPT	0.701
HP	SAA4	0.701
CCL26	ATP12A	0.7
PFKFB3	PKM	0.7
TAC1	UCHL1	0.7
UGT2B7	CYP26A1	0.7
CRHR1	TAC1	0.7
BMP7	DLX5	0.7
MT1M	MT1E	0.7

Supplementary Figure S4. Hub genes identified by the Degree, Closeness and Betweenne

node_name	MCC	DMNC	MNC	Degree	EPC	BottleNeck	EcCentricity
MT1M	1	0	1	1	2.64	1	0.00459
CCL26	1	0	1	1	27.365	1	0.06271
PAX3	1	0	1	1	1.78	1	0.0034
KMO	1	0	1	1	39.328	1	0.06271
IGF2BP3	1	0	1	1	1.361	1	0.00408
HMGA2	1	0	1	1	1.361	1	0.00408
GABRP	6	0.46346	3	3	3.526	1	0.00612
KLK3	1	0	1	1	62.483	1	0.06898
PROM1	2	0	1	2	43.224	4	0.06271
ATP2C2	1	0	1	1	1.547	1	0.00306
ZNF469	1	0	1	1	37.256	1	0.05306
FA2H	1	0	1	1	1.377	1	0.00408
SCD5	1	0	1	1	1.377	1	0.00408
POX2	1	0	1	1	48.64	1	0.06271
SLC5A1	1	0	1	1	12.069	1	0.04927
TECRL	1	0	1	1	1.411	1	0.00408
C3orf52	1	0	1	1	1.411	1	0.00408
SLC6A2	1	0	1	1	66.209	1	0.06271
TRPA1	1	0	1	1	61.81	1	0.06271
SHOX2	1	0	1	1	6.798	1	0.05306
PLA2G4F	1	0	1	1	41.472	1	0.06898
VANGL2	1	0	1	1	3.834	1	0.00612
ASPG	1	0	1	1	68.896	1	0.06271
THRSP	1	0	1	1	9.629	1	0.05306
OVOL2	1	0	1	1	1.627	1	0.00306
IBSP	2	0	1	2	87.826	4	0.06271
CMA1	1	0	1	1	1.363	1	0.00408
REN	1	0	1	1	1.363	1	0.00408
SERPINA12	2	0.30779	2	2	66.741	1	0.06271
TRIM67	1	0	1	1	2.025	1	0.00459
ZDHHC19	1	0	1	1	1.392	1	0.00408
SLC51A	1	0	1	1	1.392	1	0.00408
MOGAT2	1	0	1	1	23.589	1	0.05748
CRYAB	1	0	1	1	1.542	1	0.00306
PLA2G2A	1	0	1	1	60.851	1	0.06898
SAA2	1	0	1	1	62.987	1	0.06271
GYS2	1	0	1	1	1.536	1	0.00306
FRAS1	1	0	1	1	34.323	1	0.07664
GALP	1	0	1	1	62.305	1	0.06271
BPIFA1	1	0	1	1	62.79	1	0.06271
BPIFA2	1	0	1	1	19.125	1	0.05748
OTOG	1	0	1	1	1.534	1	0.00306
PKHD1	1	0	1	1	5.457	1	0.04927
GAGE2A	1	0	1	1	1.709	1	0.00408

DMRT2	1	0	1	1	40.213	1	0.06271
DZIP1L	1	0	1	1	1.539	1	0.00306
BPIFB1	2	0	1	2	61.05	2	0.06271
TMEM252	1	0	1	1	42.983	1	0.06271
ANO2	1	0	1	1	40.876	1	0.05748
CA9	1	0	1	1	19.617	1	0.05748
UCHL1	3	0.30779	2	3	107.726	3	0.06898
SEC14L2	1	0	1	1	45.354	1	0.05748
SPINT2	1	0	1	1	1.397	1	0.00408
C19orf33	1	0	1	1	1.397	1	0.00408
AGR2	1	0	1	1	44.97	1	0.06271
SCGB1A1	1	0	1	1	1.758	1	0.00408
KCNA1	2	0	1	2	47.357	2	0.06271
DNER	2	0.30779	2	2	62.018	1	0.06271
FOXD1	1	0	1	1	1.654	1	0.0034
ODAM	1	0	1	1	66.478	1	0.06271
PAX9	1	0	1	1	3.247	1	0.04599
MT1A	2	0.30779	2	2	3.851	1	0.00459
PCDHA3	1	0	1	1	1.591	1	0.00255
CADPS	1	0	1	1	2.33	1	0.00612
CXCL17	1	0	1	1	66.189	1	0.05748
USH1C	2	0	1	2	1.761	3	0.00612
DCHS2	1	0	1	1	1.545	1	0.00306
PCDHA12	1	0	1	1	1.572	1	0.00255
SLC30A8	2	0	1	2	103.205	10	0.06898
LRRC52	1	0	1	1	1.355	1	0.00408
KCNU1	1	0	1	1	1.355	1	0.00408
ADIPOQ	4	0.30779	2	4	107.248	3	0.06898
MNX1	1	0	1	1	10.28	1	0.04927
HRK	1	0	1	1	1.398	1	0.00408
TFDP3	1	0	1	1	1.398	1	0.00408
CYP24A1	3	0	1	3	54.43	4	0.06271
CA5A	2	0	1	2	20.985	2	0.05748
CFAP221	2	0.30779	2	2	2.224	1	0.00408
GPR123	1	0	1	1	63.99	1	0.05748
SIX3	1	0	1	1	1.775	1	0.0034
PPP1R14C	1	0	1	1	1.375	1	0.00408
PPP1R14D	1	0	1	1	1.375	1	0.00408
BHLHE41	1	0	1	1	1.376	1	0.00408
RORB	1	0	1	1	1.376	1	0.00408
ASPDH	1	0	1	1	43.231	1	0.06271
GPX3	2	0	1	2	79.941	1	0.07664
CYP8B1	2	0.30779	2	2	34.581	1	0.05748
CTNND2	2	0	1	2	4.233	1	0.00918
NHLH2	1	0	1	1	1.404	1	0.00408
BEX1	1	0	1	1	1.404	1	0.00408

SGCZ	1	0	1	1	1.378	1	0.00408
SNTG1	1	0	1	1	1.378	1	0.00408
FOXJ1	3	0	1	3	2.429	6	0.00612
MCIDAS	1	0	1	1	1.728	1	0.00408
RNF17	1	0	1	1	1.977	1	0.0051
KAAG1	1	0	1	1	1.505	1	0.00306
DCDC2	2	0	1	2	1.762	3	0.00612
CD300LG	1	0	1	1	1.385	1	0.00408
SHD	1	0	1	1	1.385	1	0.00408
PDGFD	1	0	1	1	31.973	1	0.06271
SAA4	40320	0.8164	8	8	167.442	1	0.06898
GJA3	2	0	1	2	1.764	3	0.00612
MIP	1	0	1	1	1.532	1	0.00306
HCN4	4	0	1	4	16.893	7	0.05748
HCN1	1	0	1	1	7.272	1	0.05306
MT1E	25	0.56839	4	5	4.956	2	0.00612
PCSK1N	1	0	1	1	62.682	1	0.06271
GLYAT	1	0	1	1	2.685	1	0.00544
DLK1	2	0	1	2	64.863	2	0.06898
RTL1	1	0	1	1	25.892	1	0.06271
MT1X	26	0.45378	5	5	5.093	1	0.00612
PCDHA6	2	0	1	2	1.933	2	0.0034
HAVCR1	1	0	1	1	61.857	1	0.06271
SLC26A9	1	0	1	1	42.388	1	0.05748
SYT13	1	0	1	1	46.852	1	0.06898
MEP1A	2	0	1	2	49.056	2	0.06898
GPT	3	0	1	3	112.877	13	0.07664
CD1A	1	0	1	1	1.395	1	0.00408
CD207	1	0	1	1	1.395	1	0.00408
CDX2	5	0.30779	2	5	98.546	4	0.06271
GRHL2	2	0	1	2	2.077	2	0.00408
ESRP1	2	0	1	2	2.308	6	0.00612
CALB2	2	0.30779	2	2	97.262	1	0.06898
ATP12A	4	0.30779	2	4	67.49	5	0.06898
CTTNBP2	1	0	1	1	57.188	1	0.06898
SHISA9	24	0.56839	4	4	132.097	1	0.07664
FETUB	6	0.46346	3	3	120.637	1	0.06898
CPA1	4	0	1	4	62.123	8	0.05748
CPA2	1	0	1	1	23.093	1	0.05306
RBPJL	1	0	1	1	10.664	1	0.04927
PTF1A	3	0	1	3	25.62	3	0.05306
GPR50	1	0	1	1	68.108	1	0.05748
HTR3A	27	0.56839	4	7	149.712	6	0.07664
NTRK2	3	0	1	3	55.155	12	0.06898
UGT2B10	1440	0.73175	7	7	152.367	1	0.07664
DPYSL5	6	0.46346	3	3	77.339	1	0.06271

PNMA2	8	0.37893	4	4	79.204	1	0.06271
PCDHA10	2	0	1	2	2.03	5	0.0051
PCDHA11	2	0	1	2	1.93	2	0.0034
PROZ	1	0	1	1	67.613	1	0.06271
MAGEA6	2	0.30779	2	2	2.131	1	0.00408
KCNQ1	1	0	1	1	23.399	1	0.05748
CST4	1	0	1	1	1.398	1	0.00408
CST1	1	0	1	1	1.398	1	0.00408
APOF	4.00E+07	0.90737	12	12	169.686	1	0.06898
CHGA	6	0.46346	3	3	126.893	1	0.06898
SFRP5	24	0.56839	4	4	7.395	1	0.00816
HPX	3	0.30779	2	3	124.255	1	0.06898
MCEMP1	1	0	1	1	46.677	1	0.06898
MT2A	25	0.56839	4	5	4.965	3	0.00918
TRIM17	2	0	1	2	3.143	2	0.00612
HRG	9	0.37893	4	5	133.157	2	0.06898
TGFB2	1	0	1	1	49.812	1	0.06271
OR52E8	1	0	1	1	1.645	1	0.00408
KCNJ16	1	0	1	1	66.337	1	0.06271
CASP14	1	0	1	1	1.787	1	0.00408
CALML3	2	0	1	2	25.536	2	0.06271
MYO10	1	0	1	1	10.432	1	0.05748
EPO	1	0	1	1	1.378	1	0.00408
HAMP	1	0	1	1	1.378	1	0.00408
OR7D2	1	0	1	1	1.633	1	0.00408
B3GNT7	24	0.56839	4	4	117.779	1	0.06271
RTP3	3	0	1	3	2.115	4	0.00816
OR13A1	1	0	1	1	1.643	1	0.00408
AMBN	40321	0.8164	8	9	165.91	2	0.06898
FOLR1	3	0.30779	2	3	56.43	3	0.06898
PI3	3	0.30779	2	3	2.366	2	0.00816
SI	1	0	1	1	1.367	1	0.00408
LOC93432	1	0	1	1	1.367	1	0.00408
UPP2	1	0	1	1	59.09	1	0.06898
GP2	3628800	0.89787	10	10	56.176	1	0.05748
FBN2	1	0	1	1	47.73	1	0.05748
SPAG17	2	0.30779	2	2	2.208	1	0.00408
SPAG6	3	0.30779	2	3	2.537	6	0.00612
UBE2U	2	0.30779	2	2	1.953	1	0.00612
ASB11	2	0.30779	2	2	1.948	1	0.00612
RNF182	2	0.30779	2	2	1.953	1	0.00612
ENTPD3	1	0	1	1	21.397	1	0.04927
ENPP7	6	0.46346	3	3	4.448	1	0.00544
GNMT	2	0	1	2	6.296	3	0.05306
CNTN5	3628800	0.89787	10	10	56.458	1	0.05748
TMEM132A	40320	0.8164	8	8	165.712	1	0.06898

ETNPPL	1	0	1	1	1.529	1	0.00306
ETNK2	2	0	1	2	1.755	3	0.00612
ARHGAP40	1	0	1	1	1.679	1	0.00408
PRSS1	27	0.56839	4	7	126.072	13	0.05748
CHRND	3	0	1	3	32.632	3	0.05748
CHRNA4	1	0	1	1	12.538	1	0.05306
CFTR	27	0.56839	4	7	108.216	6	0.06271
FUT4	2	0	1	2	18.287	3	0.05748
ART3	3628800	0.89787	10	10	56.228	1	0.05748
TNFAIP6	24	0.56839	4	4	136.027	1	0.06271
PRSS21	3628800	0.89787	10	10	55.571	1	0.05748
CEACAM7	3628800	0.89787	10	10	55.663	1	0.05748
AOC1	120	0.64826	5	5	143.7	1	0.06271
FXYD3	1	0	1	1	12.946	1	0.05748
SLC10A1	3	0.30779	2	3	45.508	3	0.05748
ABCB11	3	0.30779	2	3	59.52	3	0.06271
MATN3	40322	0.8164	8	10	167.969	9	0.06898
P2RY4	1.31E+12	1.05156	15	15	170.241	1	0.06271
COL28A1	24	0.56839	4	4	98.343	1	0.05748
MT1H	26	0.45378	5	5	5.019	2	0.00612
MT1G	24	0.56839	4	4	4.876	1	0.00612
MUSK	2	0	1	2	36.529	3	0.05306
PAK7	3	0.30779	2	3	74.482	2	0.05748
CNDP1	5	0.30898	3	4	110.026	2	0.06898
CHST4	24	0.56839	4	4	111.823	1	0.06271
ATP1A3	3	0.30779	2	3	35.738	2	0.06271
FXYD1	2	0.30779	2	2	33.403	1	0.06271
CACNG4	25	0.56839	4	5	136.222	1	0.07664
LGI1	8	0.46346	3	5	99.402	5	0.06898
SERPINB3	2	0.30779	2	2	96.41	1	0.06898
MTNR1B	1.31E+12	1.05156	15	18	170.241	15	0.06271
GGT6	4	0.30779	2	4	100.647	16	0.06898
CTNNA2	2	0	1	2	4.233	1	0.00918
PROK1	1.31E+12	1.05156	15	15	170.109	1	0.06898
KCNJ4	2	0	1	2	62.641	2	0.06271
SEMA3E	2	0	1	2	17.478	3	0.05748
SEMA3C	2	0	1	2	20.716	1	0.05748
PLXNB3	3	0	1	3	13.285	2	0.05306
TFF2	3	0.30779	2	3	104.069	13	0.07664
TNC	40321	0.8164	8	9	167.034	12	0.06898
GPLD1	3628800	0.89787	10	10	56.791	1	0.05748
NTNG1	3628800	0.89787	10	10	56.188	1	0.05748
FOXL2	8	0.46346	3	5	99.893	9	0.06898
ABCB4	2	0	1	2	71.2	4	0.06271
PPFIA4	1	0	1	1	46.945	1	0.06898
ALPI	3628802	0.89787	10	12	56.635	14	0.06271

XPNPEP2	3628801	0.89787	10	11	56.239	5	0.06271
CSAG1	2	0.30779	2	2	2.101	1	0.00408
MAGEA12	3	0.30779	2	3	2.299	2	0.00816
SCN1A	3	0.30779	2	3	43.533	1	0.06898
GPR27	40320	0.8164	8	8	167.442	1	0.06271
NKX2-5	3	0	1	3	14.745	4	0.05306
LY6K	3628800	0.89787	10	10	56.486	1	0.05748
LYPD6B	3628800	0.89787	10	10	56.413	1	0.05748
LPAR2	2.62E+12	0.79641	27	27	170.241	5	0.06898
LDHD	2	0	1	2	57.742	6	0.05748
NKX3-2	7	0.46346	3	4	6.563	2	0.04927
GIPR	40320	0.8164	8	8	167.668	1	0.06271
CDH17	721	0.71324	6	7	7.016	2	0.00918
MMP7	30	0.51861	5	5	112.307	1	0.05748
CERS1	1	0	1	1	2.833	1	0.00544
LPAR3	2.62E+12	0.79641	27	27	170.241	1	0.06898
MMP13	34	0.36588	7	7	121.632	2	0.06271
ITPR3	3	0	1	3	65.538	5	0.06898
KRT12	120	0.64826	5	5	92.812	1	0.06271
PFKFB1	2	0.30779	2	2	27.074	1	0.04599
PFKFB3	4	0.30898	3	3	37.927	2	0.04927
ARHGEF38	1	0	1	1	1.656	1	0.00408
FGF4	2	0	1	2	44.714	1	0.05748
FGF19	2	0	1	2	42.914	2	0.06271
CYP26B1	720	0.71324	6	6	149.008	1	0.06898
REG1A	2	0	1	2	1.906	2	0.00408
REG1B	1	0	1	1	1.594	1	0.00272
HSD11B1	5040	0.76834	7	7	151.731	1	0.07664
DLX6	6	0.46346	3	3	6.862	1	0.04927
KIF3C	1	0	1	1	1.381	1	0.00408
KLC3	1	0	1	1	1.381	1	0.00408
RAB25	3	0.30779	2	3	2.527	6	0.00408
RAB36	2	0.30779	2	2	2.257	1	0.00306
RAB19	2	0.30779	2	2	2.25	1	0.00306
RXRG	1	0	1	1	20.97	1	0.06271
CRABP2	2	0	1	2	55.08	2	0.06898
REG3G	1	0	1	1	1.624	1	0.00272
REG3A	2	0	1	2	1.918	2	0.00408
ADCY1	1.31E+12	0.6731	23	23	170.241	5	0.06271
TCN1	145	0.61814	6	7	154.343	12	0.06271
RHOV	3	0	1	3	2.101	4	0.00816
ARHGAP36	1	0	1	1	1.588	1	0.00408
GRM3	1.31E+12	0.89859	17	17	170.241	1	0.06898
SAA1	2.62E+12	0.79641	27	36	170.241	89	0.06898
AVPR1A	1.31E+12	1.05156	15	15	170.241	1	0.06898
NLGN1	2	0.30779	2	2	102.405	1	0.07664

CLDN18	2	0	1	2	1.77	3	0.00612
CLDN10	1	0	1	1	1.567	1	0.00306
GALNT5	24	0.56839	4	4	111.91	1	0.06271
KRT20	121	0.64826	5	6	95.585	4	0.06271
COL11A1	24	0.56839	4	4	98.908	1	0.05748
GRM1	1.31E+12	0.71851	20	20	170.241	7	0.07664
GLS2	5	0.30898	3	4	109.48	2	0.06898
GCGR	1.31E+12	0.68762	23	23	170.241	4	0.06898
CDH6	720	0.71324	6	6	6.877	1	0.00918
GALNTL6	24	0.56839	4	4	110.175	1	0.06271
KRT19	123	0.64826	5	8	111.655	13	0.06898
KRT80	120	0.64826	5	5	90.817	1	0.06271
CRHR1	40326	0.71599	9	9	168.108	1	0.06271
CDH12	720	0.71324	6	6	6.908	1	0.00918
CDH18	720	0.71324	6	6	6.957	1	0.00918
OLFM4	144	0.61814	6	6	150.283	1	0.06271
CRISP3	144	0.61814	6	6	150.175	1	0.06271
CDH15	721	0.71324	6	7	7.031	2	0.00918
CYP2A13	12242	0.6293	12	12	156.098	2	0.07664
KRT15	120	0.64826	5	5	93.987	1	0.06271
KRT4	120	0.64826	5	5	91.252	1	0.06271
CDH9	720	0.71324	6	6	6.958	1	0.00918
CDH10	720	0.71324	6	6	6.857	1	0.00918
HTR2C	1.31E+12	0.95127	16	18	170.057	3	0.06898
TKTL1	8	0.37893	4	4	54.124	6	0.05306
CHL1	2	0	1	2	38.288	1	0.06898
CYP4F2	7	0.46346	3	4	115.348	2	0.07664
CYP2J2	30	0.51861	5	5	139.907	1	0.07664
SYT9	26	0.45378	5	5	104.166	2	0.06898
CYP2A7	2	0.30779	2	2	98.228	1	0.06898
GALNT3	24	0.56839	4	4	110.719	1	0.06271
OXTR	1.31E+12	1.05156	15	15	169.917	1	0.06898
HPR	3.99E+07	0.81956	12	12	169.216	2	0.06898
PITX2	2	0	1	2	28.462	8	0.06271
SDS	6	0.2842	4	4	16.608	3	0.05748
DUOXA1	1	0	1	1	1.508	1	0.00306
DKK4	2	0.30779	2	2	5.619	1	0.00816
TGFA	8	0.2842	4	6	85.234	5	0.06898
TPH1	2	0	1	2	85.314	1	0.06898
IDO2	4	0.30779	2	4	103.232	2	0.06898
FUT3	2	0	1	2	7.28	2	0.05306
B3GALT5	1	0	1	1	3.108	1	0.04927
PON1	4.00E+07	0.88134	13	13	169.896	2	0.06898
GCK	6	0.30779	2	6	76.439	12	0.05748
TREH	2	0	1	2	29.079	2	0.05306
GLP1R	40320	0.8164	8	8	165.232	1	0.06271

NEFL	2	0.30779	2	2	98.754	1	0.07664
SULT2A1	14	0.25611	7	7	151.127	6	0.06898
CDO1	2	0	1	2	65.21	10	0.06898
GABBR1	1.31E+12	0.89859	17	19	170.057	15	0.06898
CYP2B6	8803	0.48572	17	18	156.889	3	0.07664
CCL16	1.31E+12	1.05156	15	15	170.241	1	0.06271
ENSG000002	1	0	1	1	1.361	1	0.00408
TUBA3C	1	0	1	1	1.361	1	0.00408
PCK1	4	0.30898	3	3	47.628	1	0.05306
UGT8	13	0.47366	4	5	4.884	2	0.00816
FCN2	1	0	1	1	27.907	1	0.05306
MASP2	3	0.30779	2	3	60.472	2	0.05748
BCAT1	2	0.30779	2	2	15.684	1	0.05748
GRIN2A	30	0.32929	7	7	152.035	2	0.07664
DLX5	7	0.46346	3	4	9.409	5	0.05306
MSX2	6	0.46346	3	3	5.952	1	0.04927
BPIFB2	40322	0.8164	8	10	167.362	4	0.06898
DAO	2	0	1	2	3.347	2	0.04927
HAO2	1	0	1	1	1.892	1	0.04599
SCTR	40321	0.8164	8	9	166.849	2	0.06271
CYP26A1	1444	0.6123	8	10	153.293	4	0.07664
RDH16	1	0	1	1	61.666	1	0.06898
ALDH3B2	8	0.46346	3	5	138.657	14	0.07664
ITGB6	33	0.51861	5	8	115.59	11	0.06271
CXCL6	1.31E+12	1.05156	15	15	170.069	1	0.06271
APOC4	3.99E+07	0.93324	11	11	169.742	1	0.06898
KISS1R	1.31E+12	1.05156	15	15	170.241	1	0.06898
GNRH2	1.31E+12	1.05156	15	15	170.064	1	0.06898
A4GNT	24	0.56839	4	4	115.096	1	0.06271
CYP2A6	20907	0.58332	16	17	156.521	3	0.07664
PCK2	4	0.30898	3	3	47.53	1	0.05306
PKM	12	0.37893	4	8	57.483	5	0.05306
CXCL5	1.31E+12	1.05156	15	15	170.096	1	0.06271
CCL25	1.31E+12	1.05156	15	16	169.875	2	0.06271
LAMA1	27	0.45378	5	6	103.498	4	0.06271
C6	6	0.46346	3	3	87.015	1	0.06271
FABP1	30	0.56839	4	9	160.26	22	0.06898
LAMC2	32	0.45378	5	8	117.394	3	0.06271
ADH4	10	0.32413	5	5	141.032	5	0.08622
C9	10	0.32413	5	5	119.515	6	0.06898
TFAP2A	3	0.30779	2	3	66.803	9	0.06898
GABRA2	12	0.47366	4	4	3.822	1	0.00612
GABRA3	13	0.47366	4	5	3.947	3	0.01224
BHMT	5	0.30898	3	4	14.509	4	0.05748
CTH	5	0.30898	3	4	26.917	9	0.06271
L1CAM	4	0.30779	2	4	61.412	3	0.06898

CNTN1	1	0	1	1	25.288	1	0.06271
F12	2	0.30779	2	2	93.018	1	0.06898
NR1I2	10	0.37893	4	6	134.188	8	0.06898
ITGB8	6	0.46346	3	3	86.054	1	0.05748
NMU	2.62E+12	0.79641	27	27	170.241	2	0.06898
ACER1	18	0.45378	5	5	4.935	1	0.00816
GSTM1	1471	0.51877	10	11	155.324	7	0.08622
NR0B1	6	0.46346	3	3	100.37	1	0.06898
DEGS2	13	0.47366	4	5	4.89	3	0.00816
APOC1	4.00E+07	0.88134	13	13	169.894	2	0.06898
RBP2	4	0.30779	2	4	118.306	9	0.06898
CYP2E1	19647	0.46905	19	20	156.698	15	0.08622
NR1I3	122	0.52304	6	6	148.581	1	0.07664
CPLX2	1	0	1	1	43.226	1	0.06898
COL9A2	30	0.32929	7	7	124.23	7	0.06271
SNAP25	9	0.30779	2	9	122.111	13	0.07664
GAD2	16	0.2842	4	13	160.601	87	0.07664
ARNT2	1	0	1	1	58.735	1	0.06898
CYP1A1	21015	0.49129	20	21	156.859	18	0.07664
TERT	1	0	1	1	46.493	1	0.06898
SH3GL3	24	0.56839	4	4	97.053	1	0.06271
WIF1	144	0.61814	6	6	8.032	1	0.00816
LCAT	4.00E+07	0.90737	12	12	169.833	1	0.06898
UGT2B7	2190	0.48295	12	12	156.25	17	0.07664
CYP3A4	21150	0.50971	20	20	156.685	1	0.07664
SIX2	2	0	1	2	2.08	2	0.0051
EYA1	3	0	1	3	2.303	5	0.0051
COL2A1	28	0.38039	6	6	118.708	1	0.06271
COL8A2	25	0.56839	4	5	97.606	2	0.05748
ITGA3	30	0.51861	5	5	100.008	1	0.05748
ITGB4	30	0.51861	5	5	102.186	1	0.05748
NAT2	24	0.56839	4	4	133.555	1	0.07664
CYP1A2	16034	0.50686	18	18	156.554	3	0.07664
WNT7B	168	0.5854	7	7	8.155	1	0.00816
GALNT4	24	0.56839	4	4	113.296	1	0.06271
ALDOB	8	0.37893	4	4	51.557	1	0.05306
PFKP	10	0.32413	5	5	46.766	2	0.04927
ARG1	126	0.43905	7	9	158.548	17	0.06898
HP	4.00E+07	0.44914	22	24	170.187	15	0.06898
ANK1	8	0.2842	4	6	63.198	8	0.07664
SPTA1	6	0.2842	4	4	52.478	1	0.06898
FZD7	25	0.56839	4	5	7.534	2	0.00816
WNT9A	168	0.5854	7	7	8.079	1	0.00816
CALCA	40605	0.45768	16	17	170.109	6	0.06898
PTHLH	40321	0.8164	8	9	166.653	5	0.06271
GAL	1.31E+12	0.88149	18	19	170.241	2	0.06898

AMPH	33	0.37893	4	9	117.393	11	0.06898
SNAP91	26	0.45378	5	5	107.976	1	0.06898
TDRD5	6	0.46346	3	3	2.915	1	0.0051
MUC5B	168	0.47886	10	10	130.808	1	0.06271
EREG	3	0.30779	2	3	53.434	1	0.06271
GABRB3	6	0.46346	3	3	3.525	1	0.00612
GABRA5	12	0.47366	4	4	3.8	1	0.00612
LPA	4.00E+07	0.7545	14	14	170.004	8	0.06898
SPHK1	6	0.46346	3	3	4.369	1	0.00544
SGPP2	18	0.45378	5	5	4.98	2	0.00816
RSPO4	1	0	1	1	2.429	1	0.00612
ZNRF3	2	0	1	2	4.182	2	0.00816
FST	2	0	1	2	42.805	7	0.06271
BMP7	3	0	1	3	22.376	6	0.05748
GRIA2	33	0.32929	7	10	159.457	11	0.08622
GRIP1	3	0	1	3	91.18	2	0.08622
SFRP2	120	0.64826	5	5	7.735	1	0.00816
MUC6	174	0.34487	13	13	132.808	13	0.06898
CYP19A1	78	0.57059	6	9	151.364	11	0.06898
CYP17A1	54	0.47564	7	7	149.795	1	0.07664
HSD17B6	102	0.49567	8	8	154.359	1	0.06898
AMDHD1	1	0	1	1	1.389	1	0.00408
UROC1	1	0	1	1	1.389	1	0.00408
ERBB4	4	0.30779	2	4	72.889	3	0.06898
NRG3	2	0.30779	2	2	42.938	1	0.06271
TFF1	6	0.30898	3	5	114.248	3	0.06898
MMP10	24	0.56839	4	4	103.965	1	0.05748
MMP3	32	0.42794	6	6	118.149	1	0.06271
TDRD1	6	0.46346	3	3	2.871	1	0.0051
NTS	1.31E+12	0.91087	18	19	170.241	16	0.07664
TAC1	1.31E+12	0.7235	21	21	170.241	6	0.06898
MUC1	171	0.47886	10	13	134.924	47	0.06898
MUC5AC	172	0.38051	12	12	131.981	1	0.06271
GRP	1.31E+12	0.94716	17	17	170.241	1	0.06898
GAST	1.31E+12	0.87415	18	20	170.241	20	0.07664
AFP	40328	0.69213	9	15	169.145	85	0.07664
IGF1R	5	0.30779	2	5	77.183	13	0.06898
ESR1	16	0.2842	4	11	126.961	47	0.07664
PKP3	2	0.30779	2	2	2.155	1	0.00408
DSC3	2	0.30779	2	2	2.128	1	0.00408
APOA4	4.00E+07	0.54276	19	19	170.357	12	0.07664
APOA5	4.00E+07	0.58955	20	20	170.357	2	0.06898
PIWIL4	7	0.46346	3	4	3.043	2	0.0102
TDRD9	6	0.46346	3	3	2.918	1	0.0051
EPHB6	120	0.64826	5	5	36.338	1	0.05748
WNT3A	171	0.49567	8	9	8.288	5	0.01224

FZD1	146	0.51223	7	7	7.996	1	0.00816
EPHA6	120	0.64826	5	5	36.543	1	0.05748
TNNT1	2	0.30779	2	2	5.306	1	0.04599
DUOXA2	1	0	1	1	1.5	1	0.00306
DUOX2	2	0	1	2	1.722	3	0.00612
HPD	1	0	1	1	6.859	1	0.04927
TAT	3	0.30779	2	3	11.438	2	0.05306
SST	1.31E+12	0.6731	23	23	170.241	6	0.06898
SSTR3	1.31E+12	1.05156	15	15	170.241	1	0.06271
CPS1	3	0.30779	2	3	94.547	1	0.06271
UGT1A8	15294	0.57479	13	13	156.889	1	0.07664
UGT1A10	15144	0.71829	10	10	155.79	1	0.07664
HBA2	2	0.30779	2	2	88.87	1	0.06271
HBA1	4	0.30898	3	3	111.453	1	0.06271
APOC3	4.00E+07	0.76113	15	16	170.182	16	0.07664
APOA2	4.00E+07	0.5133	23	23	170.357	8	0.07664
CLPS	1	0	1	1	24.936	1	0.05748
PNLIP	4	0	1	4	59.661	8	0.06271
EPHB3	120	0.64826	5	5	36.485	1	0.05748
C8A	10	0.32413	5	5	106.334	3	0.06271
C8B	8	0.37893	4	4	88.772	1	0.06271
EPHA7	120	0.64826	5	5	36.701	1	0.05748
EFNA5	124	0.64826	5	9	46.172	10	0.06271
EPHA5	120	0.64826	5	5	34.386	1	0.05748
TTR	9	0.25931	5	6	149.96	4	0.07664
RBP4	4	0.30779	2	4	103.924	1	0.06898
TNNC1	2	0.30779	2	2	5.199	1	0.04599
TNNI3	3	0.30779	2	3	7.034	3	0.04927
F9	5	0.30898	3	4	122.223	2	0.06898
SERPINC1	40458	0.33056	18	20	170.204	14	0.06898
OTC	7	0.30898	3	6	114.482	9	0.06271
ASS1	7	0.30898	3	6	118.104	3	0.06898

ess in the PPI network.

Closeness	Radiality	Betweenness	Stress	ClusteringCoefficient
3.91667	0.05969	0	0	0
62.46995	6.94913	0	0	0
2.33333	0.03061	0	0	0
63.10718	7.0003	0	0	0
1	0.01224	0	0	0
1	0.01224	0	0	0
4	0.02694	0	0	1
81.50198	7.84157	0	0	0
70.83694	7.38511	2004	4866	0
1.5	0.01837	0	0	0
53.40416	6.29823	0	0	0
1	0.01224	0	0	0
1	0.01224	0	0	0
69.0767	7.35441	0	0	0
51.42616	6.07716	0	0	0
1	0.01224	0	0	0
1	0.01224	0	0	0
74.69217	7.4936	0	0	0
72.69535	7.43424	0	0	0
46.25266	5.57773	0	0	0
59.30119	6.74854	0	0	0
4.91667	0.07124	0	0	0
75.09289	7.50793	0	0	0
44.53345	5.43649	0	0	0
2.33333	0.03918	0	0	0
75.18027	7.50383	880.70417	3062	0
1	0.01224	0	0	0
1	0.01224	0	0	0
68.12475	7.21727	0	0	1
3.33333	0.05051	0	0	0
1	0.01224	0	0	0
1	0.01224	0	0	0
58.51374	6.68304	0	0	0
1.5	0.01837	0	0	0
65.69167	7.02896	0	0	0
78.1767	7.60618	0	0	0
1.5	0.01837	0	0	0
67.47302	7.35032	0	0	0
74.97987	7.51407	0	0	0
72.48694	7.44857	0	0	0
59.14066	6.76491	0	0	0
1.5	0.01837	0	0	0
43.12753	5.22976	0	0	0
2	0.01905	0	0	0

62.18896	6.9348	0	0	0
1.5	0.01837	0	0	0
73.15361	7.45266	672	1646	0
65.10328	7.13744	0	0	0
60.86053	6.79971	0	0	0
51.39181	6.12015	0	0	0
82.61905	7.76993	3439.72202	19136	0.33333
64.96169	7.1006	0	0	0
1	0.01224	0	0	0
1	0.01224	0	0	0
65.03059	7.14972	0	0	0
2.66667	0.03184	0	0	0
62.02547	6.87749	288.38215	1158	0
67.12471	7.18862	0	0	1
2.16667	0.02806	0	0	0
72.23694	7.44447	0	0	0
37.73668	4.31481	0	0	0
4.41667	0.06199	0	0	1
2.08333	0.03827	0	0	0
3	0.02204	0	0	0
72.48626	7.36669	0	0	0
2	0.02143	2	2	0
1.5	0.01837	0	0	0
2.08333	0.03827	0	0	0
86.53929	7.95824	539.96793	3560	0
1	0.01224	0	0	0
1	0.01224	0	0	0
85.38492	7.85794	649.08486	2666	0.16667
47.65929	5.72305	0	0	0
1	0.01224	0	0	0
1	0.01224	0	0	0
62.18178	6.8079	2008	8164	0
52.05848	6.12424	672	2724	0
3.16667	0.03429	0	0	1
65.16522	7.01872	0	0	0
2.33333	0.03061	0	0	0
1	0.01224	0	0	0
1	0.01224	0	0	0
1	0.01224	0	0	0
1	0.01224	0	0	0
69.0767	7.35441	0	0	0
76.39603	7.6246	1451.03529	5030	0
55.85602	6.47221	0	0	1
5	0.02985	1	2	0
1	0.01224	0	0	0
1	0.01224	0	0	0

1	0.01224	0	0	0
1	0.01224	0	0	0
4	0.04163	14	14	0
2.66667	0.03184	0	0	0
2.5	0.02041	0	0	0
1.5	0.01837	0	0	0
2	0.02143	2	2	0
1	0.01224	0	0	0
1	0.01224	0	0	0
61.72428	6.90615	0	0	0
89.99563	7.92958	0	0	1
2	0.02143	2	2	0
1.5	0.01837	0	0	0
55.10505	6.26548	2421.56516	12284	0
46.25266	5.57773	0	0	0
6.33333	0.07577	14	16	0.6
84.13185	7.817	0	0	0
3.66667	0.03965	0	0	0
82.16865	7.84566	672	1620	0
64.97027	7.15791	0	0	0
6.33333	0.07577	6	12	0.7
2.83333	0.04592	6	6	0
73.88773	7.5284	0	0	0
60.86053	6.79971	0	0	0
68.62341	7.31347	0	0	0
64.14325	6.91638	1664.21408	5506	0
92.08016	8.12608	2721.31551	10390	0
1	0.01224	0	0	0
1	0.01224	0	0	0
72.75877	7.30529	2542.34911	9704	0.1
3.16667	0.04898	8	8	0
3.5	0.05388	12	12	0
88.4004	8.04216	0	0	1
79.19286	7.63688	2670	8006	0.16667
67.9254	7.27254	0	0	0
78.04127	7.66144	0	0	1
86.2123	7.88865	0	0	1
68.45018	7.09037	2990.33497	8656	0
55.41313	6.40262	0	0	0
47.65929	5.72305	0	0	0
56.74646	6.4108	1342	3918	0
73.51959	7.39535	0	0	0
88.55159	7.96029	1317.10042	7954	0.33333
73.22857	7.43015	3695.26107	19010	0
80.4377	7.63688	0.4	4	0.95238
68.68185	7.22341	0	0	1

69.18185	7.22546	11.53389	42	0.66667
3	0.04847	8	8	0
2.83333	0.04592	6	6	0
75.09289	7.50793	0	0	0
2.5	0.02177	0	0	1
62.08268	6.9389	0	0	0
1	0.01224	0	0	0
1	0.01224	0	0	0
92.32897	7.94391	1.41667	14	0.93939
86.84841	7.92754	0	0	1
7.16667	0.08905	0	0	1
86.13968	7.84361	61.46099	330	0.33333
68.62341	7.31347	0	0	0
6.5	0.07806	24	28	0.6
4.66667	0.06658	14	16	0
87.2123	7.89274	683.99996	1446	0.4
67.31959	7.20499	0	0	0
2	0.01905	0	0	0
77.30884	7.62255	0	0	0
2	0.01905	0	0	0
60.01872	6.79562	672	2078	0
50.37529	6.10787	0	0	0
1	0.01224	0	0	0
1	0.01224	0	0	0
2	0.01905	0	0	0
76.67662	7.61027	0	0	1
3	0.02449	6	6	0
2	0.01905	0	0	0
96.24841	8.13222	672	1646	0.77778
68.49127	7.23979	1453.4673	4366	0.33333
3	0.02449	4	4	0.33333
1	0.01224	0	0	0
1	0.01224	0	0	0
65.04167	7.01054	0	0	0
65.41919	6.79766	0	0	1
64.61804	7.05557	0	0	0
3.16667	0.03429	0	0	1
4	0.04163	12	12	0.33333
2	0.01224	0	0	1
2	0.01224	0	0	1
2	0.01224	0	0	1
51.57021	6.01371	0	0	0
4.83333	0.04665	0	0	1
48.468	5.75376	1340	3952	0
65.41919	6.79766	0	0	1
95.74841	8.13018	0	0	1

1.5	0.01837	0	0	0
2	0.02143	2	2	0
2	0.01905	0	0	0
79.6553	7.49974	4190.58856	14264	0.28571
63.29939	6.85702	1549.35244	4082	0
52.10043	6.16927	0	0	0
77.33979	7.48746	3322.23051	11332	0.28571
58.59888	6.70555	1340	3248	0
65.41919	6.79766	0	0	1
81.09217	7.66554	0	0	1
65.41919	6.79766	0	0	1
65.41919	6.79766	0	0	1
83.85725	7.77402	0	0	1
52.6403	6.26752	0	0	0
62.73936	6.89591	533.07697	2678	0.33333
62.18178	6.8079	437.78637	1918	0.33333
98.13175	8.16497	4398.50971	18416	0.64444
98.19574	8.0524	0	0	1
64.82796	6.98393	0	0	1
6.33333	0.07577	6	12	0.7
5.83333	0.07347	0	0	1
58.82819	6.63187	799.32726	1922	0
63.50061	6.88158	1046.10412	2860	0.33333
82.97381	7.82519	737.48762	2854	0.33333
76.67662	7.61027	0	0	1
63.63662	6.95527	672	2006	0.33333
63.13662	6.95322	0	0	1
80.01508	7.70852	705.02292	4744	0.6
73.34841	7.40354	995.38068	4866	0.3
76.94524	7.69419	0	0	1
100.97908	8.0831	6832.5817	60752	0.68627
85.17857	7.87022	6985.54651	23206	0.16667
5	0.02985	1	2	0
98.88056	8.10152	0	0	1
77.97551	7.62665	672	2350	0
58.68665	6.59707	668	4140	0
58.68665	6.59707	668	4140	0
50.45023	5.91751	673	4146	0
86.61587	7.95824	2928.62227	18532	0.33333
98.17103	8.1793	4300.43447	12644	0.77778
65.41919	6.79766	0	0	1
65.41919	6.79766	0	0	1
78.8627	7.62255	6676.43484	36222	0.3
75.0544	7.54068	1056.45132	5326	0
68.62341	7.31347	0	0	0
76.60289	7.38511	6833.27288	21944	0.68182

68.43066	6.97165	1669.66836	5344	0.81818
2.5	0.02177	0	0	1
3	0.02449	4	4	0.33333
60.40794	6.74444	240.3741	740	0.33333
84.63463	7.70443	0	0	1
50.43639	5.73124	2071	14822	0
65.41919	6.79766	0	0	1
65.41919	6.79766	0	0	1
109.09206	8.25913	859.06764	12028	0.61538
67.86883	7.15382	1905.70806	6168	0
43.79518	5.00256	672	3072	0.5
84.63463	7.70443	0	0	1
7.5	0.04133	11	12	0.71429
69.9434	7.12721	249.61206	1120	0.8
3.66667	0.03965	0	0	0
109.09206	8.25913	859.06764	12028	0.61538
75.44257	7.3933	1176.0542	6210	0.47619
73.86508	7.47927	2737.58251	7812	0
72.59527	7.41582	0	0	1
47.54196	5.5286	0	0	1
52.73688	6.01985	96.17984	938	0.66667
2	0.01905	0	0	0
62.63975	6.88363	425.43846	910	0
64.38222	7.01054	577.99557	1128	0
77.3754	7.48951	0	0	1
2.5	0.03265	4	4	0
1.83333	0.02721	0	0	0
80.27103	7.63483	0	0	1
43.29518	5.00051	0	0	1
1	0.01224	0	0	0
1	0.01224	0	0	0
3.83333	0.05388	12	12	0.33333
3.08333	0.04653	0	0	1
3.08333	0.04653	0	0	1
52.5017	6.24091	0	0	0
63.55	6.92866	672	3730	0
1.83333	0.02721	0	0	0
2.5	0.03265	4	4	0
103.87551	8.12813	2129.77978	11806	0.54941
87.81201	7.85999	2213.56845	8490	0.61905
3	0.02449	6	6	0
2	0.01905	0	0	0
101.19206	8.13836	589.88476	5896	0.81618
122.90278	8.50475	18048.41581	106532	0.3873
98.88056	8.10152	0	0	1
83.70992	7.89069	0	0	1

2	0.02143	2	2	0
1.5	0.01837	0	0	0
76.67662	7.61027	0	0	1
74.75956	7.46494	586.34689	1882	0.66667
64.82796	6.98393	0	0	1
106.77381	8.31644	3240.93259	24872	0.61579
82.2	7.79858	450.55433	1922	0.33333
103.87817	8.14655	1737.42362	10368	0.56126
7	0.03903	0	0	1
76.67662	7.61027	0	0	1
91.64841	8.06058	5963.48023	15830	0.39286
72.59527	7.41582	0	0	1
85.63463	7.71875	27.08052	178	0.83333
7	0.03903	0	0	1
7	0.03903	0	0	1
84.35725	7.77607	18.78413	120	0.86667
84.35725	7.77607	18.78413	120	0.86667
7.5	0.04133	11	12	0.71429
83.50913	7.67986	347.98886	3924	0.65152
72.59527	7.41582	0	0	1
72.59527	7.41582	0	0	1
7	0.03903	0	0	1
7	0.03903	0	0	1
102.65437	8.18135	2377.7233	13818	0.69281
64.68091	6.81404	655.2743	10914	0.66667
65.03571	7.04738	716.69257	1752	0
74.42937	7.43629	672	3256	0.5
77.23611	7.54886	25.4997	236	0.8
75.57937	7.48951	436.22807	1824	0.7
65.54167	7.01258	0	0	1
76.67662	7.61027	0	0	1
98.88056	8.10152	0	0	1
92.32897	7.94391	149.52123	1536	0.84848
62.15642	6.90001	2990.56516	14456	0
56.56887	6.42923	668	3940	0.5
1.5	0.01837	0	0	0
6.16667	0.0846	0	0	1
78.36151	7.5939	2535.02395	7640	0.2
81.24484	7.76174	891.60196	4908	0
80.22619	7.68805	1360.38258	6216	0.16667
50.07166	6.0219	672	1626	0
43.23321	5.33415	0	0	0
102.25913	8.2141	160.13428	2302	0.88462
78.21959	7.44857	5325.62701	51172	0.13333
61.93091	6.76491	672	4296	0
84.63463	7.70443	0	0	1

75.4746	7.62255	0	0	1
85.56389	7.83133	2256.31923	12824	0.33333
79.95119	7.75355	5904	21560	0
107.22183	8.3103	5763.3121	32894	0.64912
87.97579	7.71671	1708.57143	11910	0.39216
98.19574	8.0524	0	0	1
1	0.01224	0	0	0
1	0.01224	0	0	0
64.01425	6.80994	129.28206	2140	0.66667
6	0.05364	13.33333	20	0.5
51.81015	6.15699	0	0	0
62.55335	6.84474	672	4276	0.33333
55.1022	6.41694	0	0	1
89.26468	7.96438	697.03001	6110	0.42857
49.64714	5.68212	2664	12264	0.5
43.29518	5.00051	0	0	1
96.91508	8.13632	2008	4930	0.62222
42.46814	5.0701	672	1978	0
37.31143	4.38235	0	0	0
85.13463	7.70647	672	3030	0.77778
81.59881	7.61232	2028.87715	11380	0.46667
62.88333	6.92457	0	0	0
90.41825	8.05649	5826.12578	26046	0.4
83.3425	7.74332	4965.22457	15200	0.28571
98.19574	8.0524	0	0	1
91.82897	7.94186	0	0	1
98.88056	8.10152	0	0	1
98.88056	8.10152	0	0	1
76.67662	7.61027	0	0	1
86.59246	7.69829	1178.65736	9378	0.47794
64.01425	6.80994	129.28206	2140	0.66667
63.79285	6.70146	1800.4363	10942	0.17857
98.19574	8.0524	0	0	1
98.69574	8.05444	672	4016	0.875
73.35444	7.37488	2213.57409	6948	0.46667
66.99737	7.11288	0	0	1
99.13294	8.21614	11634.86775	80490	0.22222
74.7053	7.35851	1730.01742	6612	0.35714
83.84167	7.87022	720.02522	2924	0.5
84.80873	7.79244	1181.9774	6498	0.5
74.76389	7.53454	3615.56516	16682	0.33333
4.5	0.02939	0.66667	2	0.83333
5	0.03184	8.66667	10	0.5
56.81887	6.43332	2664	9850	0.33333
66.35725	7.09855	5268	19628	0.33333
66.98016	7.11288	1952.07255	5668	0.16667

54.60411	6.42513	0	0	0
77.53016	7.63074	0	0	1
77.11825	7.48336	3582.28201	15472	0.26667
65.61804	7.05966	0	0	1
109.09206	8.25913	859.06764	12028	0.61538
6	0.05364	3.66667	10	0.7
89.11548	7.94596	3056.45584	14526	0.47273
77.22937	7.59799	0	0	1
6	0.05364	13.33333	20	0.5
102.25913	8.2141	160.13428	2302	0.88462
87.82897	7.92958	3668.04913	12230	0.16667
96.87143	8.08515	6052.85901	28356	0.36842
79.4377	7.62869	15.3346	114	0.73333
68.62341	7.31347	0	0	0
80.78892	7.66349	2332.36072	10962	0.42857
89.97063	8.00122	6444.71261	21366	0.05556
107.00238	8.40446	24849.8031	98972	0.07692
72.5254	7.44038	0	0	0
99.31349	8.12813	6701.17789	42676	0.38095
71.85079	7.46904	0	0	0
71.35328	7.31347	0	0	1
8.16667	0.09351	1	4	0.86667
101.75913	8.21205	117.87897	1814	0.93939
93.54921	8.06468	3092.31064	27520	0.5
92.01984	7.84975	2416.58543	11708	0.43684
3	0.03571	6	6	0
3.5	0.03827	10	10	0
80.28892	7.66144	1610.84672	7816	0.53333
65.32796	6.98597	672	2974	0.6
69.9228	7.21318	45.77489	294	0.8
69.9228	7.21318	45.77489	294	0.8
74.85079	7.4629	0	0	1
88.67222	7.76583	654.29401	4908	0.45098
8.83333	0.09796	4.33333	16	0.7619
76.67662	7.61027	0	0	1
64.68091	6.81404	655.2743	10914	0.66667
56.7591	6.2143	635.59416	10410	0.5
92.7996	8.06468	2661.58918	8214	0.36111
110.04246	8.29393	6741.88462	30560	0.31159
71.98889	7.35441	2679.13987	7324	0.2
65.49325	7.05762	503.14078	1928	0.5
7.83333	0.09351	20	38	0.6
8.83333	0.09796	4.33333	16	0.7619
98.78532	8.12199	2864.85364	15406	0.375
86.26797	7.74127	1115.74185	3622	0.77778
103.30873	8.20182	1104.1655	8622	0.70175

86.73095	7.87432	5190.63436	17822	0.27778
75.57937	7.48951	436.22807	1824	0.7
3.5	0.02551	0	0	1
79.67662	7.62255	10.5	42	0.53333
63.41952	6.9348	49.37104	118	0.33333
4	0.02694	0	0	1
4.5	0.02939	0.66667	2	0.83333
103.89246	8.23252	1602.95053	10464	0.73626
4.83333	0.04665	0	0	1
6	0.05364	3.66667	10	0.7
4.41667	0.06456	0	0	0
6.33333	0.08683	20	20	0
64.43896	6.98802	5407.43484	30156	0
56.62529	6.35349	4872.43484	27228	0
98.46786	8.26322	8215.52555	37880	0.22222
86.2381	8.03807	5790.65486	27128	0
7.66667	0.09128	0	0	1
86.68175	7.84771	2119.04213	10766	0.34615
87.82143	7.86408	6739.63886	32992	0.41667
83.17222	7.76379	173.52743	1398	0.61905
80.79881	7.64097	73.71624	344	0.60714
1	0.01224	0	0	0
1	0.01224	0	0	0
74.58294	7.50997	918.2148	4186	0.33333
61.10999	6.85497	0	0	1
83.16706	7.83747	2458.60612	15486	0.2
66.98907	7.01668	0	0	1
74.94257	7.39126	683.02404	3664	0.6
3.5	0.02551	0	0	1
110.74762	8.43311	6246.35733	35742	0.72515
106.20635	8.26527	3546.63509	22330	0.60952
99.22579	8.23866	13344.76202	47566	0.32051
82.12424	7.66758	877.82866	4550	0.39394
102.34206	8.20182	98.29624	1572	0.86029
106.73333	8.30825	6310.11571	31270	0.62632
113.57421	8.52932	19829.25367	73388	0.28571
78.87103	7.61436	4994.77866	15460	0.1
95.78651	8.15679	20023.11837	83392	0.10909
2.5	0.02177	0	0	1
2.5	0.02177	0	0	1
111.29802	8.39422	6061.40114	34300	0.47368
107.18889	8.2796	3300.28402	18844	0.50526
4	0.02806	6	6	0.5
3.5	0.02551	0	0	1
59.76999	6.59707	0	0	1
10	0.10464	48.33333	66	0.47222

8.83333	0.09796	8	20	0.66667
59.76999	6.59707	0	0	1
38.60016	4.36598	0	0	1
1.5	0.01837	0	0	0
2	0.02143	2	2	0
41.91814	5.06601	0	0	0
48.88467	5.75376	672	3940	0.33333
112.32183	8.42083	8788.8524	49480	0.54941
98.19574	8.0524	0	0	1
74.22074	7.52226	239.27963	808	0.33333
87.38889	7.79654	1227.21907	6142	0.57692
81.9377	7.64302	16.73331	178	0.8
78.6767	7.60822	0	0	1
79.1767	7.61027	1	2	0.66667
108.84802	8.35943	3104.07737	25224	0.63333
118.22183	8.53136	10594.35131	55074	0.41897
58.51374	6.68304	0	0	0
72.7307	7.37079	3231.56369	10280	0
59.76999	6.59707	0	0	1
77.84289	7.52635	1596.3359	9544	0.5
67.66403	7.11697	78.60051	916	0.66667
59.76999	6.59707	0	0	1
73.0798	7.27663	6384.13586	24834	0.27778
59.76999	6.59707	0	0	1
95.50754	8.19772	2106.10045	7814	0.26667
77.15516	7.61641	237.83825	920	0.16667
38.60016	4.36598	0	0	1
44.07094	5.05168	1340	9832	0.33333
90.37738	8.04421	1908.26595	6422	0.33333
103.57222	8.19568	6498.01716	28726	0.23684
83.28304	7.78835	3722.52213	11312	0.13333
89.8496	8.04216	3878.89508	11062	0.13333

Supplementary Figure S5. Genes with high degree, closeness, and betweenness scores

Gene	P value	Hazard ratio	Low 95%CI	High 95%CI
ADIPOQ	0.895837	0.876919403	0.122755	6.264399
CDX2	0.595828	1.068172271	0.837156	1.362939
ATP12A	0.63697	0.941970396	0.734876	1.207426
CPA1	0.21386	1.279298939	0.867564	1.886439
HTR3A	0.491367	1.080349391	0.866894	1.346364
UGT2B10	0.108993	0.799531639	0.608156	1.05113
PNMA2	0.004458	1.352930826	1.098496	1.666298
APOF	0.070514	0.788432108	0.60938	1.020094
HRG	0.304102	0.878170731	0.685447	1.125082
AMBN	0.162824	1.160508452	0.941585	1.430333
PRSS1	6.91E-07	1.928234655	1.487871	2.498932
CFTR	0.683232	1.055714248	0.813672	1.369756
MATN3	0.268964	1.119812967	0.916243	1.368612
CNDP1	0.055777	0.696036405	0.480155	1.00898
CACNG4	0.554127	1.076714957	0.842848	1.375474
LGI1	0.30436	1.114573609	0.906183	1.370887
MTNR1B	0.389308	1.134320151	0.85137	1.511309
GGT6	0.006916	1.344988689	1.084702	1.667734
TNC	0.709486	1.051693347	0.806735	1.371032
FOXL2	0.513201	1.066819652	0.878795	1.295073
ALPI	0.095427	1.177343812	0.971765	1.426414
XPNPEP2	0.723304	0.955308609	0.741688	1.230456
LPAR2	0.335067	1.128966575	0.882201	1.444757
MMP7	0.010254	1.336711436	1.071053	1.668262
LPAR3	0.751556	1.04021306	0.814929	1.327776
MMP13	0.742617	1.042708915	0.812378	1.338345
ADCY1	0.788808	1.035650575	0.801485	1.33823
TCN1	0.651595	0.935935499	0.702162	1.24754
GRM3	0.038439	1.208612974	1.010136	1.446088
SAA1	0.182761	0.840622363	0.651149	1.085229
KRT20	0.004128	1.31926959	1.091702	1.594274
GRM1	0.267172	1.088639099	0.936986	1.264837
GLS2	0.003798	0.596142624	0.419983	0.846192
GCGR	0.007513	0.689181831	0.524569	0.905451
KRT19	0.805395	1.030987483	0.808758	1.314282
CRHR1	0.396435	1.084348087	0.899258	1.307534
OLFM4	0.490212	1.062487652	0.894432	1.26212
CRISP3	0.729281	1.045260733	0.813486	1.343072
CYP2A13	0.532202	0.925324813	0.72533	1.180465
HTR2C	0.092067	1.183188869	0.972878	1.438964
CYP4F2	0.029235	0.765676552	0.602294	0.973379
CYP2J2	0.768108	1.041078129	0.796647	1.360506
SYT9	0.420406	0.883493773	0.653642	1.194173
HPR	0.014237	0.735096474	0.574745	0.940185
TGFA	0.07594	1.237825457	0.977979	1.566712
IDO2	0.055325	0.709398984	0.499327	1.00785
PON1	0.013014	0.749061414	0.596325	0.940918
GCK	0.674474	1.053999336	0.824628	1.347171
SULT2A1	0.01009	0.753609951	0.607511	0.934844
GABBR1	0.962873	1.006592261	0.763312	1.327411

CYP2B6	0.01766	0.709355664	0.534157	0.942019
GRIN2A	0.051824	1.224149475	0.998403	1.500939
BPIFB2	0.621131	0.942557911	0.745462	1.191765
SCTR	0.137828	1.190333105	0.94564	1.498342
CYP26A1	0.392444	0.829424275	0.540289	1.27329
ALDH3B2	0.32226	1.156510868	0.867174	1.542386
ITGB6	0.055431	1.306161577	0.993817	1.716673
CYP2A6	0.403928	0.895006465	0.689761	1.161324
CCL25	0.672347	0.947048352	0.735994	1.218624
LAMA1	0.368952	1.0969114	0.896471	1.342167
FABP1	0.375045	0.888018598	0.683063	1.154472
LAMC2	0.0573	1.270268034	0.992609	1.625595
ADH4	0.0001	0.604133132	0.468659	0.778768
C9	0.302101	0.870693751	0.669347	1.132607
CTH	0.043537	0.77106172	0.599037	0.992486
L1CAM	0.129639	0.586457957	0.294109	1.169408
NR1I2	0.022035	0.750785994	0.587438	0.959556
NMU	0.004475	1.266528816	1.076122	1.490626
GSTM1	0.763521	0.955458014	0.710067	1.285653
APOC1	0.18658	0.844194284	0.656577	1.085423
RBP2	0.801335	1.035512212	0.789052	1.358955
CYP2E1	0.122109	0.830051044	0.655462	1.051143
NR1I3	0.028068	0.764648422	0.601819	0.971534
COL9A2	0.038683	1.238315177	1.011184	1.516465
SNAP25	0.580495	1.070782915	0.840172	1.364692
GAD2	0.650643	0.871942371	0.481866	1.57779
CYP1A1	0.746582	0.957253028	0.73443	1.24768
LCAT	1.09E-05	0.549480893	0.420814	0.717488
UGT2B7	0.81578	0.96855675	0.740285	1.267217
CYP3A4	0.038163	0.738111378	0.553912	0.983565
COL2A1	0.971804	1.003789243	0.813882	1.238008
COL8A2	0.407554	1.098395813	0.879635	1.371562
ITGA3	0.040201	1.26599025	1.010599	1.585922
ITGB4	0.356812	1.128707971	0.872447	1.46024
CYP1A2	0.277012	0.831502231	0.596179	1.159711
ARG1	0.131648	0.837101826	0.664337	1.054796
HP	0.08002	0.788551992	0.604394	1.028823
ANK1	0.486769	1.102556055	0.837347	1.451763
SPTA1	0.659586	1.069335589	0.793541	1.440982
CALCA	0.97266	1.004297069	0.785899	1.283388
PTHLH	0.209362	1.167485196	0.916749	1.4868
GAL	0.004287	1.303145262	1.086634	1.562797
AMPH	0.220297	1.140144711	0.924444	1.406175
SNAP91	0.02846	1.268075689	1.025349	1.568262
MUC5B	0.30283	1.115645144	0.90599	1.373816
LPA	0.004778	0.656240729	0.489769	0.879296
GRIA2	0.981758	0.997074316	0.775626	1.281748
MUC6	0.010618	1.311983135	1.065282	1.615815
CYP19A1	0.405708	1.102477814	0.87598	1.387539
CYP17A1	0.554054	0.920163421	0.698505	1.212161
HSD17B6	0.024732	0.765808782	0.606706	0.966634
ERBB4	0.11824	1.118290092	0.971935	1.286683

TFF1	0.346743	1.092690948	0.908462	1.31428
MMP3	0.041443	1.249406727	1.008676	1.54759
NTS	0.30543	1.118527946	0.902846	1.385735
TAC1	0.206691	0.335098574	0.061398	1.828903
MUC1	0.144819	1.15998214	0.9502	1.416079
MUC5AC	0.917401	1.012687579	0.797987	1.285155
GRP	0.100811	1.205204144	0.964362	1.506194
GAST	0.072793	1.181138599	0.98473	1.416722
AFP	0.845555	1.024672678	0.801825	1.309455
IGF1R	0.086591	1.250969955	0.968329	1.61611
ESR1	0.003907	0.583718165	0.404951	0.841403
APOA4	0.312398	1.135774458	0.887189	1.454012
APOA5	0.275742	0.855734549	0.646649	1.132426
SST	0.875845	1.020368961	0.792319	1.314058
UGT1A8	0.011182	1.216404793	1.045563	1.415162
UGT1A10	0.001871	1.327239208	1.110363	1.586476
APOC3	0.075268	0.789536154	0.608546	1.024355
APOA2	0.663499	0.936458632	0.696709	1.258709
PNLIP	0.152089	1.382293617	0.887539	2.152846
C8A	0.032974	0.766692643	0.600583	0.978746
C8B	0.015752	0.708519882	0.53565	0.93718
EFNA5	0.000462	1.437510937	1.173277	1.761253
TTR	0.119478	0.814153587	0.628493	1.054659
RBP4	0.004031	0.747051362	0.612394	0.911318
F9	0.057532	0.789238668	0.618202	1.007596
SERPINC1	0.053976	0.788612695	0.619391	1.004067
OTC	0.005109	0.715556861	0.566107	0.90446
ASS1	0.002311	0.583335265	0.41243	0.825062

Supplementary Table S6. Pathways identified in the TCGA HCC cohort

Tag	Term
DTG	KEGG_DRUG_METABOLISM_CYTOCHROME_P450
DTG.1	KEGG_RETINOL_METABOLISM
DTG.2	KEGG_TRYPTOPHAN_METABOLISM
DTG.3	KEGG_GLYCINE_SERINE_AND_THREONINE_METABOLISM
DTG.4	KEGG_FATTY_ACID_METABOLISM
DTG.5	KEGG_METABOLISM_OF_XENOBIOTICS_BY_CYTOCHROME_P450
DTG.6	KEGG_PRIMARY_BILE_ACID_BIOSYNTHESIS
DTG.7	KEGG_VALINE_LEUCINE_AND_ISOLEUCINE_DEGRADATION
DTG.8	KEGG_COMPLEMENT_AND_COAGULATION_CASCADES
DTG.9	KEGG_PEROXISOME
DTG.10	KEGG_BUTANOATE_METABOLISM
DTG.11	KEGG_PROPANOATE_METABOLISM
DTG.12	KEGG_LINOLEIC_ACID_METABOLISM
DTG.13	KEGG_PPAR_SIGNALING_PATHWAY
DTG.14	KEGG_FOLATE_BIOSYNTHESIS
DTG.15	KEGG_STEROID_HORMONE_BIOSYNTHESIS
DTG.16	KEGG_TYROSINE_METABOLISM
DTG.17	KEGG_BETA_ALANINE_METABOLISM
DTG.18	KEGG_ASCORBATE_AND_ALDARATE_METABOLISM
DTG.19	KEGG_DRUG_METABOLISM_OTHER_ENZYMES
DTG.20	KEGG_LIMONENE_AND_PINENE_DEGRADATION
DTG.21	KEGG_HISTIDINE_METABOLISM
DTG.22	KEGG_ALANINE_ASPARTATE_AND_GLUTAMATE_METABOLISM
DTG.23	KEGG_ARGININE_AND_PROLINE_METABOLISM
DTG.24	KEGG_PYRUVATE_METABOLISM
DTG.25	KEGG_ARACHIDONIC_ACID_METABOLISM
DTG.26	KEGG_GLYOXYLATE_AND_DICARBOXYLATE_METABOLISM
DTG.27	KEGG_PENTOSE_AND_GLUCURONATE_INTERCONVERSIONS
DTG.28	KEGG_STARCH_AND_SUCROSE_METABOLISM
DTG.29	KEGG_PHENYLALANINE_METABOLISM
DTG.30	KEGG_CITRATE_CYCLE_TCA_CYCLE
DTG.31	KEGG_GLYCOLYSIS_GLUCONEOGENESIS
DTG.32	KEGG_OLFACTORY_TRANSDUCTION
DTG.33	KEGG_ALPHA_LINOLENIC_ACID_METABOLISM
DTG.34	KEGG_LYSINE_DEGRADATION
DTG.35	KEGG_PORPHYRIN_AND_CHLOROPHYLL_METABOLISM
DTG.36	KEGG_TAURINE_AND_HYPOTAURINE_METABOLISM
DTG.37	KEGG_ONE_CARBON_POOL_BY_FOLATE
DTG.38	KEGG_BIOSYNTHESIS_OF_UNSATURATED_FATTY_ACIDS
DTG.39	KEGG_ABC_TRANSPORTERS
DTG.40	KEGG_PROXIMAL_TUBULE_BICARBONATE_RECLAMATION
DTG.41	KEGG_CYSTEINE_AND_METHIONINE_METABOLISM
DTG.42	KEGG_PANTOTHENATE_AND_COA_BIOSYNTHESIS
DTG.43	KEGG_NITROGEN_METABOLISM

DTG.44	KEGG_GLUTATHIONE_METABOLISM
DTG.45	KEGG_ASTHMA
DTG.46	KEGG_GLYCEROLIPID_METABOLISM
DTG.47	KEGG_TERPENOID_BACKBONE_BIOSYNTHESIS
DTG.48	KEGG_ADIPOCYTOKINE_SIGNALING_PATHWAY
DTG.49	KEGG_STEROID_BIOSYNTHESIS
DTG.50	KEGG_AUTOIMMUNE_THYROID_DISEASE
DTG.51	KEGG_MATURITY_ONSET_DIABETES_OF_THE_YOUNG
DTG.52	KEGG_RENIN_ANGIOTENSIN_SYSTEM
DTG.53	KEGG_NEUROACTIVE_LIGAND_RECEPTOR_INTERACTION
DTG.54	KEGG_GRAFT_VERSUS_HOST_DISEASE
DTG.55	KEGG_GALACTOSE_METABOLISM
DTG.56	KEGG_SYSTEMIC_LUPUS_ERYTHEMATOSUS
DTG.57	KEGG_ALLOGRAFT_REJECTION
DTG.58	KEGG_HEMATOPOIETIC_CELL_LINEAGE
DTG.59	KEGG_PARKINSONS_DISEASE
DTG.60	KEGG_INTESTINAL_IMMUNE_NETWORK_FOR_IGA_PRODUCTION
DTG.61	KEGG_PRIMARY_IMMUNODEFICIENCY
DTG.62	KEGG_TYPE_I_DIABETES_MELLITUS
DTG.63	KEGG_CELL_ADHESION_MOLECULES_CAMS
DTG.64	KEGG_OXIDATIVE_PHOSPHORYLATION
DTG.65	KEGG_NICOTINATE_AND_NICOTINAMIDE_METABOLISM
DTG.66	KEGG_CALCIUM_SIGNALING_PATHWAY
DTG.67	KEGG_ALDOSTERONE_REGULATED_SODIUM_REABSORPTION
DTG.68	KEGG_CYTOKINE_CYTOKINE_RECEPTOR_INTERACTION
DTG.69	KEGG_TASTE_TRANSDUCTION
DTG.70	KEGG_VIRAL_MYOCARDITIS
DTG.71	KEGG_FRUCTOSE_AND_MANNOSE_METABOLISM
DTG.72	KEGG_TYPE_II_DIABETES_MELLITUS
DTG.73	KEGG_ANTIGEN_PROCESSING_AND_PRESENTATION
DTG.74	KEGG_JAK_STAT_SIGNALING_PATHWAY
DTG.75	KEGG_PENTOSE_PHOSPHATE_PATHWAY
DTG.76	KEGG_AMYOTROPHIC_LATERAL_SCLEROSIS_ALS
DTG.77	KEGG_O_GLYCAN_BIOSYNTHESIS
DTG.78	KEGG_BASAL_CELL_CARCINOMA
DTG.79	KEGG_REGULATION_OF_AUTOPHAGY
DTG.80	KEGG_SULFUR_METABOLISM
DTG.81	KEGG_HEDGEHOG_SIGNALING_PATHWAY
DTG.82	KEGG_CARDIAC_MUSCLE_CONTRACTION
DTG.83	KEGG_VALINE_LEUCINE_AND_ISOLEUCINE_BIOSYNTHESIS
DTG.84	KEGG_ECM_RECEPTOR_INTERACTION
DTG.85	KEGG_PRION_DISEASES
DTG.86	KEGG_GLYCOSAMINOGLYCAN_DEGRADATION
DTG.87	KEGG_ALZHEIMERS_DISEASE
DTG.88	KEGG_VASCULAR_SMOOTH_MUSCLE_CONTRACTION
DTG.89	KEGG_AMINO_SUGAR_AND_NUCLEOTIDE_SUGAR_METABOLISM

DTG.90	KEGG_SELENOAMINO_ACID_METABOLISM
DTG.91	KEGG_CHEMOKINE_SIGNALING_PATHWAY
DTG.92	KEGG_DILATED_CARDIOMYOPATHY
DTG.93	KEGG_GLYCOSPHINGOLIPID_BIOSYNTHESIS_LACTO_AND_NEOLACTO_SERIES
DTG.94	KEGG_NATURAL_KILLER_CELL_MEDIATED_CYTOTOXICITY
DTG.95	KEGG_GLYCOSPHINGOLIPID_BIOSYNTHESIS_GANGLIO_SERIES
DTG.96	KEGG_ARRHYTHMOGENIC_RIGHT_VENTRICULAR_CARDIOMYOPATHY_ARVC
DTG.97	KEGG_HYPERTROPHIC_CARDIOMYOPATHY_HCM
DTG.98	KEGG_CYTOSOLIC_DNA_SENSING_PATHWAY
DTG.99	KEGG_TOLL_LIKE_RECEPTOR_SIGNALING_PATHWAY
DTG.100	KEGG_HUNTINGTONS_DISEASE
DTG.101	KEGG_LEISHMANIA_INFECTION
DTG.102	KEGG_GLYCOSAMINOGLYCAN_BIOSYNTHESIS_KERATAN_SULFATE
DTG.103	KEGG_SPHINGOLIPID_METABOLISM
DTG.104	KEGG_B_CELL_RECEPTOR_SIGNALING_PATHWAY
DTG.105	KEGG_MELANOGENESIS
DTG.106	KEGG_TGF_BETA_SIGNALING_PATHWAY
DTG.107	KEGG_PROTEASOME
DTG.108	KEGG_T_CELL_RECEPTOR_SIGNALING_PATHWAY
DTG.109	KEGG_MAPK_SIGNALING_PATHWAY
DTG.110	KEGG_FC_EPSILON_RI_SIGNALING_PATHWAY
DTG.111	KEGG_ENDOMETRIAL_CANCER
DTG.112	KEGG_MELANOMA
DTG.113	KEGG_CIRCADIAN_RHYTHM_MAMMAL
DTG.114	KEGG_LEUKOCYTE_TRANSENDOTHELIAL_MIGRATION
DTG.115	KEGG_OTHER_GLYCAN_DEGRADATION
DTG.116	KEGG_LONG_TERM_DEPRESSION
DTG.117	KEGG_GLIOMA
DTG.118	KEGG_RIG_I_LIKE_RECEPTOR_SIGNALING_PATHWAY
DTG.119	KEGG_INSULIN_SIGNALING_PATHWAY
DTG.120	KEGG_GLYCEROPHOSPHOLIPID_METABOLISM
DTG.121	KEGG_GLYCOSPHINGOLIPID_BIOSYNTHESIS_GLOBO_SERIES
DTG.122	KEGG_LONG_TERM_POTENTIATION
DTG.123	KEGG_TIGHT_JUNCTION
DTG.124	KEGG_ACUTE_MYELOID_LEUKEMIA
DTG.125	KEGG_NON_SMALL_CELL_LUNG_CANCER
DTG.126	KEGG_PROTEIN_EXPORT
DTG.127	KEGG_INOSITOL_PHOSPHATE_METABOLISM
DTG.128	KEGG_ETHER_LIPID_METABOLISM
DTG.129	KEGG_RIBOFLAVIN_METABOLISM
DTG.130	KEGG_N_GLYCAN_BIOSYNTHESIS
DTG.131	KEGG_DORSO_VENTRAL_AXIS_FORMATION
DTG.132	KEGG_GLYCOSAMINOGLYCAN_BIOSYNTHESIS_CHONDROITIN_SULFATE
DTG.133	KEGG_ERBB_SIGNALING_PATHWAY
DTG.134	KEGG_FOCAL_ADHESION
DTG.135	KEGG_GLYCOSAMINOGLYCAN_BIOSYNTHESIS_HEPARAN_SULFATE

DTG.136 KEGG_NOD_LIKE_RECEPTOR_SIGNALING_PATHWAY
DTG.137 KEGG_GNRH_SIGNALING_PATHWAY
DTG.138 KEGG_APOPTOSIS
DTG.139 KEGG_GLYCOSYLPHOSPHATIDYLINOSITOL_GPI_ANCHOR_BIOSYNTHESIS
DTG.140 KEGG_SNARE_INTERACTIONS_IN_VESICULAR_TRANSPORT
DTG.141 KEGG_PROSTATE_CANCER
DTG.142 KEGG_PHOSPHATIDYLINOSITOL_SIGNALING_SYSTEM
DTG.143 KEGG_VEGF_SIGNALING_PATHWAY
DTG.144 KEGG_GAP_JUNCTION
DTG.145 KEGG_AXON_GUIDANCE
DTG.146 KEGG_MTOR_SIGNALING_PATHWAY
DTG.147 KEGG_WNT_SIGNALING_PATHWAY
DTG.148 KEGG_RENAL_CELL_CARCINOMA
DTG.149 KEGG_THYROID_CANCER
DTG.150 KEGG_NUCLEOTIDE_EXCISION_REPAIR
DTG.151 KEGG_MISMATCH_REPAIR
DTG.152 KEGG_P53_SIGNALING_PATHWAY
DTG.153 KEGG_COLORECTAL_CANCER
DTG.154 KEGG_AMINOACYL_TRNA_BIOSYNTHESIS
DTG.155 KEGG_REGULATION_OF_ACTIN_CYTOSKELETON
DTG.156 KEGG_BASAL_TRANSCRIPTION_FACTORS
DTG.157 KEGG_FC_GAMMA_R_MEDIATED_PHAGOCYTOSIS
DTG.158 KEGG_NEUROTROPHIN_SIGNALING_PATHWAY
DTG.159 KEGG_PANCREATIC_CANCER
DTG.160 KEGG_LYSOSOME
DTG.161 KEGG_CHRONIC_MYELOID_LEUKEMIA
DTG.162 KEGG_PATHWAYS_IN_CANCER
DTG.163 KEGG_EPITHELIAL_CELL_SIGNALING_IN_HELICOBACTER_PYLORI_INFECTION
DTG.164 KEGG_PROGESTERONE_MEDIATED_OOCYTE_MATURATION
DTG.165 KEGG_RIBOSOME
DTG.166 KEGG_SMALL_CELL_LUNG_CANCER
DTG.167 KEGG_RNA_POLYMERASE
DTG.168 KEGG_ENDOCYTOSIS
DTG.169 KEGG_BASE_EXCISION_REPAIR
DTG.170 KEGG_UBIQUITIN_MEDIATED_PROTEOLYSIS
DTG.171 KEGG_NOTCH_SIGNALING_PATHWAY
DTG.172 KEGG_ADHERENS_JUNCTION
DTG.173 KEGG_VASOPRESSIN_REGULATED_WATER_REABSORPTION
DTG.174 KEGG_BLADDER_CANCER
DTG.175 KEGG_HOMOLOGOUS_RECOMBINATION
DTG.176 KEGG_VIBRIO_CHOLERAE_INFECTION
DTG.177 KEGG_PATHOGENIC_ESCHERICHIA_COLI_INFECTION
DTG.178 KEGG_DNA_REPLICATION
DTG.179 KEGG_RNA_DEGRADATION
DTG.180 KEGG_PURINE_METABOLISM
DTG.181 KEGG_CELL_CYCLE

DTG.182	KEGG_OOCYTE_MEIOSIS
DTG.183	KEGG_NON_HOMOLOGOUS_END_JOINING
DTG.184	KEGG_PYRIMIDINE_METABOLISM
DTG.185	KEGG_SPLICEOSOME

ES	NES	NP	FDR	FWER
-0.7325	-2.2215	0	0	0
-0.736	-2.215	0	3.00E-04	0.001
-0.7381	-2.1487	0	0.0019	0.003
-0.7924	-2.0823	0	0.0031	0.01
-0.835	-2.076	0	0.0027	0.012
-0.6607	-2.0593	0	0.0032	0.016
-0.9171	-2.0374	0	0.0039	0.024
-0.8054	-2.0215	0	0.0043	0.03
-0.6885	-2.0054	0.0075	0.0047	0.039
-0.6195	-1.9674	0.0058	0.0061	0.056
-0.7095	-1.9347	0	0.0073	0.075
-0.6908	-1.816	0.0118	0.0247	0.232
-0.5777	-1.8097	0.0038	0.0241	0.238
-0.552	-1.7978	0.0073	0.0245	0.257
-0.7062	-1.7902	0.0074	0.0252	0.275
-0.5772	-1.7822	0.0057	0.0251	0.289
-0.5387	-1.7819	0.0152	0.0237	0.29
-0.6803	-1.7702	0.0115	0.025	0.317
-0.6441	-1.6905	0.0324	0.0475	0.5
-0.5067	-1.6879	0.0319	0.0463	0.515
-0.7806	-1.654	0.0176	0.0566	0.6
-0.5204	-1.6158	0.0388	0.0708	0.681
-0.4657	-1.5048	0.0551	0.1358	0.875
-0.4326	-1.4759	0.0864	0.1555	0.911
-0.4572	-1.4736	0.0886	0.1516	0.915
-0.3773	-1.4229	0.0503	0.1911	0.945
-0.4924	-1.3816	0.1225	0.2266	0.965
-0.4864	-1.3698	0.1496	0.2309	0.972
-0.3939	-1.3323	0.1451	0.2637	0.98
-0.4802	-1.328	0.1549	0.2599	0.98
-0.4835	-1.3083	0.206	0.2738	0.985
-0.3658	-1.3004	0.1611	0.2741	0.987
-0.2603	-1.2561	0.1593	0.3195	0.994
-0.3789	-1.168	0.2471	0.4285	0.999
-0.3674	-1.1542	0.3109	0.4364	0.999
-0.3419	-1.0781	0.3627	0.5405	0.999
-0.4179	-1.0662	0.3817	0.5435	0.999
-0.3482	-1.0106	0.4533	0.6176	0.999
-0.3639	-1.0088	0.4702	0.6044	0.999
-0.2906	-1.0045	0.4542	0.5956	0.999
-0.326	-1.0034	0.4234	0.5827	0.999
-0.292	-0.9775	0.496	0.6084	0.999
-0.3135	-0.9246	0.5642	0.6768	0.999
-0.299	-0.9092	0.5825	0.6847	0.999

-0.2481	-0.8668	0.6335	0.7345	1
-0.3232	-0.8564	0.5872	0.7347	1
-0.2122	-0.8153	0.7689	0.7814	1
-0.3281	-0.8026	0.6868	0.784	1
-0.199	-0.7404	0.8724	0.8586	1
-0.2764	-0.6689	0.7593	0.936	1
-0.2408	-0.666	0.751	0.9213	1
-0.2107	-0.6368	0.9272	0.9369	1
-0.2479	-0.6367	0.9205	0.9193	1
-0.1438	-0.5941	0.976	0.9431	1
-0.2215	-0.531	0.8764	0.9703	1
-0.1447	-0.4518	0.9961	0.9838	1
0.1383	0.4887	0.9747	0.9708	1
0.2199	0.5298	0.8503	0.9593	1
0.1723	0.5481	0.9297	0.9558	1
0.1689	0.5522	0.9125	0.9609	1
0.2119	0.5586	0.8661	0.9642	1
0.2506	0.598	0.8472	0.9417	1
0.2398	0.6302	0.7872	0.9189	1
0.183	0.6486	0.8477	0.9069	1
0.2045	0.6553	0.8248	0.9072	1
0.204	0.6805	0.8679	0.8853	1
0.1677	0.686	0.9356	0.8861	1
0.1996	0.697	0.8994	0.8794	1
0.1898	0.6992	0.8439	0.8839	1
0.2172	0.7607	0.8349	0.8069	1
0.2392	0.778	0.6827	0.7888	1
0.2325	0.8003	0.7468	0.764	1
0.2294	0.8141	0.7291	0.7514	1
0.2742	0.8166	0.6194	0.7545	1
0.2178	0.8172	0.7124	0.7605	1
0.2599	0.8236	0.6942	0.7583	1
0.2233	0.8536	0.7425	0.7203	1
0.268	0.8674	0.6407	0.7061	1
0.2538	0.8866	0.6626	0.6833	1
0.2614	0.8918	0.6197	0.6818	1
0.3026	0.8964	0.6091	0.6813	1
0.2579	0.9007	0.6092	0.6809	1
0.2607	0.9058	0.5804	0.6798	1
0.3905	0.9127	0.5996	0.6755	1
0.2669	0.917	0.5592	0.6757	1
0.2792	0.9305	0.5466	0.662	1
0.3151	0.9406	0.519	0.6524	1
0.2529	0.9482	0.504	0.6472	1
0.2477	0.9507	0.5069	0.6496	1
0.288	0.9874	0.4456	0.5991	1

0.3178	0.9973	0.498	0.5896	1
0.2792	1.0025	0.4418	0.5874	1
0.2824	1.005	0.4519	0.5898	1
0.3178	1.016	0.4486	0.5796	1
0.2926	1.0165	0.4336	0.5851	1
0.3685	1.0286	0.4403	0.573	1
0.2947	1.0314	0.4046	0.5747	1
0.2899	1.0417	0.3843	0.5647	1
0.3153	1.0597	0.3947	0.5422	1
0.2939	1.0688	0.3985	0.5348	1
0.2935	1.0698	0.3814	0.5397	1
0.358	1.085	0.3825	0.5222	1
0.4079	1.0981	0.374	0.5071	0.999
0.3363	1.1035	0.341	0.5043	0.999
0.3485	1.1204	0.3652	0.4838	0.998
0.2958	1.1371	0.2614	0.464	0.997
0.3256	1.1381	0.313	0.4685	0.997
0.429	1.1481	0.3623	0.4594	0.996
0.3491	1.1482	0.3333	0.4651	0.996
0.2831	1.1541	0.255	0.4624	0.996
0.3211	1.158	0.259	0.4623	0.996
0.3548	1.162	0.3094	0.4624	0.996
0.3084	1.1635	0.2406	0.4665	0.996
0.4794	1.1669	0.3227	0.4674	0.996
0.314	1.1682	0.2892	0.4716	0.996
0.4344	1.1697	0.3183	0.4756	0.996
0.3088	1.1711	0.2185	0.4804	0.996
0.3364	1.1758	0.2777	0.4795	0.996
0.3326	1.1794	0.2739	0.4804	0.996
0.3044	1.1831	0.248	0.4809	0.996
0.2901	1.1927	0.216	0.4722	0.995
0.4295	1.1946	0.245	0.4764	0.995
0.3291	1.1975	0.2213	0.4792	0.993
0.3057	1.2021	0.2151	0.4787	0.993
0.3667	1.2065	0.2618	0.4783	0.993
0.3612	1.2067	0.2633	0.4858	0.993
0.4401	1.2091	0.2679	0.4895	0.992
0.3747	1.2092	0.251	0.4977	0.992
0.3434	1.2223	0.1701	0.4831	0.99
0.4241	1.2312	0.2149	0.4755	0.99
0.3983	1.232	0.2581	0.4828	0.99
0.4142	1.2411	0.204	0.4737	0.989
0.4424	1.247	0.2343	0.4708	0.988
0.3586	1.2475	0.217	0.4789	0.988
0.3444	1.2495	0.2077	0.4842	0.988
0.4214	1.262	0.1815	0.4693	0.986

0.3879	1.2645	0.1952	0.4737	0.986
0.3358	1.2682	0.1482	0.4765	0.985
0.3715	1.275	0.1905	0.473	0.984
0.4446	1.2772	0.1833	0.4791	0.984
0.407	1.2814	0.1886	0.4811	0.984
0.3625	1.2894	0.159	0.4761	0.984
0.3865	1.2965	0.1697	0.4728	0.984
0.3436	1.2985	0.1395	0.4799	0.984
0.3723	1.3088	0.1312	0.469	0.98
0.3571	1.3118	0.1469	0.4739	0.98
0.3994	1.3192	0.1215	0.4693	0.978
0.3467	1.3204	0.1204	0.4787	0.977
0.4002	1.3213	0.1339	0.4893	0.976
0.439	1.34	0.1283	0.4628	0.965
0.4652	1.3516	0.1523	0.4505	0.961
0.5432	1.3635	0.1409	0.4396	0.96
0.3776	1.3737	0.1006	0.4318	0.956
0.4215	1.3783	0.0842	0.4353	0.954
0.4917	1.3808	0.1721	0.4438	0.952
0.3626	1.3852	0.0905	0.4498	0.952
0.4512	1.3888	0.0938	0.4569	0.952
0.4179	1.4027	0.095	0.4441	0.947
0.4001	1.4072	0.0792	0.4502	0.943
0.43	1.4075	0.0707	0.4661	0.943
0.4014	1.4077	0.0994	0.4835	0.943
0.4329	1.4168	0.0772	0.4787	0.938
0.3622	1.4306	0.0323	0.466	0.93
0.4191	1.4509	0.0535	0.4385	0.917
0.4166	1.4717	0.0402	0.4111	0.901
0.7059	1.4778	0.1624	0.4175	0.893
0.4306	1.4837	0.026	0.4256	0.888
0.5292	1.4956	0.0721	0.4178	0.875
0.406	1.5086	0.0219	0.4103	0.862
0.5324	1.5252	0.0787	0.3964	0.84
0.4747	1.5335	0.0182	0.4004	0.827
0.4866	1.5372	0.0302	0.4185	0.823
0.4577	1.5387	0.032	0.4444	0.82
0.4851	1.542	0.0317	0.4712	0.818
0.471	1.5791	0.0155	0.4073	0.748
0.5857	1.5812	0.037	0.4375	0.746
0.4561	1.5825	0.0152	0.4781	0.743
0.4815	1.5881	0.0251	0.5139	0.735
0.6402	1.5959	0.0656	0.5514	0.719
0.5396	1.6054	0.006	0.5927	0.7
0.4065	1.6093	0.0059	0.6707	0.695
0.5243	1.6316	0.0264	0.7003	0.646

0.4555	1.6435	0.002	0.8112	0.618
0.6895	1.6857	0.002	0.8185	0.533
0.476	1.705	0.0077	1	0.494
0.6224	1.8104	0	0.8907	0.262

Supplementary Table S7. Pathways identified in the ICGC HCC cohort

Tag	Term	ES	NES	NP	FDR	FWER
DTG	KEGG_FATTY	-0.7355	-1.9751	0	0.0427	0.049
DTG.1	KEGG_BETA_	-0.6578	-1.9245	0	0.0355	0.075
DTG.2	KEGG_TRYPT	-0.6178	-1.9167	0.0039	0.0252	0.082
DTG.3	KEGG_PRIMA	-0.8799	-1.9017	0	0.0232	0.101
DTG.4	KEGG_PERO	-0.5353	-1.8768	0.0251	0.0265	0.146
DTG.5	KEGG_GLYC	-0.7382	-1.8653	0.0042	0.0238	0.16
DTG.6	KEGG_RETIN	-0.7588	-1.8267	0	0.0305	0.209
DTG.7	KEGG_DRUG	-0.7179	-1.8208	0.0021	0.0278	0.218
DTG.8	KEGG_TYRO	-0.6078	-1.8066	0.0041	0.0279	0.242
DTG.9	KEGG_COMI	-0.6331	-1.7859	0.0241	0.0308	0.275
DTG.10	KEGG_DRUG	-0.6702	-1.7795	0.0176	0.0304	0.293
DTG.11	KEGG_STERO	-0.7135	-1.7714	0.0121	0.0299	0.307
DTG.12	KEGG_HISTII	-0.5648	-1.7338	0.0192	0.039	0.394
DTG.13	KEGG_META	-0.6552	-1.7208	0.0183	0.0407	0.431
DTG.14	KEGG_PPAR	-0.5035	-1.6933	0.035	0.0482	0.509
DTG.15	KEGG_VALIN	-0.5348	-1.6328	0.0649	0.0735	0.648
DTG.16	KEGG_PROP	-0.4963	-1.6115	0.0524	0.0823	0.693
DTG.17	KEGG_LINO	-0.5915	-1.585	0.0176	0.0932	0.752
DTG.18	KEGG_BUTA	-0.5152	-1.5798	0.0449	0.0917	0.76
DTG.19	KEGG_STAR	-0.5411	-1.5448	0.0589	0.1121	0.827
DTG.20	KEGG_PHEN	-0.5547	-1.5084	0.0431	0.1358	0.882
DTG.21	KEGG_ALAN	-0.4634	-1.4923	0.0442	0.1424	0.897
DTG.22	KEGG_LYSIN	-0.4096	-1.4536	0.1294	0.1698	0.925
DTG.23	KEGG_PENT	-0.636	-1.4353	0.1612	0.1794	0.936
DTG.24	KEGG_ASCO	-0.6842	-1.418	0.1503	0.1882	0.946
DTG.25	KEGG_PORP	-0.5018	-1.3661	0.2141	0.2347	0.97
DTG.26	KEGG_FOLA	-0.5637	-1.3626	0.1406	0.2295	0.972
DTG.27	KEGG_ARGII	-0.3661	-1.3295	0.142	0.2577	0.98
DTG.28	KEGG_LIMO	-0.5891	-1.3212	0.1882	0.2573	0.982
DTG.29	KEGG_CYSTI	-0.3708	-1.3207	0.1602	0.2494	0.982
DTG.30	KEGG_ABC_	-0.4418	-1.32	0.1434	0.2421	0.982
DTG.31	KEGG_PYRU	-0.3467	-1.2757	0.1868	0.2793	0.988
DTG.32	KEGG_GLYC	-0.3453	-1.2445	0.1827	0.3067	0.992
DTG.33	KEGG_GLYC	-0.4101	-1.2338	0.1976	0.3098	0.992
DTG.34	KEGG_NITRO	-0.4362	-1.2139	0.2008	0.3231	0.994
DTG.35	KEGG_ADIPO	-0.3098	-1.1799	0.2312	0.3525	0.996
DTG.36	KEGG_CITRA	-0.3764	-1.1765	0.2848	0.3468	0.996
DTG.37	KEGG_PROT	-0.3693	-1.131	0.2827	0.3895	0.998
DTG.38	KEGG_PROX	-0.3964	-1.0969	0.3382	0.4212	0.999
DTG.39	KEGG_SULFU	-0.4332	-1.0889	0.3718	0.4212	0.999
DTG.40	KEGG_ARAC	-0.3567	-1.0771	0.3726	0.4254	0.999
DTG.41	KEGG_ONE_	-0.3246	-1.028	0.4226	0.4762	0.999
DTG.42	KEGG_MATU	-0.3693	-0.9688	0.5051	0.5388	0.999
DTG.43	KEGG_TAUR	-0.3776	-0.9171	0.5728	0.5941	1

DTG.44	KEGG_RIBOS	-0.3117	-0.8784	0.5717	0.6328	1
DTG.45	KEGG_RENIN	-0.294	-0.7398	0.8278	0.8074	1
DTG.46	KEGG_PRIOI	-0.2513	-0.7338	0.7737	0.7985	1
DTG.47	KEGG_INSUI	-0.1533	-0.6661	0.9509	0.8634	1
DTG.48	KEGG_NICO	-0.1948	-0.5874	0.977	0.921	1
DTG.49	KEGG_ASTH	0.2413	0.5572	0.9147	0.9399	1
DTG.50	KEGG_CELL_	0.2063	0.5898	0.9184	0.923	1
DTG.51	KEGG_OLFA	0.1702	0.5904	0.9979	0.9294	1
DTG.52	KEGG_GRAF	0.2874	0.6256	0.8271	0.9051	1
DTG.53	KEGG_TERPI	0.24	0.6335	0.8464	0.904	1
DTG.54	KEGG_REGU	0.1781	0.6613	0.8909	0.8809	1
DTG.55	KEGG_CYTO	0.2324	0.6859	0.8039	0.8582	1
DTG.56	KEGG_VIRAI	0.2441	0.6928	0.7899	0.8562	1
DTG.57	KEGG_TIGHT	0.18	0.6945	0.9379	0.8608	1
DTG.58	KEGG_PANT	0.2633	0.7095	0.8151	0.8486	1
DTG.59	KEGG_LEUK	0.218	0.7154	0.8156	0.8476	1
DTG.60	KEGG_VASC	0.2234	0.7283	0.8519	0.8375	1
DTG.61	KEGG_INTES	0.3218	0.7329	0.7128	0.8376	1
DTG.62	KEGG_ALPH	0.2835	0.7498	0.8307	0.8209	1
DTG.63	KEGG_ENDC	0.1963	0.7521	0.8088	0.8244	1
DTG.64	KEGG_OXID	0.1752	0.755	0.6762	0.8271	1
DTG.65	KEGG_AUTC	0.311	0.768	0.7179	0.8149	1
DTG.66	KEGG_NATU	0.2664	0.774	0.6944	0.8127	1
DTG.67	KEGG_CALC	0.2439	0.775	0.8228	0.8181	1
DTG.68	KEGG_ANTI	0.2757	0.7942	0.6617	0.7965	1
DTG.69	KEGG_ALLO	0.3619	0.807	0.6599	0.7845	1
DTG.70	KEGG_PROT	0.2528	0.8083	0.6889	0.7891	1
DTG.71	KEGG_PRIM	0.3779	0.8406	0.6341	0.747	1
DTG.72	KEGG_MELA	0.2568	0.8507	0.6815	0.7381	1
DTG.73	KEGG_LONC	0.2468	0.8549	0.6896	0.7378	1
DTG.74	KEGG_JAK_S	0.2731	0.855	0.6402	0.7443	1
DTG.75	KEGG_TYPE_	0.3646	0.8663	0.6189	0.7331	1
DTG.76	KEGG_GLIOI	0.2423	0.8678	0.6708	0.7374	1
DTG.77	KEGG_PARK	0.2026	0.8796	0.5776	0.726	1
DTG.78	KEGG_GALA	0.2867	0.88	0.6292	0.7321	1
DTG.79	KEGG_ALDC	0.2825	0.8808	0.6389	0.7377	1
DTG.80	KEGG_NEUR	0.2763	0.8827	0.7121	0.7415	1
DTG.81	KEGG_TYPE_	0.2906	0.883	0.6276	0.7481	1
DTG.82	KEGG_BIOSY	0.3211	0.8917	0.5813	0.7424	1
DTG.83	KEGG_HEMA	0.3524	0.8963	0.56	0.7423	1
DTG.84	KEGG_CYTO	0.3295	0.9037	0.5633	0.7378	1
DTG.85	KEGG_CIRCA	0.364	0.9042	0.5759	0.7442	1
DTG.86	KEGG_CHEM	0.3051	0.9158	0.5415	0.7328	1
DTG.87	KEGG_ADHE	0.2351	0.9161	0.5774	0.7395	1
DTG.88	KEGG_GLYC	0.3572	0.9176	0.5749	0.745	1
DTG.89	KEGG_HEDG	0.3166	0.9311	0.5699	0.7297	1

DTG.90	KEGG_STER(	0.3691	0.9336	0.5361	0.733	1
DTG.91	KEGG_B_CEL	0.3238	0.934	0.5476	0.7399	1
DTG.92	KEGG_FOCA	0.2882	0.9391	0.5335	0.7382	1
DTG.93	KEGG_NOD_	0.3528	0.9488	0.5231	0.7288	1
DTG.94	KEGG_SELEN	0.2707	0.955	0.5128	0.7272	1
DTG.95	KEGG_AMY(	0.2752	0.9574	0.5072	0.731	1
DTG.96	KEGG_SNAR	0.2473	0.9599	0.491	0.7348	1
DTG.97	KEGG_PROS	0.265	0.961	0.503	0.7411	1
DTG.98	KEGG_DORS	0.3618	0.9718	0.5356	0.7306	1
DTG.99	KEGG_TOLL_	0.3276	0.9737	0.5189	0.7355	1
DTG.100	KEGG_RIG_I_	0.2783	0.9817	0.5109	0.7295	1
DTG.101	KEGG_THYR	0.2625	0.9822	0.4782	0.7371	1
DTG.102	KEGG_GNRH	0.2925	0.9888	0.4908	0.7338	1
DTG.103	KEGG_GLUT	0.2693	0.9963	0.4576	0.7291	1
DTG.104	KEGG_HUNT	0.1865	0.9968	0.501	0.7373	1
DTG.105	KEGG_T_CEL	0.3467	0.9993	0.4865	0.7416	1
DTG.106	KEGG_LEISH	0.4056	1.0121	0.5052	0.7277	1
DTG.107	KEGG_BASA	0.3503	1.018	0.4297	0.7266	1
DTG.108	KEGG_ERBB_	0.2768	1.0195	0.4632	0.7334	1
DTG.109	KEGG_MTOR	0.2923	1.0245	0.4399	0.7328	1
DTG.110	KEGG_GLYC	0.293	1.0253	0.4352	0.7412	1
DTG.111	KEGG_FC_EF	0.3379	1.0328	0.4388	0.7362	1
DTG.112	KEGG_TGF_E	0.3194	1.0387	0.3996	0.7335	1
DTG.113	KEGG_GLYC	0.3196	1.0403	0.4249	0.7401	1
DTG.114	KEGG_APOF	0.3076	1.0403	0.4343	0.7503	1
DTG.115	KEGG_RNA_	0.3096	1.0461	0.4177	0.7496	1
DTG.116	KEGG_DILAT	0.3713	1.0489	0.4215	0.7548	1
DTG.117	KEGG_NEUR	0.2776	1.0506	0.4392	0.762	1
DTG.118	KEGG_MELA	0.3267	1.0718	0.3972	0.7268	0.999
DTG.119	KEGG_NOT(	0.3562	1.073	0.4119	0.7352	0.999
DTG.120	KEGG_AMIN	0.3197	1.0748	0.3925	0.7427	0.999
DTG.121	KEGG_ACUT	0.3052	1.0851	0.3932	0.7323	0.999
DTG.122	KEGG_ECM_	0.399	1.0936	0.3987	0.7265	0.999
DTG.123	KEGG_OTHE	0.3388	1.1031	0.3261	0.7165	0.999
DTG.124	KEGG_HYPE	0.3813	1.1048	0.3765	0.7244	0.999
DTG.125	KEGG_VALIN	0.4167	1.1053	0.3409	0.7351	0.999
DTG.126	KEGG_ALZH	0.2353	1.1104	0.341	0.7363	0.999
DTG.127	KEGG_GLYC	0.3282	1.1108	0.294	0.7476	0.999
DTG.128	KEGG_SPHIN	0.3308	1.1119	0.2965	0.7585	0.999
DTG.129	KEGG_NON_	0.3204	1.1195	0.346	0.7549	0.999
DTG.130	KEGG_REGU	0.3096	1.1202	0.3171	0.7667	0.999
DTG.131	KEGG_N_GL	0.3042	1.1437	0.3395	0.7266	0.999
DTG.132	KEGG_GLYC	0.3921	1.147	0.2733	0.7325	0.999
DTG.133	KEGG_GAP_	0.3614	1.1522	0.2836	0.7338	0.999
DTG.134	KEGG_VASC	0.2946	1.1566	0.277	0.7374	0.999
DTG.135	KEGG_MAPK	0.316	1.1575	0.303	0.7495	0.998

DTG.136	KEGG_VIBRI(	0.3078	1.1638	0.2992	0.7497	0.998
DTG.137	KEGG_WNT_	0.3282	1.1688	0.2562	0.7536	0.998
DTG.138	KEGG_VEGF_	0.3542	1.1753	0.3044	0.753	0.998
DTG.139	KEGG_FRUC	0.3735	1.1853	0.2871	0.7435	0.997
DTG.140	KEGG_ARRH	0.4003	1.1878	0.2516	0.7539	0.997
DTG.141	KEGG_EPITH	0.3376	1.1899	0.3008	0.7649	0.997
DTG.142	KEGG_INOS	0.3974	1.1906	0.286	0.7801	0.997
DTG.143	KEGG_ENDC	0.2961	1.1947	0.2617	0.7873	0.997
DTG.144	KEGG_TASTI	0.4272	1.1972	0.2012	0.799	0.997
DTG.145	KEGG_PHOS	0.3932	1.1996	0.2825	0.8125	0.997
DTG.146	KEGG_PATH	0.3301	1.2058	0.2484	0.8163	0.997
DTG.147	KEGG_PENT	0.3813	1.2131	0.2409	0.8138	0.997
DTG.148	KEGG_GLYC	0.4502	1.2135	0.2328	0.8344	0.997
DTG.149	KEGG_GLYC	0.3794	1.217	0.2168	0.8462	0.996
DTG.150	KEGG_BASA	0.3724	1.2279	0.2449	0.8364	0.996
DTG.151	KEGG_LONG	0.3673	1.2334	0.1722	0.8431	0.995
DTG.152	KEGG_BASE_	0.3701	1.2386	0.2609	0.8515	0.994
DTG.153	KEGG_AXON	0.3907	1.2575	0.1913	0.8174	0.991
DTG.154	KEGG_FC_G/	0.4235	1.2592	0.263	0.8367	0.991
DTG.155	KEGG_ETHEI	0.4395	1.2624	0.1652	0.854	0.99
DTG.156	KEGG_SYSTE	0.42	1.2629	0.1946	0.8806	0.99
DTG.157	KEGG_COLC	0.3624	1.2671	0.1979	0.8962	0.99
DTG.158	KEGG_AMIN	0.3519	1.2702	0.2157	0.9168	0.989
DTG.159	KEGG_CHRC	0.3549	1.2802	0.2188	0.9151	0.988
DTG.160	KEGG_PURII	0.3021	1.3024	0.1051	0.8716	0.985
DTG.161	KEGG_PANC	0.3939	1.3234	0.1762	0.8298	0.982
DTG.162	KEGG_PATH	0.3749	1.324	0.1694	0.8618	0.982
DTG.163	KEGG_RENA	0.4132	1.325	0.2009	0.8955	0.981
DTG.164	KEGG_CARD	0.4129	1.3401	0.1174	0.8787	0.977
DTG.165	KEGG_GLYC	0.5486	1.3411	0.1557	0.9156	0.977
DTG.166	KEGG_GLYC	0.4942	1.3585	0.0956	0.8921	0.974
DTG.167	KEGG_PYRIN	0.3256	1.3744	0.1374	0.8756	0.97
DTG.168	KEGG_BLAD	0.4174	1.3839	0.0994	0.8845	0.968
DTG.169	KEGG_SMAL	0.4261	1.3899	0.109	0.909	0.966
DTG.170	KEGG_PROG	0.4211	1.4074	0.0962	0.8853	0.962
DTG.171	KEGG_SPLIC	0.3321	1.4095	0.1414	0.934	0.959
DTG.172	KEGG_UBIQ	0.3102	1.415	0.1523	0.9712	0.953
DTG.173	KEGG_RIBOI	0.5436	1.435	0.0888	0.9483	0.943
DTG.174	KEGG_RNA_	0.3376	1.4392	0.1219	1	0.94
DTG.175	KEGG_GLYC	0.5703	1.461	0.0649	0.979	0.926
DTG.176	KEGG_NUCL	0.4105	1.4814	0.1165	0.9624	0.913
DTG.177	KEGG_OOCY	0.4073	1.5248	0.0247	0.8251	0.874
DTG.178	KEGG_P53_S	0.443	1.5334	0.0458	0.8787	0.863
DTG.179	KEGG_LYSO	0.3619	1.5407	0.0695	0.9559	0.852
DTG.180	KEGG_O_GL	0.5716	1.5519	0.0283	1	0.837
DTG.181	KEGG_DNA_	0.6059	1.5549	0.0938	1	0.835

DTG.182	KEGG_MISM	0.586	1.6225	0.0423	0.9592	0.722
DTG.183	KEGG_NON_	0.6199	1.6491	0.018	1	0.655
DTG.184	KEGG_HOM	0.643	1.6682	0.0184	1	0.613
DTG.185	KEGG_CELL_	0.5816	1.8336	0.0062	0.6004	0.212

Supplementary Table S8. Pathways identified in the GSE15654 cohort

Tag	Term	ES	NES	NP	FDR	FWER
DTG	KEGG_RIBOS	-0.6728	-2.2112	0	0	0
DTG.1	KEGG_VALIN	-0.7161	-2.1792	0	6.00E-04	0.001
DTG.2	KEGG_PERO	-0.6397	-2.1246	0	0.002	0.004
DTG.3	KEGG_META	-0.634	-2.1111	0.002	0.0018	0.004
DTG.4	KEGG_DRUG	-0.6573	-2.1035	0.002	0.0015	0.004
DTG.5	KEGG_ARGI	-0.6287	-2.0967	0	0.0015	0.005
DTG.6	KEGG_COMI	-0.6873	-2.0745	0	0.0015	0.006
DTG.7	KEGG_FATTY	-0.7126	-2.0686	0	0.0013	0.006
DTG.8	KEGG_BUTA	-0.6769	-2.0671	0	0.0011	0.006
DTG.9	KEGG_TRYPT	-0.642	-2.0345	0	0.0015	0.008
DTG.10	KEGG_PYRU	-0.6281	-2.0248	0	0.0016	0.009
DTG.11	KEGG_LYSIN	-0.591	-1.9799	0	0.0032	0.023
DTG.12	KEGG_PROP	-0.6802	-1.9795	0	0.0029	0.023
DTG.13	KEGG_LINO	-0.6301	-1.9459	0	0.0049	0.036
DTG.14	KEGG_RETIN	-0.6004	-1.9436	0.0061	0.0047	0.038
DTG.15	KEGG_GLYC	-0.6936	-1.9313	0.002	0.0053	0.041
DTG.16	KEGG_HISTI	-0.592	-1.8626	0.002	0.0109	0.088
DTG.17	KEGG_GLYC	-0.5038	-1.8493	0.004	0.0116	0.099
DTG.18	KEGG_TYRO	-0.5296	-1.8486	0.0041	0.011	0.1
DTG.19	KEGG_CITRA	-0.6256	-1.8306	0.0099	0.0127	0.118
DTG.20	KEGG_LIMO	-0.8226	-1.8247	0.002	0.0129	0.126
DTG.21	KEGG_DRUG	-0.5619	-1.8222	0.0041	0.0129	0.132
DTG.22	KEGG_AMIN	-0.5132	-1.8073	0.0057	0.0151	0.16
DTG.23	KEGG_PARK	-0.4492	-1.8058	0.004	0.0148	0.163
DTG.24	KEGG_RNA_	-0.4873	-1.801	0.0038	0.0149	0.174
DTG.25	KEGG_BETA_	-0.6303	-1.8008	0.0126	0.0143	0.174
DTG.26	KEGG_GLUT	-0.4917	-1.7999	0.0041	0.0139	0.174
DTG.27	KEGG_PRIM	-0.7128	-1.7966	0.0081	0.0139	0.181
DTG.28	KEGG_OXID	-0.4495	-1.7841	0	0.015	0.2
DTG.29	KEGG_ABC_	-0.4689	-1.7839	0.0039	0.0145	0.2
DTG.30	KEGG_HUNT	-0.3945	-1.7804	0	0.0142	0.203
DTG.31	KEGG_PHEN	-0.629	-1.7692	0.0061	0.0155	0.219
DTG.32	KEGG_ALAN	-0.5349	-1.7674	0.0061	0.0151	0.22
DTG.33	KEGG_CYSTI	-0.5238	-1.7205	0.0077	0.0223	0.305
DTG.34	KEGG_PENT	-0.5906	-1.7016	0.0261	0.0256	0.344
DTG.35	KEGG_STER	-0.5053	-1.6975	0.0164	0.0257	0.349
DTG.36	KEGG_PROT	-0.5178	-1.6935	0.022	0.0259	0.358
DTG.37	KEGG_ASCO	-0.6112	-1.6914	0.0228	0.0258	0.364
DTG.38	KEGG_SPLIC	-0.4059	-1.6906	0.002	0.0254	0.365
DTG.39	KEGG_PATH	-0.5089	-1.6842	0.0291	0.0264	0.382
DTG.40	KEGG_BIOSY	-0.5999	-1.6812	0.0223	0.0265	0.391
DTG.41	KEGG_PROT	-0.5442	-1.6725	0.0079	0.0279	0.411
DTG.42	KEGG_PORP	-0.5013	-1.6478	0.0224	0.0344	0.477
DTG.43	KEGG_GLYC	-0.5692	-1.6458	0.0283	0.034	0.48

DTG.44	KEGG_ONE_	-0.5605	-1.6155	0.0405	0.0422	0.544
DTG.45	KEGG_PANT	-0.5457	-1.6128	0.0225	0.0423	0.548
DTG.46	KEGG_SELEI	-0.4841	-1.5658	0.0409	0.0594	0.666
DTG.47	KEGG_ALZH	-0.349	-1.5652	0.008	0.0584	0.667
DTG.48	KEGG_PPAR	-0.4239	-1.5591	0.0385	0.0596	0.682
DTG.49	KEGG_UBIQ	-0.3718	-1.5479	0.0139	0.0631	0.704
DTG.50	KEGG_SYSTE	-0.3449	-1.475	0.0323	0.1004	0.835
DTG.51	KEGG_TERPI	-0.5263	-1.4464	0.0669	0.1171	0.867
DTG.52	KEGG_RNA_	-0.4381	-1.4005	0.0655	0.1518	0.921
DTG.53	KEGG_STAR	-0.4008	-1.4004	0.0751	0.1491	0.921
DTG.54	KEGG_VIBRI	-0.3594	-1.3938	0.0602	0.1523	0.924
DTG.55	KEGG_PYRIN	-0.3101	-1.3478	0.058	0.1938	0.953
DTG.56	KEGG_ARAC	-0.3375	-1.3345	0.0781	0.2042	0.956
DTG.57	KEGG_BASA	-0.3874	-1.3319	0.0875	0.2037	0.957
DTG.58	KEGG_NUCL	-0.386	-1.3257	0.1235	0.2076	0.96
DTG.59	KEGG_SULFI	-0.4704	-1.2775	0.1582	0.2642	0.975
DTG.60	KEGG_AMIN	-0.3385	-1.2684	0.1159	0.2725	0.978
DTG.61	KEGG_PROS	-0.3082	-1.2638	0.1137	0.2745	0.979
DTG.62	KEGG_INSUI	-0.2821	-1.2609	0.0974	0.2744	0.98
DTG.63	KEGG_DORS	-0.3721	-1.2559	0.1357	0.2772	0.983
DTG.64	KEGG_PRIOI	-0.3799	-1.2463	0.2037	0.2863	0.986
DTG.65	KEGG_ADHE	-0.3196	-1.2367	0.1918	0.2951	0.987
DTG.66	KEGG_LYSO	-0.2697	-1.2152	0.1175	0.3219	0.992
DTG.67	KEGG_NOD_	-0.3185	-1.192	0.2081	0.3536	0.996
DTG.68	KEGG_ETHEI	-0.3641	-1.1898	0.2105	0.352	0.996
DTG.69	KEGG_THYR	-0.3474	-1.1817	0.2399	0.3591	0.996
DTG.70	KEGG_LEISH	-0.3036	-1.1808	0.2045	0.3552	0.996
DTG.71	KEGG_GALA	-0.3416	-1.1622	0.269	0.3827	0.998
DTG.72	KEGG_VASC	-0.2719	-1.1535	0.2023	0.3932	0.998
DTG.73	KEGG_NITR	-0.3634	-1.1489	0.2851	0.3956	0.998
DTG.74	KEGG_ANTI	-0.2867	-1.1383	0.2722	0.4093	0.998
DTG.75	KEGG_MTOI	-0.2966	-1.1362	0.2571	0.408	0.998
DTG.76	KEGG_ALLO	-0.3385	-1.1172	0.3157	0.4357	0.998
DTG.77	KEGG_ALPH	-0.3749	-1.1064	0.2971	0.4502	0.998
DTG.78	KEGG_GLYC	-0.294	-1.0978	0.3112	0.4598	0.998
DTG.79	KEGG_TYPE_	-0.3286	-1.0961	0.3301	0.4575	0.998
DTG.80	KEGG_CELL_	-0.2621	-1.0911	0.3314	0.4614	0.998
DTG.81	KEGG_NICO	-0.3444	-1.0841	0.3295	0.4695	0.999
DTG.82	KEGG_GRAF	-0.3227	-1.0715	0.3845	0.4888	0.999
DTG.83	KEGG_CYTO	-0.2791	-1.0673	0.3791	0.4904	0.999
DTG.84	KEGG_PANC	-0.2658	-1.0671	0.3259	0.485	0.999
DTG.85	KEGG_SMAL	-0.264	-1.0626	0.3464	0.4881	0.999
DTG.86	KEGG_BLAD	-0.2903	-1.0573	0.3412	0.492	0.999
DTG.87	KEGG_FOCA	-0.2411	-1.0563	0.3556	0.4882	0.999
DTG.88	KEGG_ASTH	-0.3181	-1.0536	0.3895	0.4878	0.999
DTG.89	KEGG_INOS	-0.2736	-1.0379	0.3976	0.514	0.999

DTG.90	KEGG_EPITH	-0.2554	-1.0333	0.4104	0.5162	0.999
DTG.91	KEGG_NON_	-0.2584	-1.0296	0.409	0.518	0.999
DTG.92	KEGG_ACUT	-0.2636	-1.0282	0.3996	0.5153	0.999
DTG.93	KEGG_ADIPO	-0.249	-1.0219	0.425	0.5218	0.999
DTG.94	KEGG_ECM_	-0.2642	-1.0193	0.4233	0.5208	0.999
DTG.95	KEGG_PROX	-0.3386	-1.0156	0.4277	0.5226	0.999
DTG.96	KEGG_ENDO	-0.2202	-1.0143	0.4156	0.5198	0.999
DTG.97	KEGG_GLYC	-0.3229	-1.0128	0.4451	0.5175	0.999
DTG.98	KEGG_GLYC	-0.2958	-1.0051	0.4536	0.5269	0.999
DTG.99	KEGG_VIRAL	-0.2651	-1.0034	0.4362	0.5249	0.999
DTG.100	KEGG_ENDO	-0.2474	-0.9976	0.466	0.5307	0.999
DTG.101	KEGG_FOLA	-0.3864	-0.99	0.4527	0.5389	0.999
DTG.102	KEGG_JAK_S	-0.2086	-0.9688	0.5456	0.5747	1
DTG.103	KEGG_LONG	-0.2414	-0.9567	0.503	0.5935	1
DTG.104	KEGG_FC_GA	-0.2259	-0.9417	0.5476	0.6172	1
DTG.105	KEGG_TOLL_	-0.2174	-0.9414	0.5622	0.6119	1
DTG.106	KEGG_LONG	-0.2293	-0.9292	0.5924	0.6306	1
DTG.107	KEGG_PHOS	-0.2279	-0.926	0.5843	0.6309	1
DTG.108	KEGG_RENA	-0.2235	-0.9254	0.6075	0.6261	1
DTG.109	KEGG_OTHE	-0.3025	-0.908	0.5926	0.6552	1
DTG.110	KEGG_MATU	-0.2977	-0.8971	0.583	0.6699	1
DTG.111	KEGG_CIRCA	-0.3555	-0.8819	0.6048	0.694	1
DTG.112	KEGG_STERO	-0.3072	-0.8677	0.6499	0.7154	1
DTG.113	KEGG_PURIN	-0.1772	-0.8267	0.874	0.7891	1
DTG.114	KEGG_SNAR	-0.2348	-0.8239	0.7535	0.7877	1
DTG.115	KEGG_B_CEL	-0.2047	-0.812	0.7725	0.8031	1
DTG.116	KEGG_HEMA	-0.1923	-0.799	0.8238	0.8181	1
DTG.117	KEGG_RENIN	-0.2877	-0.796	0.7526	0.8166	1
DTG.118	KEGG_CHRO	-0.1978	-0.7856	0.8528	0.8275	1
DTG.119	KEGG_TGF_B	-0.1985	-0.7849	0.8085	0.8223	1
DTG.120	KEGG_GLYC	-0.1897	-0.7652	0.8701	0.8458	1
DTG.121	KEGG_TAUR	-0.3037	-0.765	0.7573	0.8391	1
DTG.122	KEGG_ERBB_	-0.1764	-0.7478	0.9303	0.8566	1
DTG.123	KEGG_NOTC	-0.2033	-0.7407	0.8828	0.8595	1
DTG.124	KEGG_NON_	-0.2589	-0.7239	0.8676	0.8736	1
DTG.125	KEGG_INTES	-0.2041	-0.7209	0.8821	0.8701	1
DTG.126	KEGG_VALIN	0.1988	0.5466	0.9548	0.9837	1
DTG.127	KEGG_GLYC	0.1772	0.5576	0.985	0.997	1
DTG.128	KEGG_AUTO	0.1943	0.7044	0.865	0.9196	1
DTG.129	KEGG_CHEM	0.1576	0.7177	0.9841	0.9206	1
DTG.130	KEGG_T_CEL	0.181	0.751	0.8757	0.893	1
DTG.131	KEGG_P53_S	0.1893	0.7511	0.9018	0.9091	1
DTG.132	KEGG_DNA_	0.2177	0.7559	0.841	0.9184	1
DTG.133	KEGG_GLYC	0.2282	0.7593	0.8281	0.9301	1
DTG.134	KEGG_MISM	0.2626	0.8106	0.7579	0.8565	1
DTG.135	KEGG_BASE_	0.2268	0.8171	0.7838	0.861	1

DTG.136	KEGG_RIG_I_	0.2117	0.8274	0.72	0.8565	1
DTG.137	KEGG_VASC	0.2231	0.8516	0.7287	0.8232	1
DTG.138	KEGG_N_GL	0.227	0.8591	0.6987	0.8237	1
DTG.139	KEGG_PENT	0.2515	0.86	0.6982	0.8393	1
DTG.140	KEGG_PRIM	0.2681	0.867	0.6151	0.8416	1
DTG.141	KEGG_FC_E	0.2027	0.8677	0.728	0.8587	1
DTG.142	KEGG_GLYC	0.2937	0.8689	0.636	0.8755	1
DTG.143	KEGG_CARD	0.2246	0.8816	0.676	0.865	1
DTG.144	KEGG_HOM	0.2775	0.882	0.5973	0.8846	1
DTG.145	KEGG_REGU	0.1903	0.9145	0.6763	0.8226	1
DTG.146	KEGG_NEUR	0.209	0.9185	0.6143	0.8324	1
DTG.147	KEGG_FRUC	0.2666	0.9348	0.5595	0.8114	1
DTG.148	KEGG_GAP_	0.2232	0.9392	0.5558	0.822	1
DTG.149	KEGG_GNRH	0.2154	0.9424	0.5845	0.8351	1
DTG.150	KEGG_CELL_	0.2192	0.9501	0.5342	0.8357	1
DTG.151	KEGG_GLYC	0.3358	0.9521	0.5169	0.8542	1
DTG.152	KEGG_CYTO	0.205	0.9561	0.5356	0.868	1
DTG.153	KEGG_NATU	0.2202	0.9597	0.5258	0.8844	1
DTG.154	KEGG_OOCY	0.2212	0.9599	0.5466	0.9117	1
DTG.155	KEGG_RIBO	0.3479	0.9779	0.499	0.8842	1
DTG.156	KEGG_REGU	0.2962	0.9832	0.47	0.8953	1
DTG.157	KEGG_GLIO	0.2456	0.992	0.4676	0.8982	1
DTG.158	KEGG_WNT_	0.2248	1.0101	0.4385	0.8717	1
DTG.159	KEGG_AXON	0.2264	1.0232	0.4157	0.8605	1
DTG.160	KEGG_SPHIN	0.3016	1.0377	0.4131	0.8448	1
DTG.161	KEGG_VEGF_	0.2433	1.0384	0.3723	0.8766	1
DTG.162	KEGG_PATH	0.2058	1.0395	0.3354	0.909	1
DTG.163	KEGG_AMY	0.2795	1.0405	0.4089	0.9451	1
DTG.164	KEGG_ALDO	0.3	1.0445	0.404	0.9722	1
DTG.165	KEGG_ARRH	0.2575	1.0581	0.3478	0.965	1
DTG.166	KEGG_GLYC	0.376	1.0808	0.3558	0.9286	0.999
DTG.167	KEGG_TASTE	0.29	1.0874	0.3082	0.9515	0.999
DTG.168	KEGG_APOP	0.2627	1.1033	0.2977	0.942	0.999
DTG.169	KEGG_MAPK	0.2278	1.1177	0.1873	0.9407	0.999
DTG.170	KEGG_COLO	0.2749	1.1258	0.2596	0.9656	0.999
DTG.171	KEGG_TIGHT	0.2513	1.1268	0.256	1	0.999
DTG.172	KEGG_O_GL	0.3527	1.1271	0.2965	1	0.999
DTG.173	KEGG_CALC	0.2562	1.1463	0.2163	1	0.996
DTG.174	KEGG_GLYC	0.3541	1.1537	0.2595	1	0.996
DTG.175	KEGG_MELA	0.2825	1.211	0.1354	0.9494	0.992
DTG.176	KEGG_HYPE	0.3198	1.2591	0.1446	0.8269	0.984
DTG.177	KEGG_PROG	0.2969	1.2593	0.1164	0.918	0.984
DTG.178	KEGG_TYPE_	0.3578	1.2815	0.1279	0.9233	0.974
DTG.179	KEGG_DILAT	0.3235	1.2875	0.1355	1	0.97
DTG.180	KEGG_BASA	0.344	1.2974	0.1012	1	0.966
DTG.181	KEGG_LEUK	0.2993	1.3012	0.0893	1	0.964

DTG.182	KEGG_MELA	0.335	1.322	0.0778	1	0.956
DTG.183	KEGG_NEUR	0.3446	1.4566	0.035	0.908	0.854
DTG.184	KEGG_HEDG	0.4099	1.5653	0.0098	0.6351	0.666
DTG.185	KEGG_OLFA	0.3878	1.5974	0.0215	1	0.6
